# Computational investigation of the effect of BODIPY labelling on peptide-membrane interaction

**DOI:** 10.1038/s41598-024-72662-y

**Published:** 2024-11-12

**Authors:** Dominique de Jong-Hoogland, Jacob Ulmschneider, Martin Ulmschneider

**Affiliations:** 1 Department of Chemistry, King’s College London, London, UK; 2 Institute of Natural Sciences, Shanghai Jiao Tong University, Shanghai, China

**Keywords:** Molecular dynamic simulations, Membrane active peptide, BODIPY, deltaG, Computational chemistry, Computational biophysics

## Abstract

Optical monitoring of peptide binding to live cells is hampered by the abundance of naturally occurring fluorophores such as tryptophan. Unnatural amino acids incorporating synthetic fluorophores such as BODIPY overcome these optical limitations. A drawback to using fluorophores in lipid binding peptide design is their propensity to override other interactions, potentially causing the peptides to lose their binding selectivity. Here, the binding strength of a selection of peptides incorporating a variety of BODIPY derivatized amino acids has been studied via molecular dynamics simulations to quantify the impact of BODIPY incorporation on peptide-membrane binding behaviour.

## Introduction

Molecular dynamics (MD) simulations have proven a useful tool in elucidating the modes of action of membrane active peptides (MAPs)^1–3^ as well as in informing the design of new sequences^4–7^. Using all-atom MD (AA-MD) methodologies a good agreement between simulation and physical experiments with model membranes has previously been achieved^8^. However, such model membranes fall short of accurately representing the complexity of biological membranes^9–11^. To further test the utility of MD as a design tool for MAPs alongside physical experiments, matching it to *in vivo* measurements is crucial. Currently, only secondary observations such as the therapeutic effect (cell death *vs* survival) are used when evaluating the binding ability of peptides to live cells. This impedes direct comparison between simulation and experiment and slows the development of cell membrane targeting peptides.

To overcome this, solvatochromic compounds can be incorporated into the peptide design to allow for observation of membrane adsorption of the peptide on live cells, analogous to the tryptophan assay employed with model membranes^12^. First synthesised in the 1960s, BODIPY derivatives have become widely used in a myriad of applications ranging from biological labelling^13–15^ to solid-state solar concentrators^16,17^ on account of its excellent chemical stability and photophysical properties. For biomedical applications its most valued properties are its excitation and emission wavelengths which lie in the visible spectral region^18^. These properties can be altered through introduction of chemical groups to the BODIPY core, of which numerous examples are present in the literature. For an overview of various functionalised BODIPYs the interested reader is referred to reviews by Boens *et al*. and Loudet and Brugess^18,19^.

**Fig. 1:**
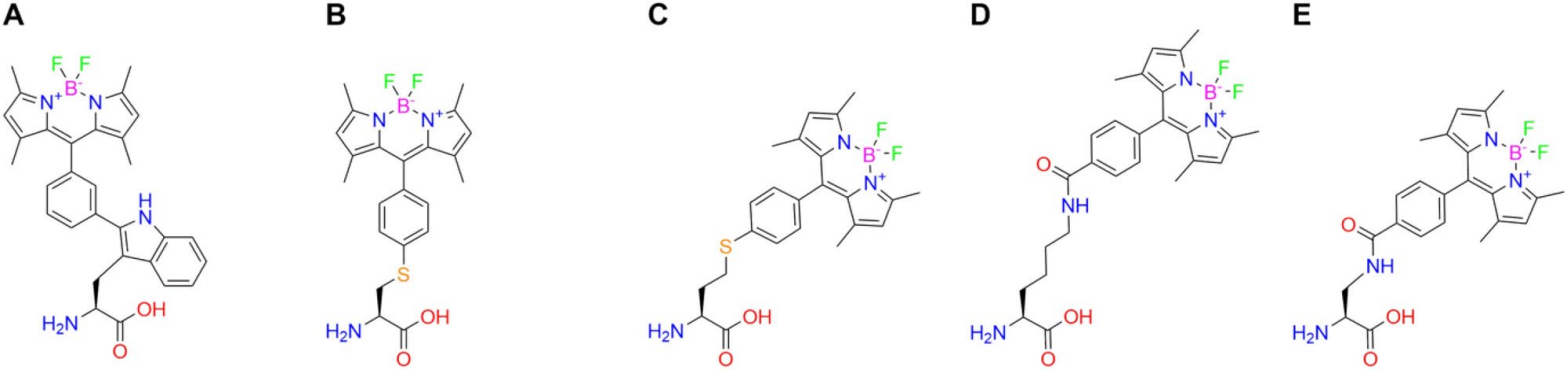
Molecular structures of BODIPY functionalised amino acids: (**A**) tryptophan (WB), (**B**) cysteine (CB), (**C**) homocysteine (hCB), (**D**) lysine (KB) and (**E**) 3-amino-alanine (DPB).

Often when fluorophores are incorporated into peptides, they are linked through either the N- or C-terminus which is known to interfere with the activity of cell active peptides^20,21^. Connecting the fluorophore via the side chain of an amino acid could help to overcome this problem and would offer greater flexibility in position along the peptide chain. One compound that meets these criteria is a BODIPY functionalised tryptophan developed by Mendive-Tapia *et al.*^22,23^. This compound shows a significant increase in emission intensity when exposed to a more hydrophobic environment^22^. Here, use has been made of AA-MD to quantify the impact of BODIPY functionalised amino acids on the membrane binding behaviour of membrane active peptides. The effects of rigidity, connectivity, and distance of the BODIPY moiety to the peptide backbone have been assessed through use of tryptophan, cysteine, lysine, homocysteine and 3-amino-alanine as BODIPY bearing residues (see Fig. 1). Additionally, the impact of incorporation near the N- and C-terminus has been determined.

## Results

Unbiased, atomic detail, multi-microsecond partitioning simulations have been performed of four host peptide sequences, six guest amino acids, and two model membranes representing a eukaryotic (100% POPC lipids) and a prokaryotic (70% POPC and 30% POPG lipids) cell respectively. These membrane compositions are chosen as in previous studies they have shown good agreement between in silico and wet-lab experiments^24–26^. The peptide sequences are seven amino acids in length and contain lipophilic and anionic residues alongside the tryptophan or BODIPY-bearing residue. They are predicted to be too short to form a secondary structure so only membrane partitioning and no subsequent peptide folding is expected to be observed.

**Fig. 2:**
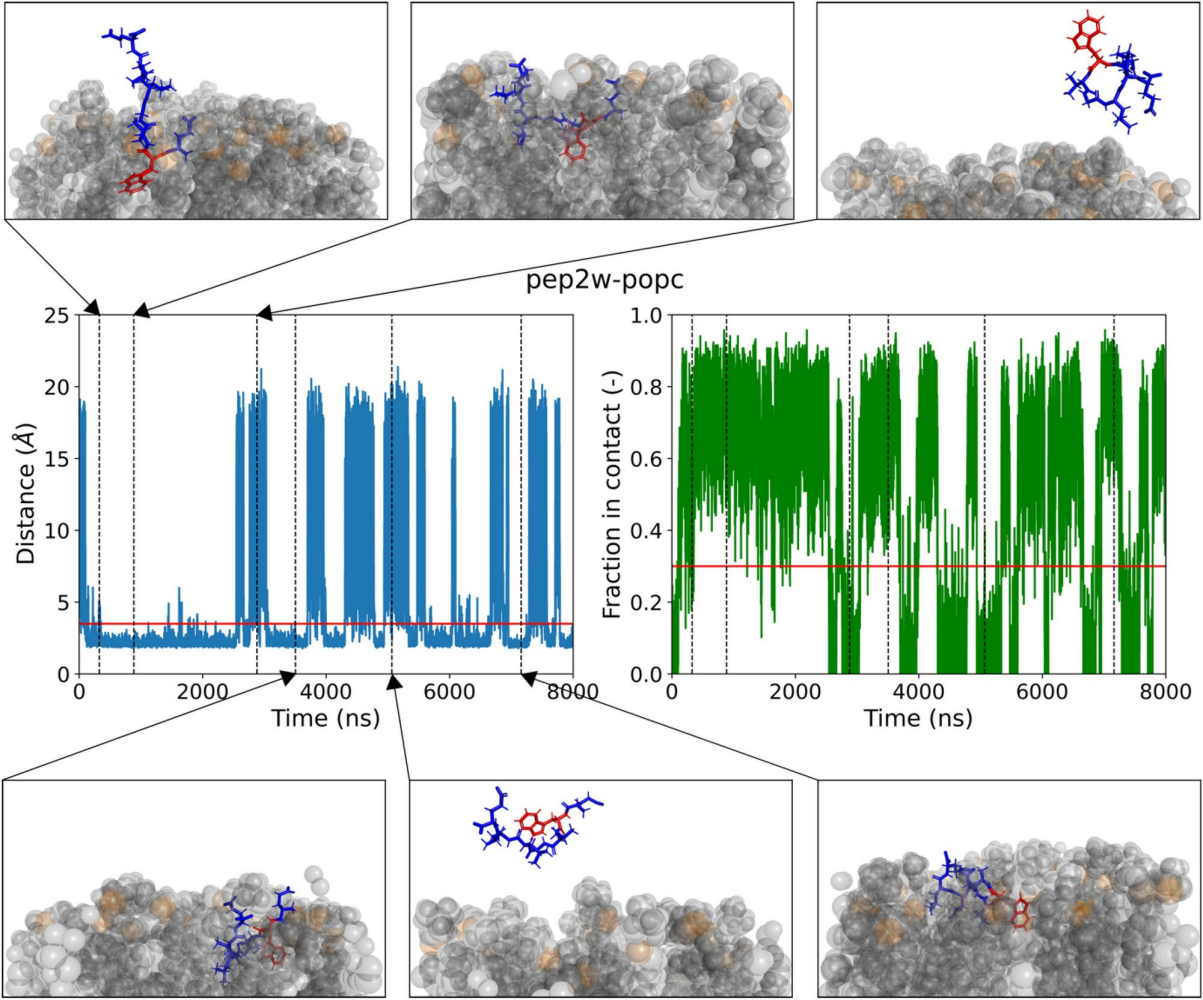
Trajectory of pep2 with the tryptophan guest residue and the POPC membrane. Central panels: in blue the average minimum distance of each residue to the membrane for each frame and in green the corresponding fraction of the peptide that is considered in contact with the membrane. In the left panel the red horizontal line depicts the cut-off value below which the peptide is considered within distance from the membrane. In the right panel the red horizontal line depicts the cut-off value of the fraction above which the peptide is considered bound to the membrane. Black vertical depict frames corresponding to trajectory screen shots in the upper and lower panel.

**Fig. 3:**
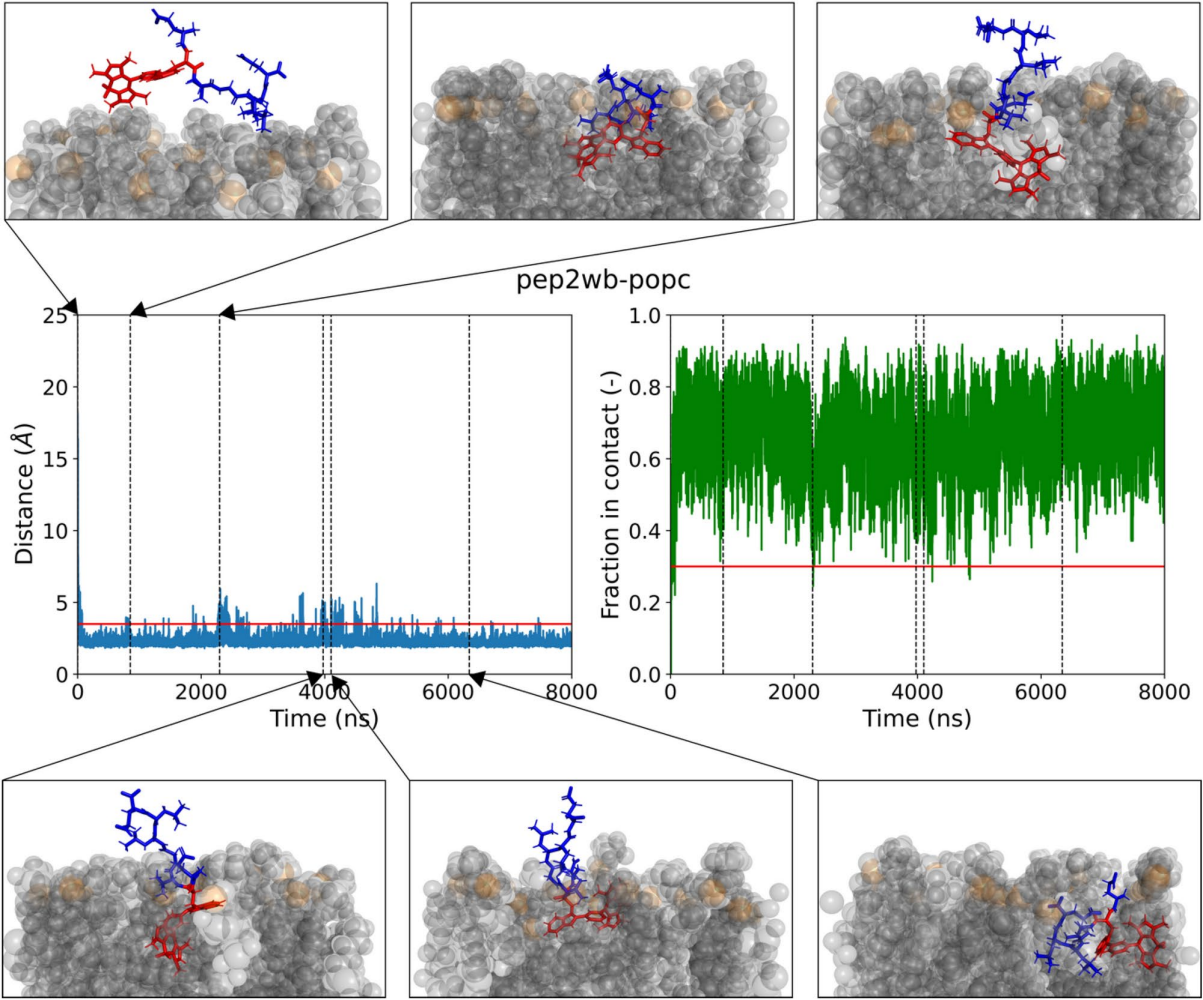
Trajectory of pep2 with the BODIPY bearing tryptophan guest residue and the POPC membrane. Central panels: in blue the average minimum distance of each residue to the membrane for each frame and in green the corresponding fraction of the peptide that is considered in contact with the membrane. In the left panel the red horizontal line depicts the cut-off value below which the peptide is considered within distance from the membrane. In the right panel the red horizontal line depicts the cut-off value of the fraction above which the peptide is considered bound to the membrane. Black vertical depict frames corresponding to trajectory screen shots in the upper and lower panel.

The guest amino acid is incorporated in either the second or penultimate position. The sequences studied are EXDLPLE (pep1), EXVGLLE (pep2), EDLPLXE (pep3) and EVGLLXE (pep4) where X denotes the location of the tryptophan or BODIPY-labelled residue. Sequences pep1 and pep2 were previously developed in the Ulsmchneider lab and in physical experiments have shown moderate binding affinity for POPC/POPG and pure POPC large unilammelar vesicles (LUVs) respectively (unpublished data). Pep3 and pep4 were derived from these sequences to evaluate the effect of incorporating the fluorophore at the C-terminus and have not been tested in physical experiments. When determining the affinity of the peptides for a membrane the fraction of the peptide in contact with the membrane, number of binding events, total time spend bound, and the deltaG are considered. The peptide is considered to be in contact with the membrane when the minimum distance is less than 3.5 Å and this is true for at least 30% of the atoms in the peptide. The fraction requirement was determined based on a visual investigation of trajectories containing tryptophan and tryptophan-BODIPY amino acids. This is illustrated in Figs. 2 and 3, which show the peptide is completely loose from the membrane (Fig. 2 upper right and lower middle panel and Fig. 3 upper left panel) or in contact with a single residue (Fig. 3 upper right, lower left and middle panel) when neither criteria is met, when both criteria are met multiple residues in the peptide are embedded within the membrane (Fig. 2 upper middle, lower left and lower right panel and Fig. 3 upper middle and lower right panel).

**Fig. 4:**
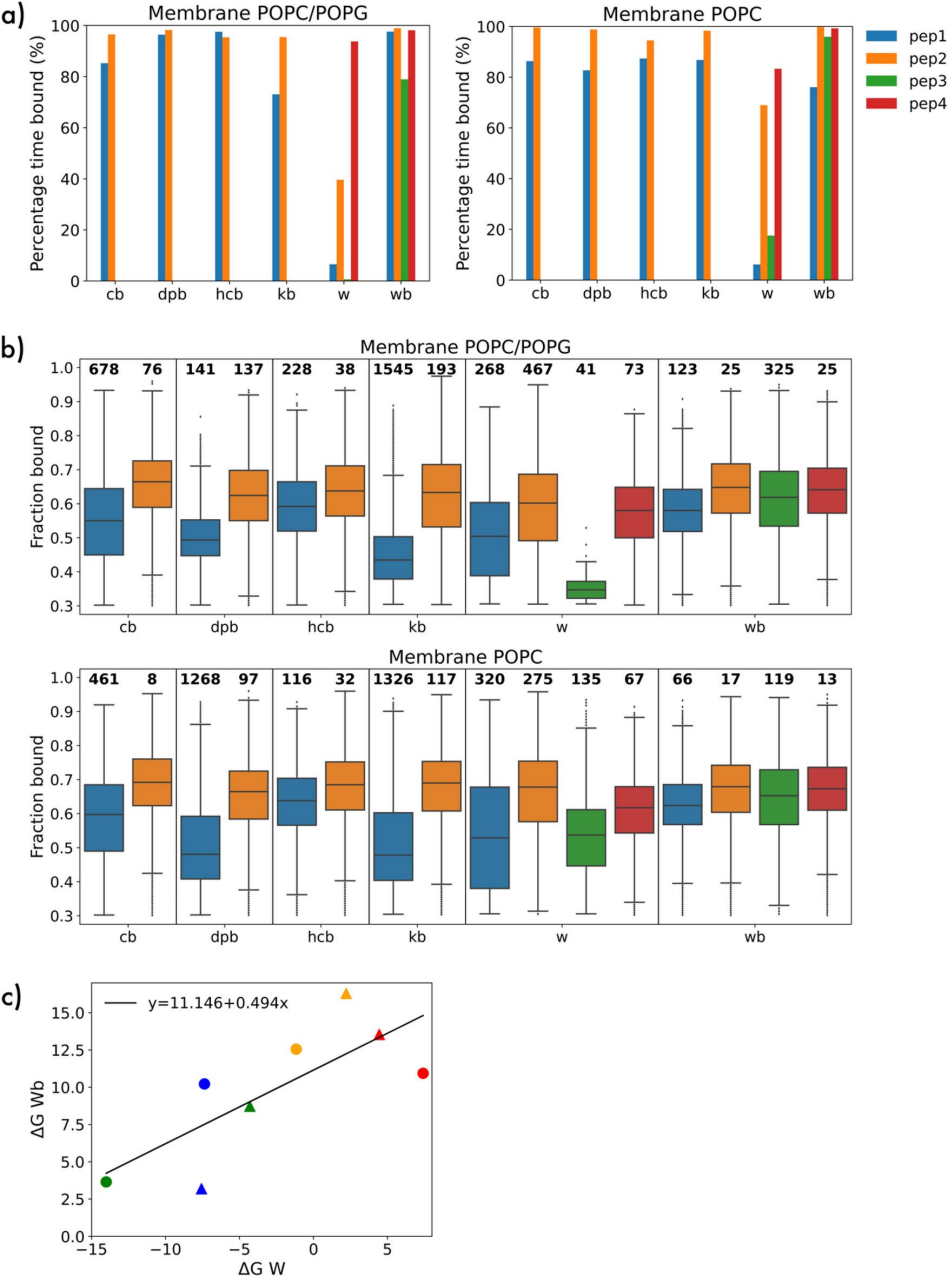
Data corresponding to pep1 is given in blue, pep2 in orange, pep3 in green and pep4 in red. (**a**) Bar plot representation of the percentage of simulated time the peptide is considered in the bound state with the 70/30 POPC/POPG membrane in the left panel and the POPC membrane in the right panel. (**b**) Boxplot representation of distribution of peptide fraction in contact with the membrane when the peptide is considered bound. The number above each boxplot gives the number of observations included in the boxplot. The upper panel represents the peptides interacting with the 70/30 POPC/POPG membrane and the lower panel represents the POPC membrane. (**c**) Scatter plot showing the deltaG of tryptophan containing peptides on the x-axis and tryptophan-BODIPY containing peptides on the y-axis. Data points corresponding to the POPC membrane are shown as triangles, those corresponding to the POPC/POPG membrane as circles. Linear regression fit of the data with R^2^ = 0.59, slope 95% c.i. = 0.494 ± 0.414 and intercept 95% c.i. = 11.146 ± 2.953.

In MD simulations the tryptophan containing peptides show the following binding behaviour; pep1 and pep3 show limited affinity for either membrane with a high number of binding and unbinding events and less than 20% of the time spend in contact with either membrane. This is a deviation from previously conducted physical experiments where for pep1 a slight preference for binding to the POPC/POPG membrane was found. Pep2 shows preferential binding to the POPC membrane spending around 75% of the time in contact with this membrane and around 40% with the POPC/POPG membrane, this is in accordance with previously conducted physical experiments where a slight preference for binding to the POPC membrane was observed. When examining the total number of contacts for pep2 with the lipids in the POPC/POPG membrane we find there is indeed slightly less preference for interaction with the POPG lipid, 25% of contacts involve POPG while this lipid makes up 30% of the membrane (see supporting information Figure S35). Pep4 exhibits the strongest affinity for both membranes with the fewest binding and unbinding events and over 80% of the time spend in contact with both membranes (see Fig. 4a). These behaviours are reflected in the deltaGs which are calculated according to:

1 $$\Delta G = \frac{{ - RT\; {\text{ln}}\left( {\frac{1}{{t_{b} /\left( {t_{b} + t_{l} } \right)}} - 1} \right)}}{1000}$$

where T is the temperature in Kelvin, R is the gasconstant, t_b_ is the total simulated time the peptide is bound to the membrane and t_l_ is the total simulated time the peptide is loose from the membrane resulting in the deltaG in kcal/mol. Pep1 and pep3 have a negative deltaG for both membranes which is of similar value for pep1 regardless of the membrane (− 7.566 kcal/mol for POPC and − 7.369 kcal/mol for POPC/POPG) and a threefold larger for pep3 upon interaction with the POPC/POPG membrane (− 14.011 kcal/mol rather than − 4.298 kcal/mol for POPC). Pep2 shows a positive deltaG for interaction with POPC of 2.204 kcal/mol and a slightly negative deltaG upon interaction with the POPC/POPG membrane of − 1.174 kcal/mol. Pep4 shows positive values of deltaG for interaction with both membranes with 4.447 kcal/mol for POPC and 7.490 kcal/mol for POPC/POPG (see Table 1 and Fig. 4c). When looking at the distribution of the fraction of the peptide in contact with the membrane when the bound state is assigned a clear distinction is seen between pep1 and pep3, and pep2 and pep4 (see Fig. 4b). For both membranes pep1 and pep3 have on average a smaller fraction that is in contact than pep2 and pep4, with the smallest fraction in contact for pep3 binding to POPC/POPG with an average around 35%. Based on minimum residue distances over time (RMSD), radius of gyration (Rgyr), solvent accessible surface analysis (SASA) and Ramachandran plots (supporting information Figures S1-S8 h)-l)) it can be concluded, as expected, that the peptides do not form a secondary structure neither when in the bound nor unbound state.

**Table 1:** DeltaGs of whole peptides in kcal/mol as calculated from MD simulations.

Peptide	Guest amino acid	POPC/POPG membrane	POPC membrane
pep1	CB	4.853	5.089
	DPB	9.098	4.331
	hCB	10.184	5.349
	KB	2.757	5.198
	W	− 7.369	− 7.566
	WB	10.225	3.193
pep2	CB	9.158	15.043
	DPB	11.062	12.186
	hCB	8.379	7.862
	KB	8.436	11.180
	W	− 1.174	2.204
	WB	12.553	16.292
pep3	W	− 14.011	− 4.298
	WB	3.654	8.723
pep4	W	7.490	4.447
	WB	10.936	13.538

When the guest amino acid is changed to the BODIPY functionalised tryptophan all peptides show strong affinity for both membranes with a large drop in binding and unbinding events, over 80% of the simulation time spend in contact with the membrane (see Fig. 4a) and positive deltaGs for all peptide-membrane interactions (see Table 1 and Fig. 4c). Pep1 shows the least affinity for the POPC membrane with the highest number of events and the least amount of time spend in contact with the membrane and a deltaG of 3.193 kcal/mol. For the POPC/POPG membrane pep3 shows the smallest affinity with a deltaG of 3.654 kcal/mol. This result illustrates the loss of selectivity observed in peptide-membrane interactions upon introduction of a fluorophore. For pep1 and pep2 the simulations where repeated with the other BODIPY bearing amino acids. All show behaviour that is similar to what is observed for sequences where the BODIPY moiety is connected through tryptophan with a stark drop in binding events compared to the native peptide and over 80% of the time spend in contact with the membrane (see Fig. 4a). For all guest amino acids, apart from hCB containing sequences, pep2 exhibits a larger deltaG than pep1 and the original preference of pep2 for the POPC membrane is also largely preserved upon introduction of the fluorophore (see Table 1). When examining the number of contacts between the individual residues and the membrane (see supporting information Figures S41-S64) it becomes apparent that the increased number of contacts between the peptides and the membranes is driven by the BODIPY-labelled residues. For the POPC/POPG membranes the percentage of interactions with each individual lipid correspond to the membrane composition showing the BODIPY fluorophore has no preference for binding to either POPC or POPG.

There does not seem to be a preferred orientation of the BODIPY moiety within the membrane (illustrated by upper and lower panels of Fig. 3). It’s position along the z-axis of the simulation box appears to be determined by the rigidity and length of the residue which connects it to the peptide back bone (see supporting information Figures S73-96).

Using the deltaGs, the effect of BODIPY introduction into the peptide design is quantified. To this end the deltaGs of all peptide sequences containing tryptophan and tryptophan-BODIPY residues were compared, and a linear relationship was fitted through these datapoints (see Fig. 4c). From this analysis it follows that ΔΔG irrespective of membrane is 11.146 ± 2.953 kcal/mol.

**Fig. 5:**
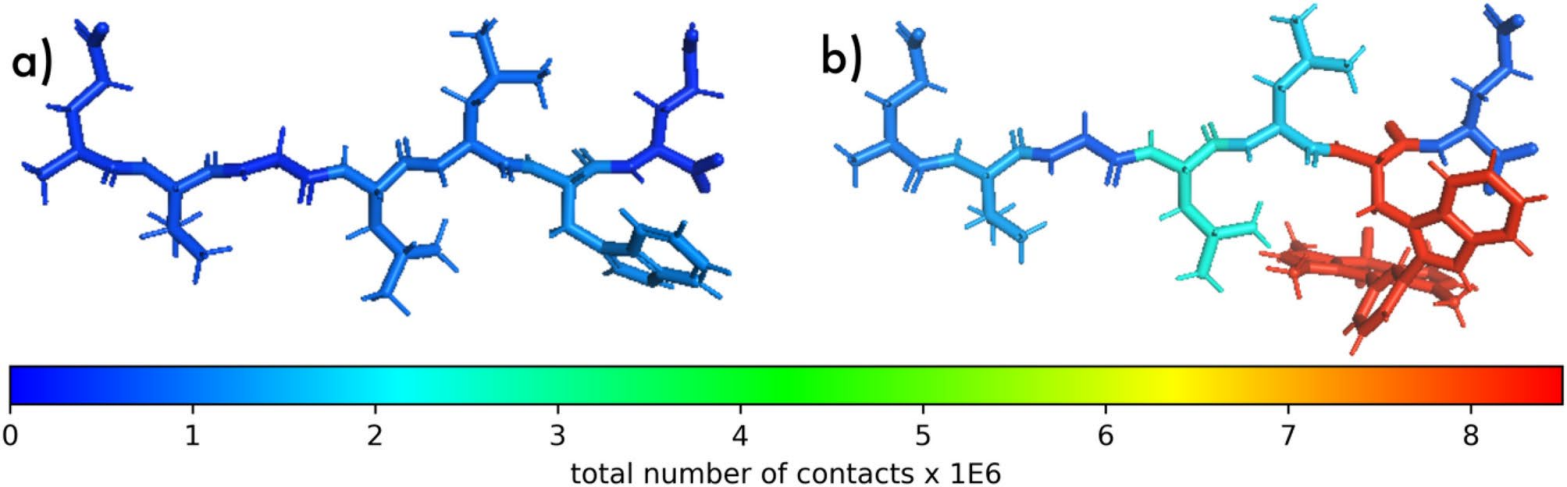
Structural representations with each residue coloured according to the number of contacts made with the POPC membrane for (**a**) pep4 with Tryptophan guest residue. (**b**) pep4 with Tryptophan-BODIPY guest residue.

All combinations of peptide sequence and BODIPY bearing amino acid show a much larger fraction of the peptide in contact with the membrane than the parent tryptophan containing sequence (see Fig. 4b). This implies that it is not just the BODIPY entity that binds with the membranes, but other residues are also pulled into contact with the membrane. Analysing the number of contacts for each residue with the membrane indeed shows neighbouring residues also have an increased number of interactions with the membrane (see Fig. 5).

## Materials and methods

### Parameters for new amino acids

To simulate the various BODIPY functionalised amino acids a combination of CHARMM-GUI Ligand Reader & Modeler and parameters from previous publications was used to construct the topologies and new amino acids were added to the CHARMM36 database (see supporting information)^27–30^.

### MD simulations

For these simulations two model membranes consisting of 60 lipids were used, one representing a eukaryotic cell containing solely POPC lipids and one representing a prokaryotic cell containing 70% POPC lipids and 30% POPG lipids. The membranes were constructed and equilibrated using the CHARMM-GUI membrane builder tool^27^. Peptide membrane simulations were set-up with the peptide at a distance of 15 Å from the membrane. All simulations are performed at an elevated temperature of 333K to speed up the dynamics and ensure the simulations converged.

With position restrains in place on the peptide, an energy minimization step with steepest descent integrator to a minimal of 1000.0 kJ/mol/nm with a Verlet cut-off scheme was conducted. This was followed by a 10 ps equilibration step with a step size of 0.1 fs for 100.000 steps at 333K with NVT ensemble using V-rescale thermostat on the whole system and Berendsen pressure coupling^31,32^. Finally, a longer equilibration step lasting 20 ns was performed with a step size of 1 fs for 20.000.000 steps at 333K with NPT ensemble using V-rescale thermostat and Berendsen pressure coupling.

Production runs were performed for 8 µs in the NPT ensemble with a target pressure of 1 bar and temperature of 333 K using the Berendsen barostat and independent temperature coupling on the peptide, membrane and solvent. The position restriction on the peptide was removed for the production run. LINCS constraints on all bonds were used^33^. A Verlet list cut-off scheme was used for the non-bonded interactions^34^. The van der Waals and Coulomb interactions were cut-off at 1.0 nm. Long-range electrostatic effects were treated with the particle-mesh Ewald method^35^.

### Analysis

To determine the SASA of each trajectory the GROMACS function gmx sasa was used, for all other analysis use was made of the MDAnalysis python package^36–39^.

The state of the peptide, bound or unbound, was determined based on the proximity of a certain fraction of atoms in the peptide to the membrane for a minimal number of consecutive frames. An atom is regarded as bound when its distance to the membrane is 3.5 angstrom or less. At least 30% of the atoms in the peptide need to be within this binding distance for at least 50 consecutive time steps (100 fs) in order for the peptide to be assigned the bound state.

The deltaG was determined from the simulation through the following relationship $\Delta G= -RT \;ln\left(\frac{1}{\frac{{t}_{b}}{{t}_{b}+{t}_{l}}-1}\right).$ Where T is the temperature in Kelvin, R is the gas constant, t_b_ is the simulated time bound in ns and t_l_ is the simulated time unbound in ns.

## Conclusions

The ability to optically monitor peptide binding to the plasma membrane will streamline the process through which new MAPs are designed. The introduction of fluorophores to peptides to enable this often affects the binding ability or specificity of the peptide. Here a computational studies was undertaken to quantify the impact of the introduction of a BODIPY fluorophore on peptide-membrane interactions. To this end four peptide sequences, five BODIPY-labelled amino acids, and two model membranes were employed. MD simulations show the introduction of the BODIPY fluorophore into a peptide sequence greatly impacts the binding propensity of the peptide compared to a tryptophan analogues sequence. This effect is observed regardless of the amino acid chosen as the carrier of the BODIPY moiety. Although the presence of the fluorophore biases the peptides to the bound state, the value of the deltaG varies between peptides indicating that the peptide sequence continues to affect the binding behaviour. It is shown that the contact time between neighbouring residues and the membrane is increased upon introduction of the fluorophore. It may therefore be possible to modulate the binding behaviour of the peptide through careful selection of residues neighbouring the fluorophore.

## Supplementary Information

Supplementary Information.

## Data Availability

Supporting information is available for this manuscript.
