# Supplementary material for: Computational investigation of the effect of BODIPY labelling on peptide-membrane interaction
Source: Sci Rep. 2024 Nov 12;14:27726. doi: 10.1038/s41598-024-72662-y (PMC11557973; doi:10.1038/s41598-024-72662-y)
Supplement: Supplementary file 1 — Supplementary Information. [file 41598_2024_72662_MOESM1_ESM.pdf]

Supporting Information - Computational Investigation of the Effect of BODIPY Labelling on Peptide-Membrane Interaction

Adapting forcefield.

Topologies of BODIPY containing peptides were described in the CHARMM36 forcefield. Parameters for the BODIPY core were as previously published[1], the parameters for atoms connecting the BODIPY moiety to the natural amino acids were determined using the CHARMM-GUI Ligand Reader & Modeler tool[2,3]. Standard CHARMM36 parameters were used for the amino acids.

When adding these parameters to the forcefield it is important to follow the strict layout of the files with respect to alignment, blank spaces and tabs.

Topology of the BODIPY amino acids added to merged.rtp

```
[ CYB ]
  [ atoms ]
        N    NH1   -0.470  0
       HN      H    0.310  1
       CA    CT1    0.070  2
       HA    HB1    0.090  3
       CB    CT2   -0.110  4
      HB1    HA2    0.090  5
      HB2    HA2    0.090  6
       SG  SG311   -0.230  7
       CP CG2R61   -0.061  8
      CQ1 CG2R61   -0.114  9
      HQ1  HGR61    0.115 10
      CR1 CG2R61   -0.114 11
      HR1  HGR61    0.115 12
      CS2 CG2R61   -0.001 13
      CR2 CG2R61   -0.114 14
      HR2  HGR61    0.115 15
      CQ2 CG2R61   -0.114 16
      HQ2  HGR61    0.115 17
      CT2 CG2R67    0.219 18
      CU1  CG2O9    0.090 19
      CT1  CG2O9   -0.180 20
      CS1  CG331   -0.080 21
     HS11   HGA3    0.080 22
     HS12   HGA3    0.080 23
     HS13   HGA3    0.080 24
      CV1  CG2O8   -0.290 25
      HV1  HGR63    0.160 26
      CX1  CG2O9    0.100 27
      CY1  CG331   -0.080 28
     HY11   HGA3    0.080 29
     HY12   HGA3    0.080 30
     HY13   HGA3    0.080 31
      NW1  NG2D1   -0.200 32
      FY1   FGA2   -0.400 33
       BX     BL    0.799 34
      FY2   FGA2   -0.400 35
      NW2  NG2D1   -0.200 36
      CX2  CG2O9    0.100 37
      CY2  CG331   -0.080 38
     HY21   HGA3    0.080 39
     HY22   HGA3    0.080 40
     HY23   HGA3    0.080 41
      CV2  CG2O8   -0.290 42
      HV2  HGR63    0.160 43
      CT3  CG2O9   -0.180 44
      CS3  CG331   -0.080 45
```

```
      HS31   HGA3    0.080 46
      HS32   HGA3    0.080 47
      HS33   HGA3    0.080 48
       CU2  CG2O9    0.090 49
         C      C    0.510 50
         O      O   -0.510 51
 [ bonds ]
        CB     CA
        SG     CB
         N     HN
         N     CA
         C     CA
         C     +N
        CA     HA
        CB    HB1
        CB    HB2
        SG     CP
        CP    CQ1
       CQ1    HQ1
       CQ1    CR1
       CR1    HR1
       CR1    CS2
       CS2    CT2
       CT2    CU1
       CU1    CT1
       CT1    CS1
       CS1   HS11
       CS1   HS12
       CS1   HS13
       CT1    CV1
       CV1    HV1
       CV1    CX1
       CX1    CY1
       CY1   HY11
       CY1   HY12
       CY1   HY13
       CX1    NW1
       NW1    CU1
       NW1     BX
        BX    FY1
        BX    FY2
        BX    NW2
       NW2    CU2
       NW2    CX2
       CX2    CY2
       CY2   HY21
       CY2   HY22
       CY2   HY23
       CX2    CV2
       CV2    HV2
       CV2    CT3
       CT3    CS3
       CS3   HS31
       CS3   HS32
       CS3   HS33
       CT3    CU2
       CU2    CT2
       CS2    CR2
       CR2    HR2
       CR2    CQ2
       CQ2    HQ2
```

```
       CQ2    CP
         O     C
  [ impropers ]
         N    -C    CA    HN
         C    CA    +N     O
  [ cmap ]
        -C     N    CA     C    +N

[ DPB ]
  [ atoms ]
         N    NH1   -0.470  0
        HN      H    0.310  1
        CA    CT1    0.070  2
        HA    HB1    0.090  3
        CB    CT2    0.001  4
       HB1    HA2    0.090  5
       HB2    HA2    0.090  6
        NG    NH2   -0.565  7
        HG     HC    0.334  8
        CD  CG2O1    0.405  9
        OD  OG2D1   -0.459 10
        CP CG2R61   -0.117 11
       CQ1 CG2R61   -0.114 12
       HQ1  HGR61    0.115 13
       CR1 CG2R61   -0.114 14
       HR1  HGR61    0.115 15
       CS2 CG2R61   -0.001 16
       CR2 CG2R61   -0.114 17
       HR2  HGR61    0.115 18
       CQ2 CG2R61   -0.114 19
       HQ2  HGR61    0.115 20
       CT2 CG2R67    0.219 21
       CU1  CG2O9    0.090 22
       CT1  CG2O9   -0.180 23
       CS1  CG331   -0.080 24
      HS11   HGA3    0.080 25
      HS12   HGA3    0.080 26
      HS13   HGA3    0.080 27
       CV1  CG2O8   -0.290 28
       HV1  HGR63    0.160 29
       CX1  CG2O9    0.100 30
       CY1  CG331   -0.080 31
      HY11   HGA3    0.080 32
      HY12   HGA3    0.080 33
      HY13   HGA3    0.080 34
       NW1  NG2D1   -0.200 35
       FY1   FGA2   -0.400 36
        BX     BL    0.799 37
       FY2   FGA2   -0.400 38
       NW2  NG2D1   -0.200 39
       CX2  CG2O9    0.100 40
       CY2  CG331   -0.080 41
      HY21   HGA3    0.080 42
      HY22   HGA3    0.080 43
      HY23   HGA3    0.080 44
       CV2  CG2O8   -0.290 45
       HV2  HGR63    0.160 46
       CT3  CG2O9   -0.180 47
       CS3  CG331   -0.080 48
      HS31   HGA3    0.080 49
      HS32   HGA3    0.080 50
```

```
     HS33   HGA3    0.080 51
      CU2  CG2O9    0.090 52
        C      C    0.510 53
        O      O   -0.510 54
 [ bonds ]
       CB     CA
       NG     CB
        N     HN
        N     CA
        C     CA
        C     +N
       CA     HA
       CB    HB1
       CB    HB2
       NG     HG
       NG     CD
       CD     OD
       CD     CP
       CP    CQ1
      CQ1    HQ1
      CQ1    CR1
      CR1    HR1
      CR1    CS2
      CS2    CT2
      CT2    CU1
      CU1    CT1
      CT1    CS1
      CS1   HS11
      CS1   HS12
      CS1   HS13
      CT1    CV1
      CV1    HV1
      CV1    CX1
      CX1    CY1
      CY1   HY11
      CY1   HY12
      CY1   HY13
      CX1    NW1
      NW1    CU1
      NW1     BX
       BX    FY1
       BX    FY2
       BX    NW2
      NW2    CU2
      NW2    CX2
      CX2    CY2
      CY2   HY21
      CY2   HY22
      CY2   HY23
      CX2    CV2
      CV2    HV2
      CV2    CT3
      CT3    CS3
      CS3   HS31
      CS3   HS32
      CS3   HS33
      CT3    CU2
      CU2    CT2
      CS2    CR2
      CR2    HR2
      CR2    CQ2
```

```
      CQ2   HQ2
      CQ2    CP
        O     C
  [ impropers ]
        N    -C    CA    HN
        C    CA    +N     O
  [ cmap ]
       -C     N    CA     C    +N

[ HCB ]
  [ atoms ]
        N    NH1   -0.470  0
       HN      H    0.310  1
       CA    CT1    0.070  2
       HA    HB1    0.090  3
       CB    CT2   -0.180  4
      HB1    HA2    0.090  5
      HB2    HA2    0.090  6
       CG    CT2   -0.110  7
      HG1    HA2    0.090  8
      HG2    HA2    0.090  9
       SD  SG311   -0.230 10
       CP CG2R61   -0.061 11
      CQ1 CG2R61   -0.114 12
      HQ1  HGR61    0.115 13
      CR1 CG2R61   -0.114 14
      HR1  HGR61    0.115 15
      CS2 CG2R61   -0.001 16
      CR2 CG2R61   -0.114 17
      HR2  HGR61    0.115 18
      CQ2 CG2R61   -0.114 19
      HQ2  HGR61    0.115 20
      CT2 CG2R67    0.219 21
      CU1  CG2O9    0.090 22
      CT1  CG2O9   -0.180 23
      CS1  CG331   -0.080 24
     HS11   HGA3    0.080 25
     HS12   HGA3    0.080 26
     HS13   HGA3    0.080 27
      CV1  CG2O8   -0.290 28
      HV1  HGR63    0.160 29
      CX1  CG2O9    0.100 30
      CY1  CG331   -0.080 31
     HY11   HGA3    0.080 32
     HY12   HGA3    0.080 33
     HY13   HGA3    0.080 34
      NW1  NG2D1   -0.200 35
      FY1   FGA2   -0.400 36
       BX     BL    0.799 37
      FY2   FGA2   -0.400 38
      NW2  NG2D1   -0.200 39
      CX2  CG2O9    0.100 40
      CY2  CG331   -0.080 41
     HY21   HGA3    0.080 42
     HY22   HGA3    0.080 43
     HY23   HGA3    0.080 44
      CV2  CG2O8   -0.290 45
      HV2  HGR63    0.160 46
      CT3  CG2O9   -0.180 47
      CS3  CG331   -0.080 48
     HS31   HGA3    0.080 49
```

```
     HS32   HGA3    0.080 50
     HS33   HGA3    0.080 51
      CU2  CG2O9    0.090 52
        C      C    0.510 53
        O      O   -0.510 54
[ bonds ]
       CB    CA
       CG    CB
       SD    CG
        N    HN
        N    CA
        C    CA
        C    +N
       CA    HA
       CB   HB1
       CB   HB2
       CG   HG1
       CG   HG2
       SD    CP
       CP   CQ1
      CQ1   HQ1
      CQ1   CR1
      CR1   HR1
      CR1   CS2
      CS2   CT2
      CT2   CU1
      CU1   CT1
      CT1   CS1
      CS1  HS11
      CS1  HS12
      CS1  HS13
      CT1   CV1
      CV1   HV1
      CV1   CX1
      CX1   CY1
      CY1  HY11
      CY1  HY12
      CY1  HY13
      CX1   NW1
      NW1   CU1
      NW1    BX
       BX   FY1
       BX   FY2
       BX   NW2
      NW2   CU2
      NW2   CX2
      CX2   CY2
      CY2  HY21
      CY2  HY22
      CY2  HY23
      CX2   CV2
      CV2   HV2
      CV2   CT3
      CT3   CS3
      CS3  HS31
      CS3  HS32
      CS3  HS33
      CT3   CU2
      CU2   CT2
      CS2   CR2
      CR2   HR2
```

```
       CR2   CQ2
       CQ2   HQ2
       CQ2    CP
         O     C
  [ impropers ]
         N    -C    CA    HN
         C    CA    +N     O
  [ cmap ]
        -C     N    CA     C    +N

[ LYB ]
  [ atoms ]
         N    NH1   -0.470  0
        HN      H    0.310  1
        CA    CT1    0.070  2
        HA    HB1    0.090  3
        CB    CT2   -0.180  4
       HB1    HA2    0.090  5
       HB2    HA2    0.090  6
        CG    CT2   -0.180  7
       HG1    HA2    0.090  8
       HG2    HA2    0.090  9
        CD    CT2   -0.180 10
       HD1    HA2    0.090 11
       HD2    HA2    0.090 12
        CE    CT2    0.071 13
       HE1    HA2    0.050 14
       HE2    HA2    0.050 15
        NZ  NG2S1   -0.566 16
        HZ     HC    0.314 17
        CH  CG2O1    0.405 18
        OH  OG2D1   -0.428 19
        CP CG2R61   -0.117 20
       CQ1 CG2R61   -0.114 21
       HQ1  HGR61    0.115 22
       CR1 CG2R61   -0.114 23
       HR1  HGR61    0.115 24
       CS2 CG2R61   -0.001 25
       CR2 CG2R61   -0.114 26
       HR2  HGR61    0.115 27
       CQ2 CG2R61   -0.114 28
       HQ2  HGR61    0.115 29
       CT2 CG2R67    0.219 30
       CU1  CG2O9    0.090 31
       CT1  CG2O9   -0.180 32
       CS1  CG331   -0.080 33
      HS11   HGA3    0.080 34
      HS12   HGA3    0.080 35
      HS13   HGA3    0.080 36
       CV1  CG2O8   -0.290 37
       HV1  HGR63    0.160 38
       CX1  CG2O9    0.100 39
       CY1  CG331   -0.080 40
      HY11   HGA3    0.080 41
      HY12   HGA3    0.080 42
      HY13   HGA3    0.080 43
       NW1  NG2D1   -0.200 44
       FY1   FGA2   -0.400 45
        BX     BL    0.799 46
       FY2   FGA2   -0.400 47
       NW2  NG2D1   -0.200 48
```

```
       CX2  CG2O9    0.100 49
       CY2  CG331   -0.080 50
      HY21   HGA3    0.080 51
      HY22   HGA3    0.080 52
      HY23   HGA3    0.080 53
       CV2  CG2O8   -0.290 54
       HV2  HGR63    0.160 55
       CT3  CG2O9   -0.180 56
       CS3  CG331   -0.080 57
      HS31   HGA3    0.080 58
      HS32   HGA3    0.080 59
      HS33   HGA3    0.080 60
       CU2  CG2O9    0.090 61
         C      C    0.510 62
         O      O   -0.510 63
[ bonds ]
        CB     CA
        CG     CB
        CD     CG
        CE     CD
        NZ     CE
         N     HN
         N     CA
         C     CA
         C     +N
        CA     HA
        CB    HB1
        CB    HB2
        CG    HG1
        CG    HG2
        CD    HD1
        CD    HD2
        CE    HE1
        CE    HE2
        NZ     HZ
        NZ     CH
        CH     OH
        CH     CP
        CP    CQ1
       CQ1    HQ1
       CQ1    CR1
       CR1    HR1
       CR1    CS2
       CS2    CT2
       CT2    CU1
       CU1    CT1
       CT1    CS1
       CS1   HS11
       CS1   HS12
       CS1   HS13
       CT1    CV1
       CV1    HV1
       CV1    CX1
       CX1    CY1
       CY1   HY11
       CY1   HY12
       CY1   HY13
       CX1    NW1
       NW1    CU1
       NW1     BX
        BX    FY1
```

```
       BX   FY2
       BX   NW2
      NW2   CU2
      NW2   CX2
      CX2   CY2
      CY2  HY21
      CY2  HY22
      CY2  HY23
      CX2   CV2
      CV2   HV2
      CV2   CT3
      CT3   CS3
      CS3  HS31
      CS3  HS32
      CS3  HS33
      CT3   CU2
      CU2   CT2
      CS2   CR2
      CR2   HR2
      CR2   CQ2
      CQ2   HQ2
      CQ2    CP
        O     C
  [ impropers ]
        N    -C    CA    HN
        C    CA    +N     O
  [ cmap ]
       -C     N    CA     C    +N

[ TRB ]
  [ atoms ]
        N    NH1   -0.470  0
       HN      H    0.310  1
       CA    CT1    0.070  2
       HA    HB1    0.090  3
       CB    CT2   -0.180  4
      HB1    HA2    0.090  5
      HB2    HA2    0.090  6
       CG     CY   -0.030  7
      CD1     CA   -0.150  8
      CQ1 CG2R61   -0.001  9
      CR1 CG2R61   -0.114 10
      HR1  HGR61    0.115 11
      CS2 CG2R61   -0.001 12
      CR2 CG2R61   -0.114 13
      HR2  HGR61    0.115 14
      CQ2 CG2R61   -0.114 15
      HQ1  HGR61    0.115 16
       CP CG2R61   -0.114 17
       HP  HGR61    0.115 18
      CT2 CG2R67    0.219 19
      CU1  CG2O9    0.090 20
      CT1  CG2O9   -0.180 21
      CS1  CG331   -0.080 22
     HS11   HGA3    0.080 23
     HS12   HGA3    0.080 24
     HS13   HGA3    0.080 25
      CV1  CG2O8   -0.290 26
      HV1  HGR63    0.160 27
      CX1  CG2O9    0.100 28
      CY1  CG331   -0.080 29
```

```
     HY11   HGA3    0.080 30
     HY12   HGA3    0.080 31
     HY13   HGA3    0.080 32
      NW1  NG2D1   -0.200 33
      FY1   FGA2   -0.400 34
       BX     BL    0.799 35
      FY2   FGA2   -0.400 36
      NW2  NG2D1   -0.200 37
      CX2  CG2O9    0.100 38
      CY2  CG331   -0.080 39
     HY21   HGA3    0.080 40
     HY22   HGA3    0.080 41
     HY23   HGA3    0.080 42
      CV2  CG2O8   -0.290 43
      HV2  HGR63    0.160 44
      CT3  CG2O9   -0.180 45
      CS3  CG331   -0.080 46
     HS31   HGA3    0.080 47
     HS32   HGA3    0.080 48
     HS33   HGA3    0.080 49
      CU2  CG2O9    0.090 50
      NE1     NY   -0.510 51
      HE1      H    0.370 52
      CE2    CPT    0.240 53
      CD2    CPT    0.110 54
      CE3    CAI   -0.250 55
      HE3     HP    0.170 56
      CZ3     CA   -0.200 57
      HZ3     HP    0.140 58
      CZ2    CAI   -0.270 59
      HZ2     HP    0.160 60
      CH2     CA   -0.140 61
      HH2     HP    0.140 62
        C      C    0.510 63
      O      O   -0.510 64
[ bonds ]
       CB    CA
       CG    CB
      CD2    CG
      NE1   CD1
      CZ2   CE2
        N    HN
        N    CA
        C    CA
        C    +N
      CZ3   CH2
      CD2   CE3
      NE1   CE2
       CA    HA
       CB   HB1
       CB   HB2
      CD1   CQ1
      CQ1   CR1
      CR1   HR1
      CR1   CS2
      CS2   CT2
      CT2   CU1
      CU1   CT1
      CT1   CS1
      CS1  HS11
      CS1  HS12
```

```
        CS1  HS13
        CT1   CV1
        CV1   HV1
        CV1   CX1
        CX1   CY1
        CY1  HY11
        CY1  HY12
        CY1  HY13
        CX1   NW1
        NW1   CU1
        NW1    BX
         BX   FY1
         BX   FY2
         BX   NW2
        NW2   CU2
        NW2   CX2
        CX2   CY2
        CY2  HY21
        CY2  HY22
        CY2  HY23
        CX2   CV2
        CV2   HV2
        CV2   CT3
        CT3   CS3
        CS3  HS31
        CS3  HS32
        CS3  HS33
        CT3   CU2
        CU2   CT2
        CS2   CR2
        CR2   HR2
        CR2   CQ2
        CQ2   HQ1
        CQ2    CP
        CP     HP
        CP    CQ1
        NE1   HE1
        CE3   HE3
        CZ2   HZ2
        CZ3   HZ3
        CH2   HH2
          O     C
        CD1    CG
        CE2   CD2
        CZ3   CE3
        CH2   CZ2
  [ impropers ]
         N    -C    CA    HN
         C    CA    +N     O
  [ cmap ]
        -C     N    CA     C    +N
```

Hydrogen atoms added to merged.hdb

```
CYB         13
1     1     HN    N     -C    CA
1     5     HA    CA    N     C     CB
2     6     HB    CB    SG    CA
1     1     HR1 CR1 CQ1 CS2
1     1     HR2 CR2 CS2 CQ2
1     1     HQ1 CQ1  CP CR1
1     1     HQ2 CQ2 CR2  CP
```

```
3     4     HS1   CS1   CT1   CU1
1     1     HV1 CV1 CT1 CX1
3     4     HY1   CY1   CX1   CV1
3     4     HY2   CY2   CX2   NW2
1     1     HV2 CV2 CY2 CT3
3     4     HS3   CS3   CT3   CV2

DPB         14
1     1     HN    N     -C    CA
1     5     HA    CA    N     C     CB
2     6     HB    CB    NG    CA
1     4     HG    NG    CD    CB
1     1     HR1 CR1 CQ1 CS2
1     1     HR2 CR2 CS2 CQ2
1     1     HQ1 CQ1  CP CR1
1     1     HQ2 CQ2 CR2  CP
3     4     HS1   CS1   CT1   CU1
1     1     HV1 CV1 CT1 CX1
3     4     HY1   CY1   CX1   CV1
3     4     HY2   CY2   CX2   NW2
1     1     HV2 CV2 CY2 CT3
3     4     HS3   CS3   CT3   CV2

HCB         14
1     1     HN    N     -C    CA
1     5     HA    CA    N     C     CB
2     6     HB    CB    CG    CA
2     6     HG    CG    SD    CB
1     1     HR1 CR1 CQ1 CS2
1     1     HR2 CR2 CS2 CQ2
1     1     HQ1 CQ1  CP CR1
1     1     HQ2 CQ2 CR2  CP
3     4     HS1   CS1   CT1   CU1
1     1     HV1 CV1 CT1 CX1
3     4     HY1   CY1   CX1   CV1
3     4     HY2   CY2   CX2   NW2
1     1     HV2 CV2 CY2 CT3
3     4     HS3   CS3   CT3   CV2

LYB         17
1     1     HN    N     -C    CA
1     5     HA    CA    N     C     CB
2     6     HB    CB    CG    CA
2     6     HG    CG    CD    CB
2     6     HD    CD    CE    CG
2     6     HE    CE    NZ    CD
1     4     HZ    NZ    CE    CD
1     1     HR1 CR1 CQ1 CS2
1     1     HR2 CR2 CS2 CQ2
1     1     HQ1 CQ1  CP CR1
1     1     HQ2 CQ2 CR2  CP
3     4     HS1   CS1   CT1   CU1
1     1     HV1 CV1 CT1 CX1
3     4     HY1   CY1   CX1   CV1
3     4     HY2   CY2   CX2   NW2
1     1     HV2 CV2 CY2 CT3
3     4     HS3   CS3   CT3   CV2

TRB         18
1     1     HN    N     -C    CA
1     5     HA    CA    N     C     CB
```

```
2     6     HB    CB    CG    CA
1     1     HE1 NE1 CD1 CE2
1     1     HE3 CE3 CD2 CZ3
1     1     HZ3 CZ3 CE3 CH2
1     1     HH2 CH2 CZ3 CZ2
1     1     HZ2 CZ2 CE2 CH2
1     1     HR1 CR1 CQ1 CS2
1     1     HR2 CR2 CS2 CQ2
1     1     HQ1 CQ2 CR2  CP
1     1     HP  CP  CQ2 CQ1
3     4     HS1   CS1   CT1   CU1
1     1     HV1 CV1 CT1 CX1
3     4     HY1   CY1   CX1   CV1
3     4     HY2   CY2   CX2   NW2
1     1     HV2 CV2 CY2 CT3
3     4     HS3   CS3   CT3   CV2
```

Atom types added to ffnonbonded.itp

```
[ atomtypes ]
;type atnum          mass   charge ptype         sigma  epsilon
   BL      5    10.810000    0.000  A  0.356359487256  1.88280
CG2O8      6    12.011000    0.000  A  0.320723538531  0.20920
CG2O9      6    12.011000    0.000  A  0.320723538531  0.37656
```

Bond types, angles and dihedrals added to ffbonded.itp

```
[ bondtypes ]
;for Trp(BODIPY)
;      i        j  func           b0           kb
      BL     FGA2     1   0.13750000    258767.31
      BL    NG2D1     1   0.15682000    303579.00
   CG2O8    NG2D1     1   0.13800000    334720.00
   CG2O8    HGR63     1   0.10900000    284512.00
   CG2O8    CG2O8     1   0.13600000    343088.00
   CG2O8    CG2O9     1   0.14000000    301248.00
   NG2D1    CG2O9     1   0.13855000    225936.00
   CG2O9   CG2R67     1   0.13680000    255224.00
   CG2O9    CG2O9     1   0.13600000    343088.00
      CA   CG2R61     1   0.14400000    251040.00
   CG2O9    CG331     1   0.15000000    192154.40
;for Cys(BODIPY)
   SG311   CG2R61     1   0.17500000    234304.00
     CT2    SG311     1   0.18180000    165686.40
;for Lys(BODIPY)
     NH2    CG2O1     1   0.13450000    309616.00
[ angletypes ]
;for Trp(BODIPY)
;      i        j        k  func      theta0      ktheta         ub0          kub
   CG2O8   NG2D1    CG2O9     5   110.000000  1087.840000  0.00000000         0.00
  CG2R61  CG2R61   HGR61     5  120.000000   251.040000  0.21525000     18409.60
  CG2R67  CG2R61   HGR61     5   120.000000   251.040000  0.00000000         0.00
  CG2R61  CG2R67  CG2R67     5   120.000000   334.720000  0.00000000         0.00
  CG2R61  CG2R67  CG2R61     5   120.000000   334.720000  0.00000000         0.00
  CG2R61  CG2R61  CG2R67     5   120.000000   334.720000  0.00000000         0.00
  CG2R61  CG2R61  CG2R61     5  120.000000   334.720000  0.24162000     29288.00
  CG2R61  CG2R61   OG301     5   120.000000   920.480000  0.00000000         0.00
  CG2R61   OG301   CG321     5   108.000000   543.920000  0.00000000         0.00
   OG301   CG321    HGA2     5   108.890000   384.091200  0.00000000         0.00
   CG321   CG321   OG301     5   111.500000   376.560000  0.00000000         0.00
    FGA2      BL    FGA2     5  113.600000   396.493413  0.25610000     30848.46
    FGA2      BL   NG2D1     5  113.600000   263.692416  0.25610000      4466.90
   NG2D1      BL   NG2D1     5  106.000000   784.104194  0.24162000      6451.19
```

```
        BL    NG2D1    CG2O8     5   120.000000   123.687408   0.24162000      4202.13
        BL    NG2D1    CG2O9     5   120.000000   123.687408   0.24162000      4202.13
     NG2D1    CG2O9   CG2R67     5   120.000000  1338.880000   0.00000000         0.00
     NG2D1    CG2O9    CG2O8     5   120.000000  1338.880000   0.00000000         0.00
     NG2D1    CG2O8    CG2O8     5   110.000000  1087.840000   0.00000000         0.00
     CG2O8    CG2O8    CG2O8     5   107.400000   920.480000   0.00000000         0.00
     CG2O8    CG2O8    HGR63     5   126.400000   267.776000   0.22550000     20920.00
     CG2O8    CG2O8    CG2O9     5   107.400000   920.480000   0.00000000         0.00
     CG2O8    CG2O9   CG2R67     5   130.600000  1338.880000   0.00000000         0.00
     CG2O9   CG2R67    CG2O9     5   122.000000  1338.880000   0.00000000         0.00
     CG2O9    NG2D1    CG2O9     5   110.000000  1087.840000   0.00000000         0.00
     NG2D1    CG2O8    HGR63     5   123.000000   317.984000   0.00000000         0.00
     CG2O9    CG2O8    HGR63     5   126.400000   267.776000   0.22550000     20920.00
     CG2O9   CG2R67   CG2R67     5   120.000000   334.720000   0.00000000         0.00
    CG2R67    CG2O9    CG2O9     5   130.000000   292.880000   0.00000000         0.00
     CG2O8    CG2O9    CG2O9     5   107.200000   753.120000   0.00000000         0.00
     CG2O9    CG2O8    CG2O9     5   107.200000   753.120000   0.00000000         0.00
     CG2O9    CG2O9    CG2O8     5   107.200000   753.120000   0.00000000         0.00
        CY       CA   CG2R61     5   107.200000   753.120000   0.00000000         0.00
     CG2O8    CG2O9    CG331     5   130.000000   383.254400   0.00000000         0.00
     CG2O9    CG2O9    CG331     5   130.000000   383.254400   0.00000000         0.00
     CG2O9    CG2O9    NG2D1     5   106.000000  1087.840000   0.00000000         0.00
        CA   CG2R61   CG2R61     5   120.000000   267.776000   0.00000000         0.00
     CG2O9    CG331     HGA3     5   109.500000   460.240000   0.00000000         0.00
    CG2R61   CG2R67    CG2O9     5   120.000000   334.720000   0.00000000         0.00
    CG2R61       CA       NY     5   124.000000   292.880000   0.00000000         0.00
     CG331    CG2O9    CG2O8     5   130.000000   383.254400   0.00000000         0.00
     CG331    CG2O9    NG2D1     5   124.000000   383.254400   0.00000000         0.00
     NG2D1    CG2O9    CG331     5   124.000000   383.254400   0.00000000         0.00
;for Cys(BODIPY)
       CT2    SG311   CG2R61     5    95.000000   284.512000   0.00000000         0.00
     SG311   CG2R61   CG2R61     5   120.000000   334.720000   0.00000000         0.00
       CT1      CT2    SG311     5   112.500000   485.344000   0.00000000         0.00
       HA2      CT2    SG311     5   111.300000   385.764800   0.00000000         0.00
;for Lys(BODIPY)
       CT2      NH2    CG2O1     5   120.000000   418.400000   0.00000000         0.00
        HC      NH2    CG2O1     5   123.000000   284.512000   0.00000000         0.00
       NH2    CG2O1   OG2D1     5   122.500000   669.440000   0.00000000         0.00
       NH2    CG2O1   CG2R61     5   116.500000   669.440000   0.00000000         0.00
;for hCys(BODIPY)
       CT2      CT2    SG311     5   114.500000   485.344000   0.00000000         0.00
;for Dap(BODIPY)
       CT1      CT2      NH2     5   113.500000   585.760000   0.00000000         0.00
[ dihedraltypes ]
;for Trp(BODIPY)
;       i         j          k          l   func         phi0          kphi   mult
      FGA2        BL     NG2D1      CG2O9      9   180.000000      6.323823      4
        BL     NG2D1     CG2O9      CG2O8      9   180.000000      4.189816      2
        BL     NG2D1     CG2O9      CG331      9   180.000000      4.189816      2
        BL     NG2D1     CG2O9     CG2R67      9   180.000000      4.189816      2
     NG2D1     CG2O9     CG331       HGA3      9   180.000000     12.552000      3
     NG2D1     CG2O9     CG2O8      HGR63      9   180.000000     12.552000      1
     NG2D1     CG2O9     CG2O9      CG2O8      9   180.000000     58.576000      1
     NG2D1     CG2O9     CG2O8      CG2O9      9   180.000000     58.576000      1
     NG2D1     CG2O9    CG2R67      CG2O9      9   180.000000     58.576000      1
     NG2D1     CG2O9    CG2R67     CG2R61      9   180.000000      6.694400      1
     CG2O9     CG2O8     CG2O9      CG331      9   180.000000     14.644000      1
     CG2O9     CG2O8     CG2O9      CG2O9      9   180.000000     12.970400      1
     CG2O9     CG2O9     CG331       HGA3      9   180.000000     14.644000      3
     CG2O9     CG2O9     CG2O8      CG2O9      9   180.000000     12.970400      1
     CG2O9     CG2O9     CG2O8      HGR63      9   180.000000     14.644000      1
```

```
   CG2O9   CG2R67   CG2R61   CG2R61       9   180.000000      3.723760      2
   CG2O9   CG2R67    CG2O9    CG2O9       9   180.000000     12.970400      1
   CG2O8    CG2O9    CG331     HGA3       9   180.000000     14.644000      3
   CG2O8    CG2O9    CG2O9   CG2R67       9   180.000000     12.970400      1
   HGR63    CG2O8    CG2O9    CG331       9   180.000000     14.644000      1
   CG331    CG2O9    CG2O9   CG2R67       9   180.000000     12.970400      1
  CG2R67    CG2O9    NG2D1    CG2O9       9   180.000000     58.576000      2
      CY       CA   CG2R61   CG2R61       9   180.000000     12.970400      2
   CG2O8    CG2O9    NG2D1    CG2O9       9   180.000000     41.840000      2
   CG2O9    CG2O9    NG2D1    CG2O9       9   180.000000     41.840000      2
      CA   CG2R61   CG2R61   CG2R61       9   180.000000     12.970400      2
      CA   CG2R61   CG2R61    HGR61       9   180.000000     10.041600      2
  CG2R61       CA       NY      CPT       9   180.000000     25.104000      2
  CG2R61       CA       NY        H       9   180.000000      4.184000      2
     CPT       CY       CA   CG2R61       9   180.000000      0.000000      2
     CT2       CY       CA   CG2R61       9   180.000000      0.000000      2
      NY       CA   CG2R61   CG2R61       9   180.000000      3.723760      2
   CG2O9    CG2O9    NG2D1       BL       9   180.000000      4.189816      2
   CG2O9    NG2D1    CG2O9   CG2R67       9   180.000000     58.576000      2
   CG2O9    NG2D1    CG2O9    CG331       9   180.000000     12.552000      2
   CG2O9    NG2D1       BL    NG2D1       9   180.000000     25.942010      2
  CG2R61   CG2R67    CG2O9    CG2O9       9   180.000000     12.970400      2
      BL    NG2D1    CG2O9    CG2O9       9   180.000000      4.189816      2
   CG331    CG2O9    CG2O9    NG2D1       9   180.000000     12.552000      2
   CG331    CG2O9    NG2D1    CG2O9       9   180.000000     12.552000      2
   NG2D1    CG2O9    CG2O9    CG331       9   180.000000     12.552000      2
   NG2D1       BL    NG2D1    CG2O9       9   180.000000     25.942010      2
;for Cys(BODIPY)
     CT1      CT2    SG311   CG2R61       9   180.000000      1.004160      1
     HA2      CT2    SG311   CG2R61       9     0.000000      1.171520      2
     CT2    SG311   CG2R61   CG2R61       9     0.000000      7.322000      2
   SG311   CG2R61   CG2R61    HGR61       9   180.000000     10.041600      2
   SG311   CG2R61   CG2R61   CG2R61       9   180.000000     18.828000      2
;for Lys(BODIPY)
     CT2      CT2      NH2    CG2O1       9     0.000000      7.531200      1
     HA2      CT2      NH2    CG2O1       9     0.000000      0.000000      3
     CT2      NH2    CG2O1    OG2D1       9   180.000000     10.460000      2
     CT2      NH2    CG2O1   CG2R61       9   180.000000     10.460000      2
      HC      NH2    CG2O1    OG2D1       9   180.000000     10.460000      2
      HC      NH2    CG2O1   CG2R61       9   180.000000     10.460000      2
     NH2    CG2O1   CG2R61   CG2R61       9   180.000000      4.184000      2
; for hCys(BODIPY)
     CT2      CT2    SG311   CG2R61       9   180.000000      1.004160      1
; for Dap(BODIPY)
     CT1      CT2      NH2       HC       9     0.000000      0.000000      1
     CT1      CT2      NH2    CG2O1       9     0.000000      7.531200      1
```

Supporting figures

Figures S1-32: General analysis of peptide-membrane systems. **a)** Minimum distance of the peptide to the membrane throughout the trajectory. **b)** Histogram of the distances of the peptide to the membrane shown as probability. **c)** In grey the positions of the phosphor atoms of the lipid head groups in the upper and lower leaflet, in red the carbon atom of the peptide's C-terminus and in green the nitrogen atom of the peptide's N-terminus showing their position in the z-dimension throughout the trajectory. **d)** the fraction of the peptide in contact with the membrane throughout the trajectory. **e)** Minimum distance of the tryptophan (Figures S1-S8) or BODIPY-labelled (Figures S9-S32) residue to the membrane throughout the trajectory. **f)** Histogram of the distances of the tryptophan (Figures S1-S8) or BODIPY-labelled (Figures S9-S32) residue to the membrane shown as probability. **g)** In grey the positions of the phosphor atoms of the lipid head groups in the upper and lower leaflet, for Figures S1-S8 in orange the position nitrogen atom in the indole of tryptophan and for Figures S9-S32 in magenta the position of the boron atom in the BODIPY showing their position in the z-dimension throughout the trajectory. **h)** Root Mean Square Distance (RMSD) of the peptide as determined by MDAnalysis throughout the trajectory. **i)** Raduis of gyration (Rgyr) of the peptide as determined by MDAnalysis throughout the trajectory. **j)** Surface accessible solvent ares (SASA) of the peptide as determined by Gromacs throughout the trajectory. **k)** Ramachandran plot of the peptide. **l)** Ramachandran plot of the tryptophan (Figures S1-S8) or BODIPY-labelled (Figures S9-S32) residue.

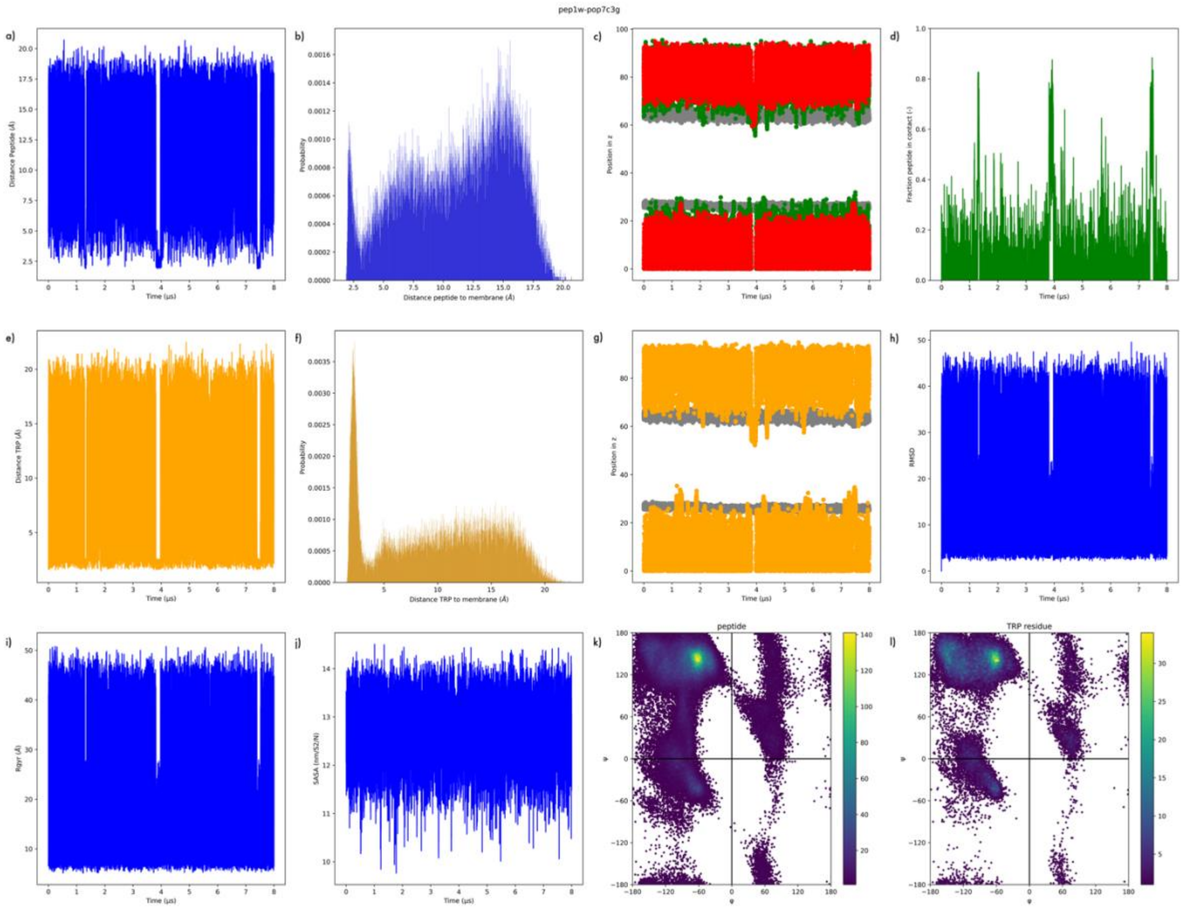

Figure S1 peptide 1 with tryptophan residue in POPC/POPG membrane

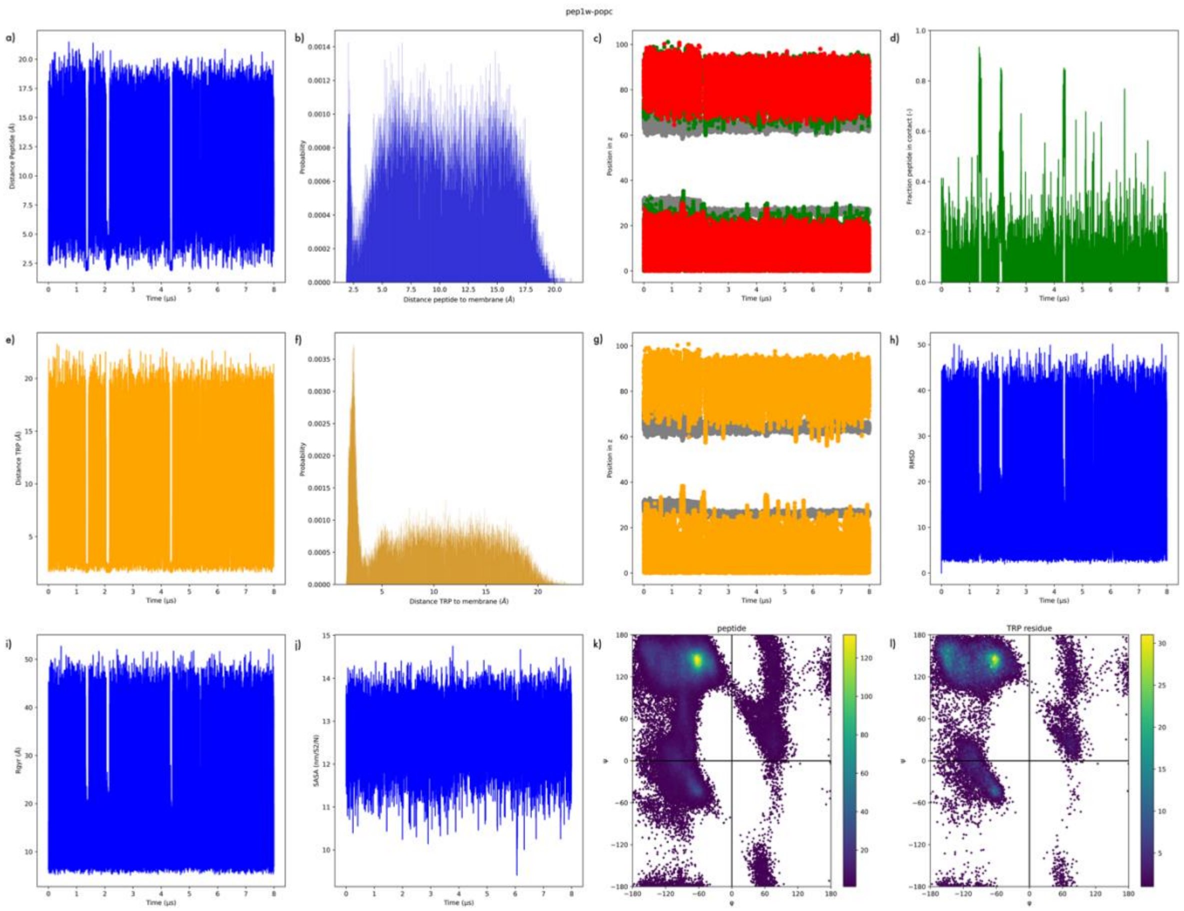

Figure S2 peptide 1 with tryptophan residue in POPC membrane

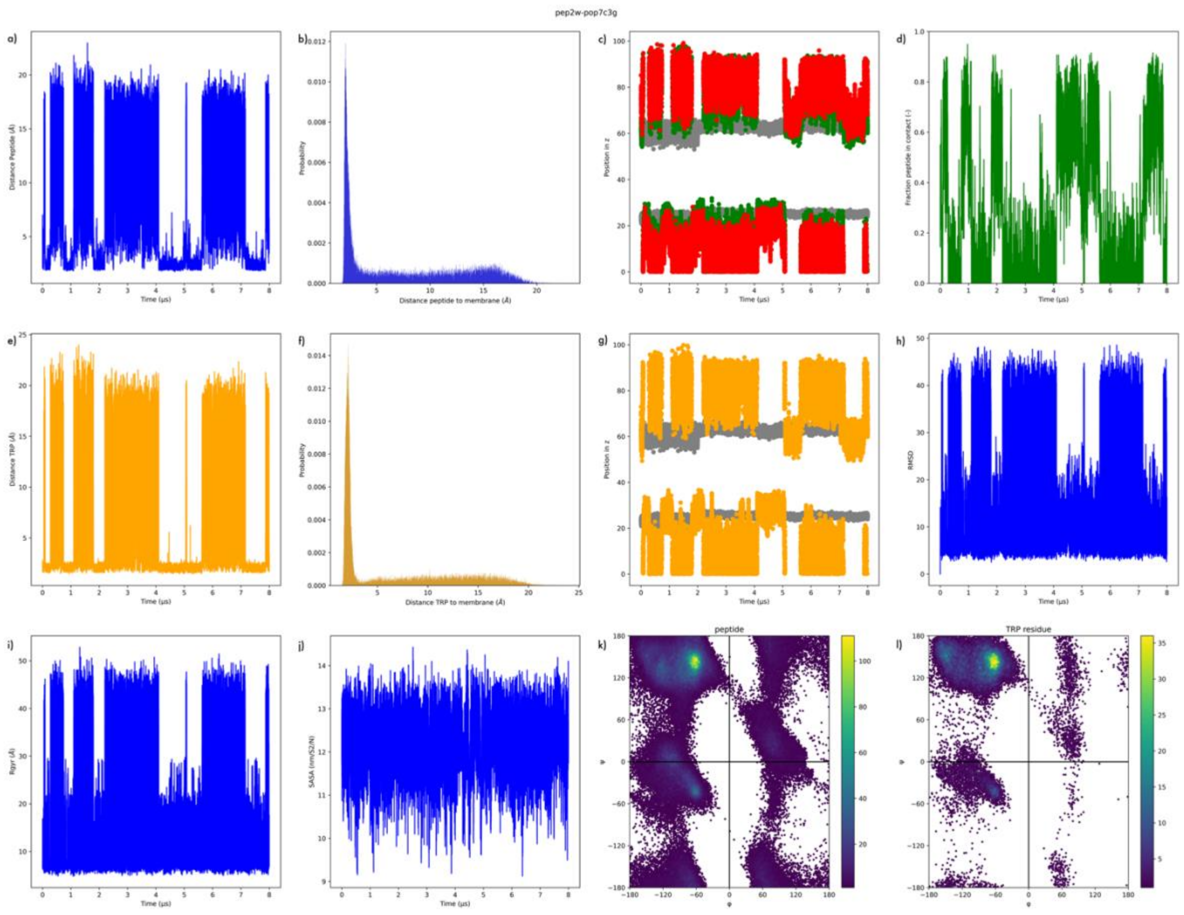

Figure S3 peptide 2 with tryptophan residue in POPC/POPG membrane

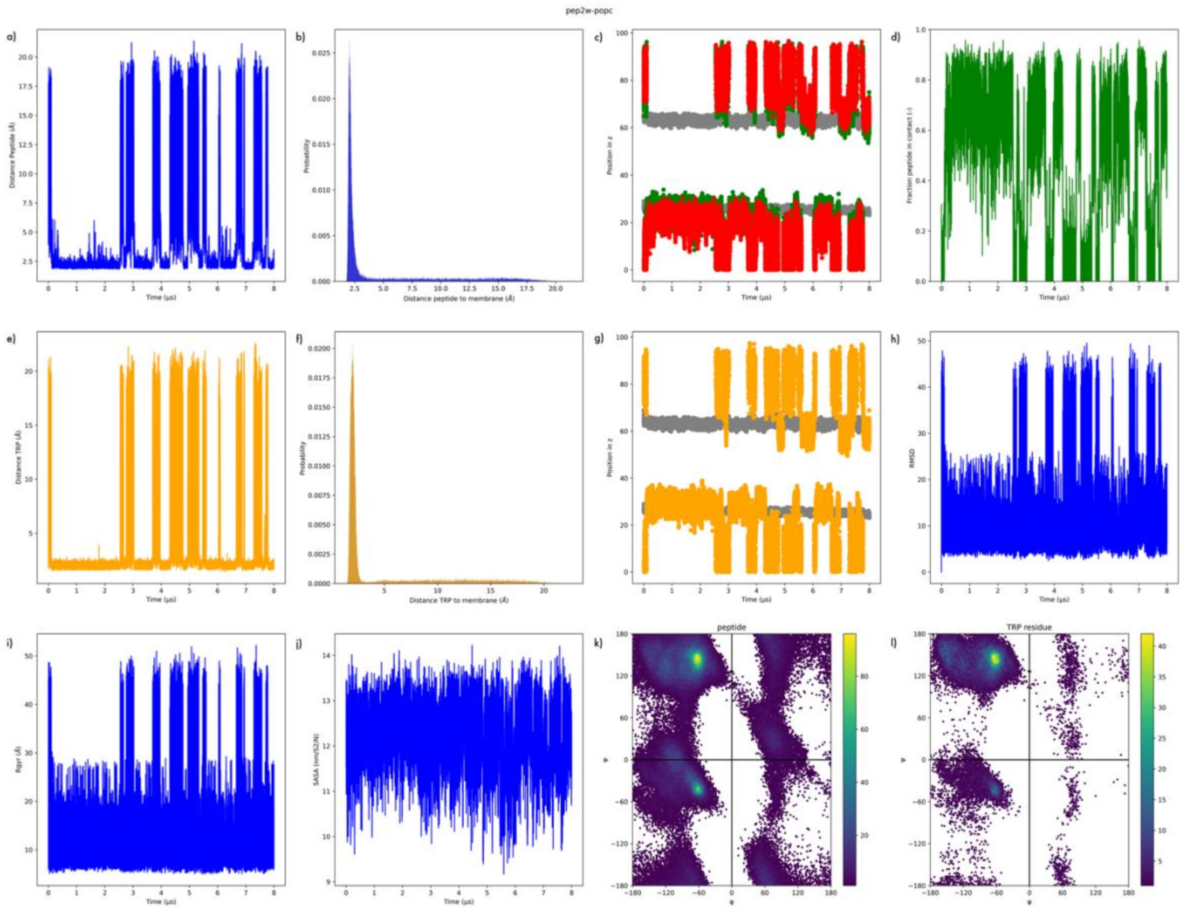

Figure S4 peptide 2 with tryptophan residue in POPC membrane

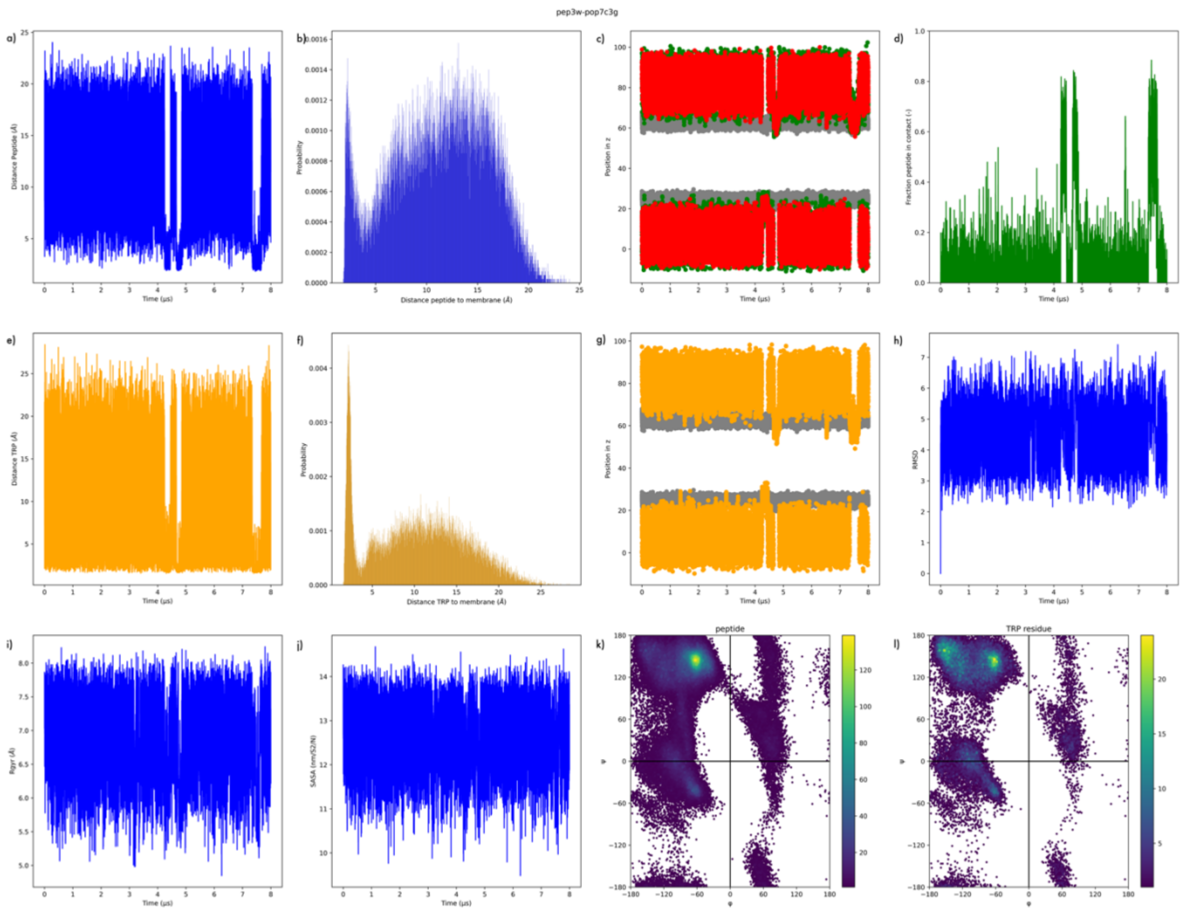

Figure S5 peptide 3 with tryptophan residue in POPC/POPG membrane

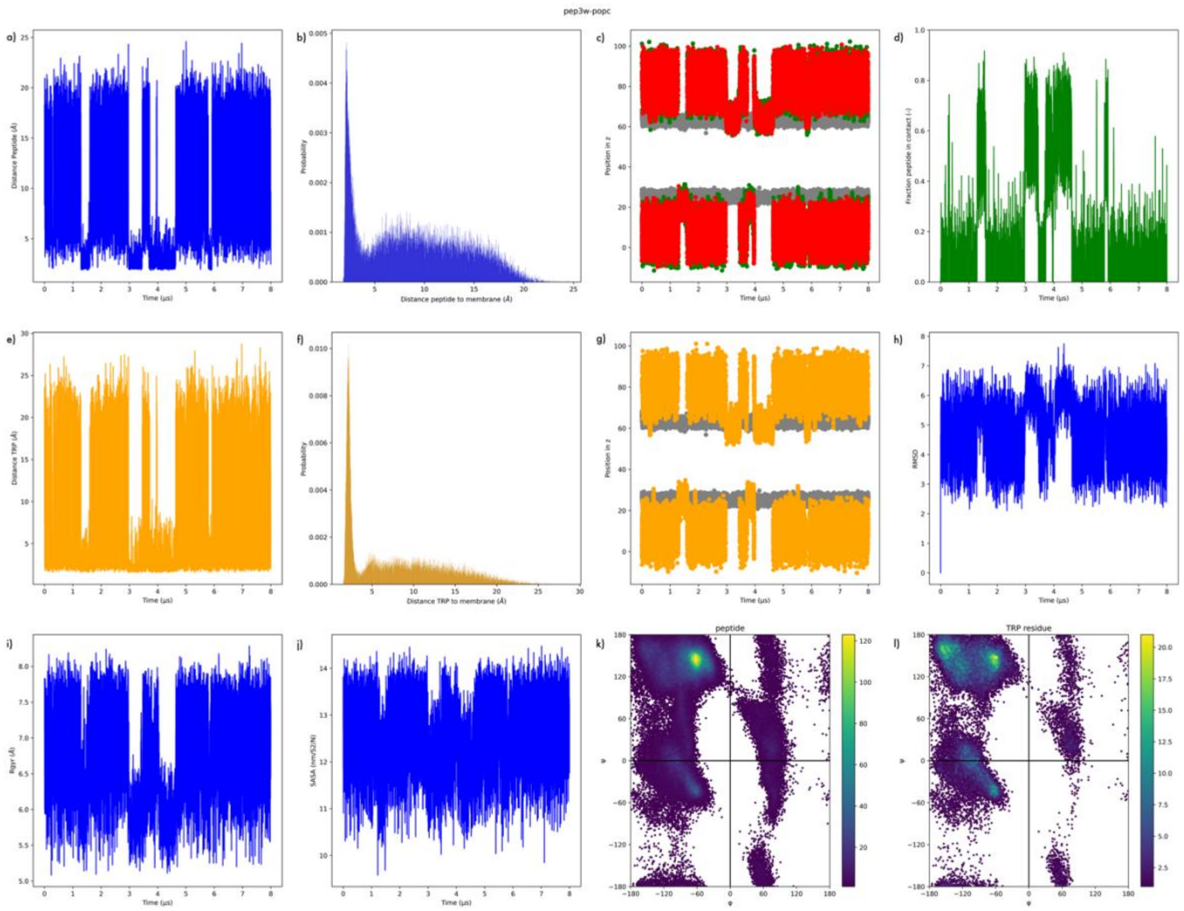

Figure S6 peptide 3 with tryptophan residue in POPC membrane

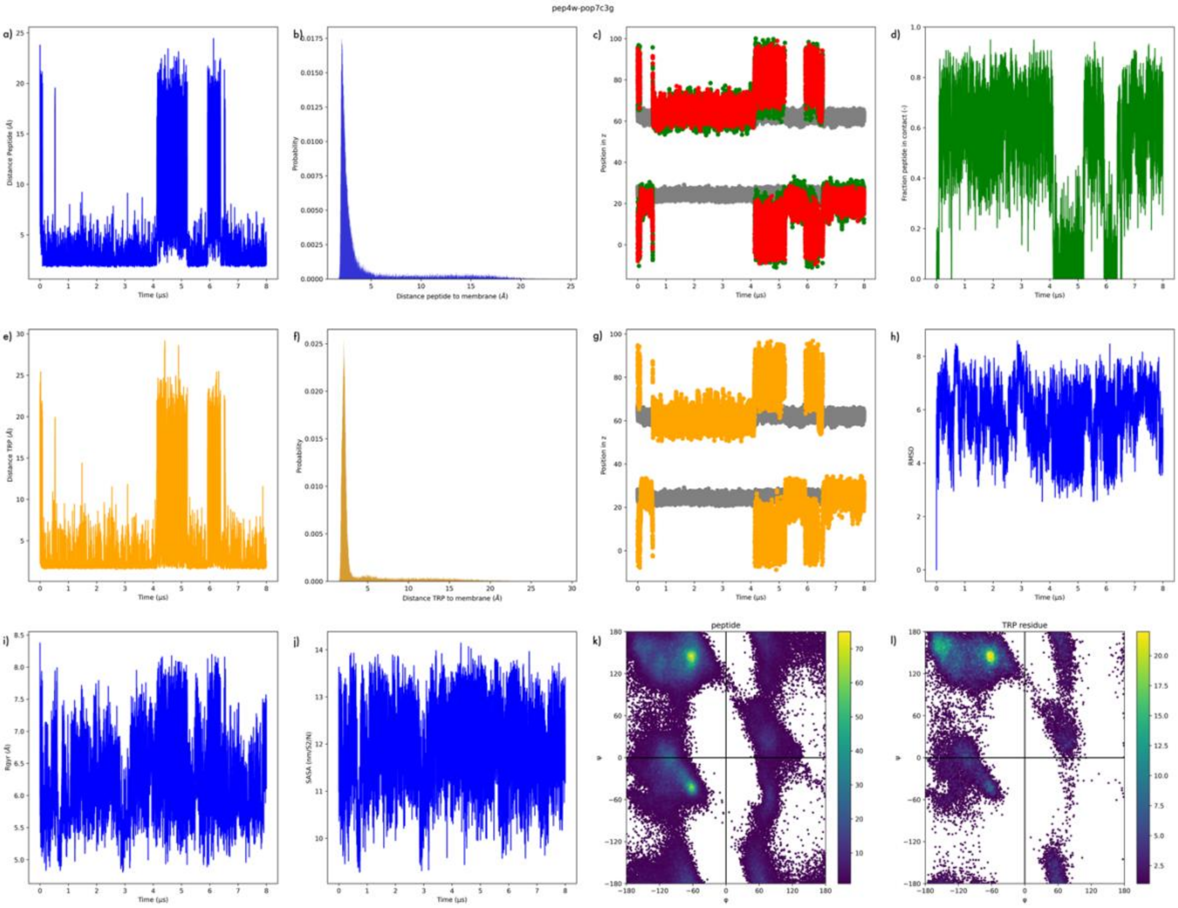

Figure S7 peptide 4 with tryptophan residue in POPC/POPG membrane

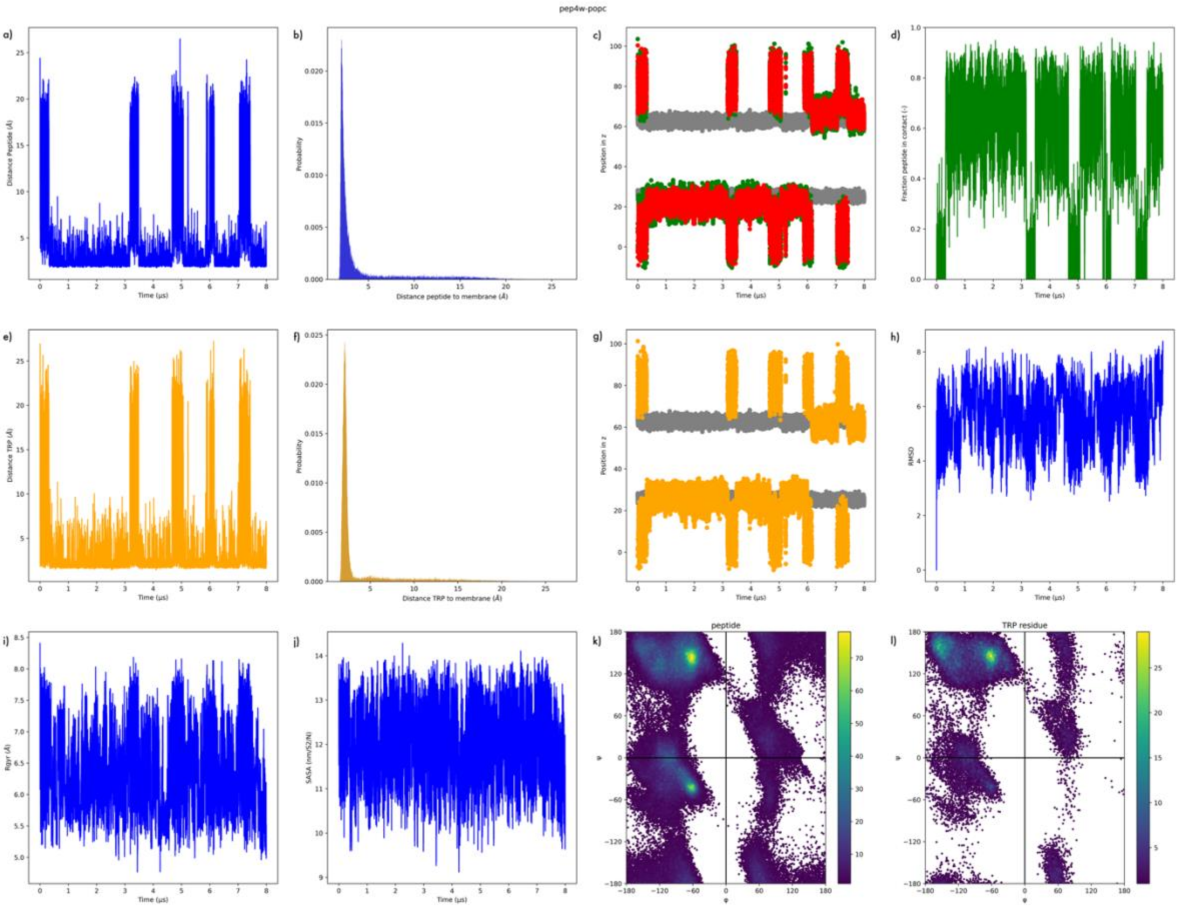

Figure S8 peptide 4 with tryptophan residue in POPC membrane

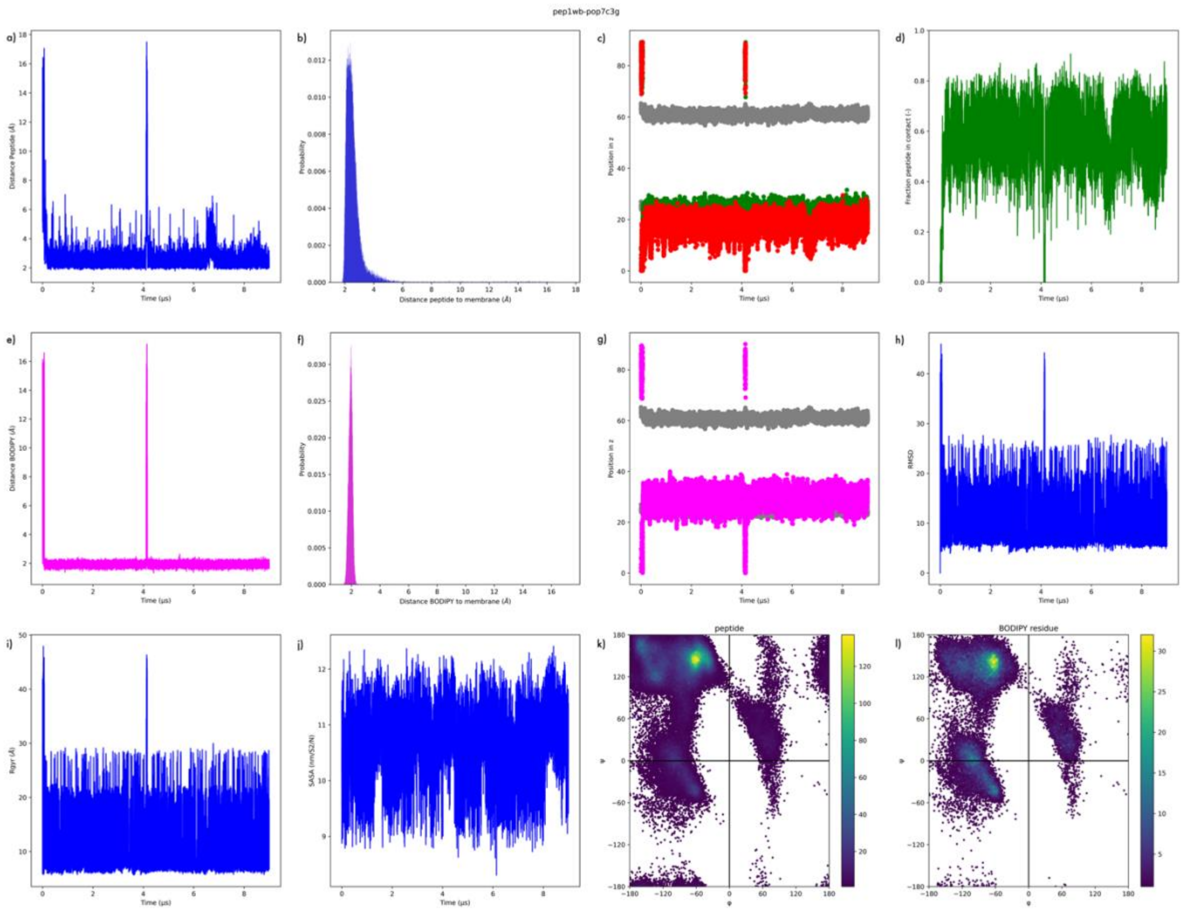

Figure S9 peptide 1 with BODIPY labelled tryptophan residue in POPC/POPG membrane

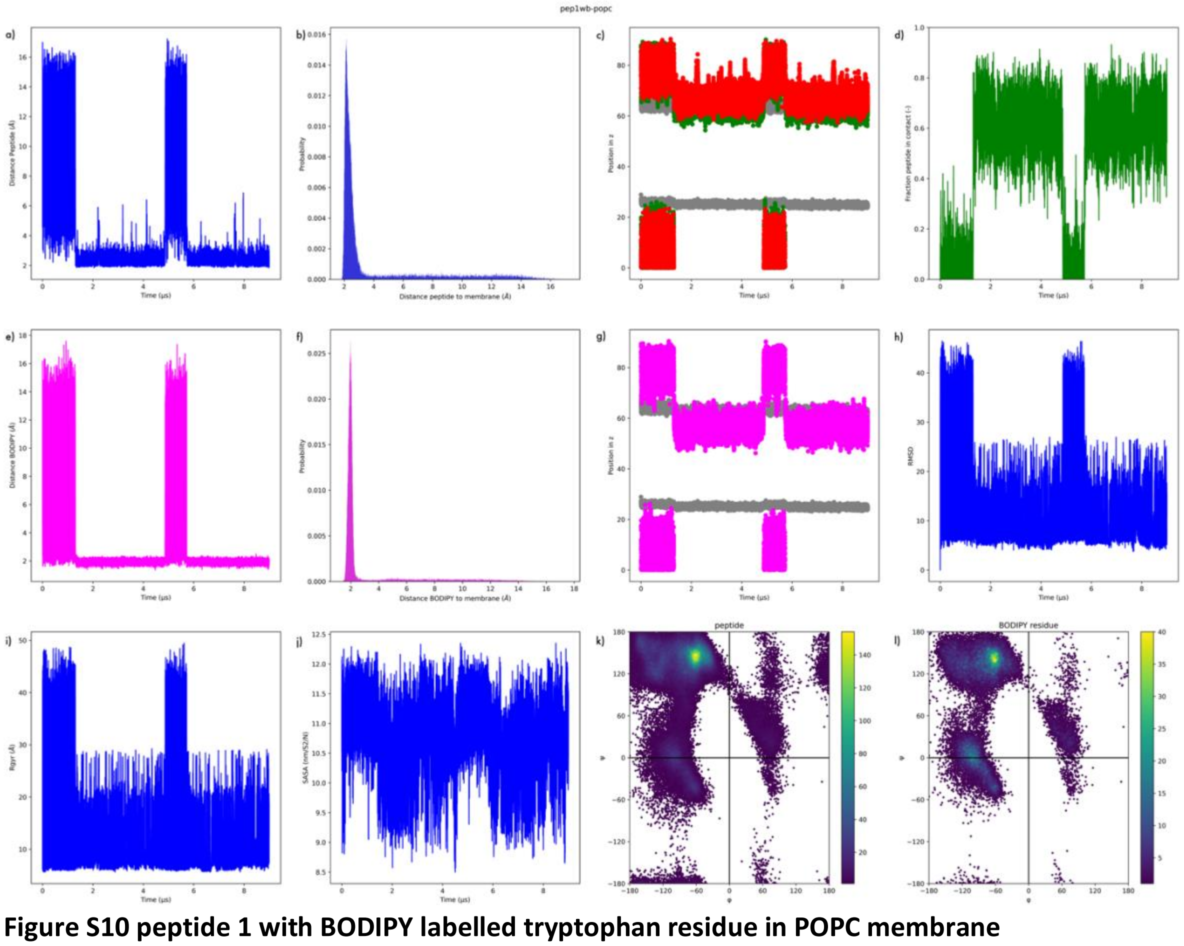

Figure S10 peptide 1 with BODIPY labelled tryptophan residue in POPC membrane

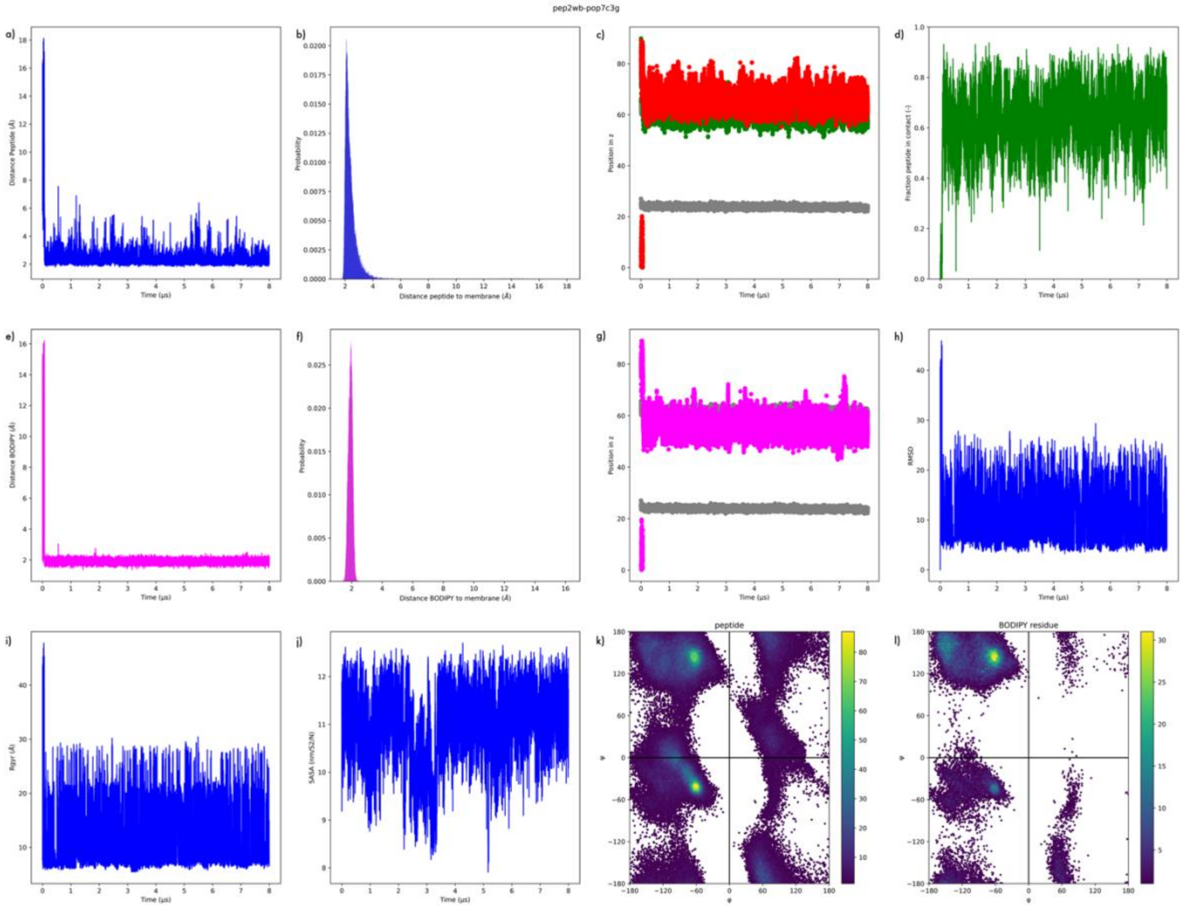

Figure S11 peptide 2 with BODIPY labelled tryptophan residue in POPC/POPG membrane

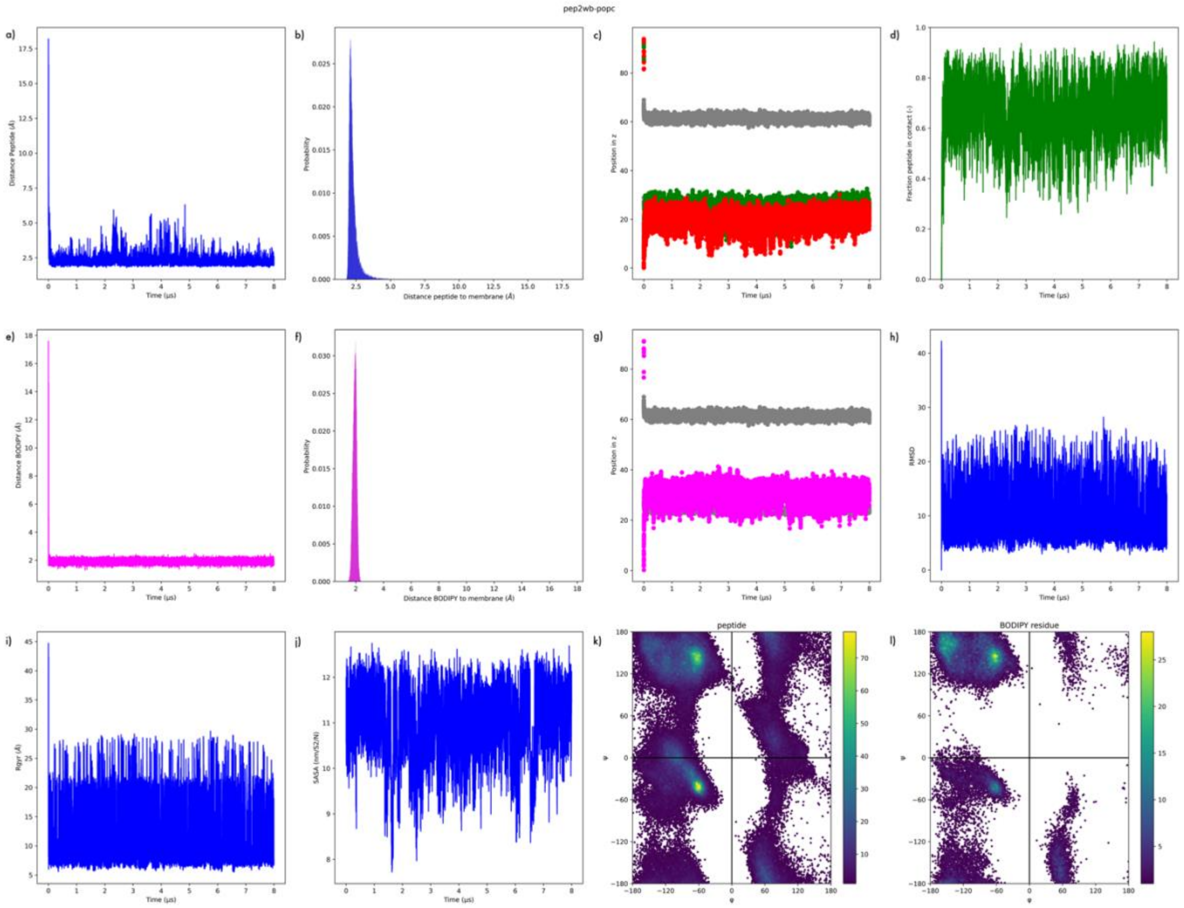

Figure S12 peptide 2 with BODIPY labelled tryptophan residue in POPC membrane

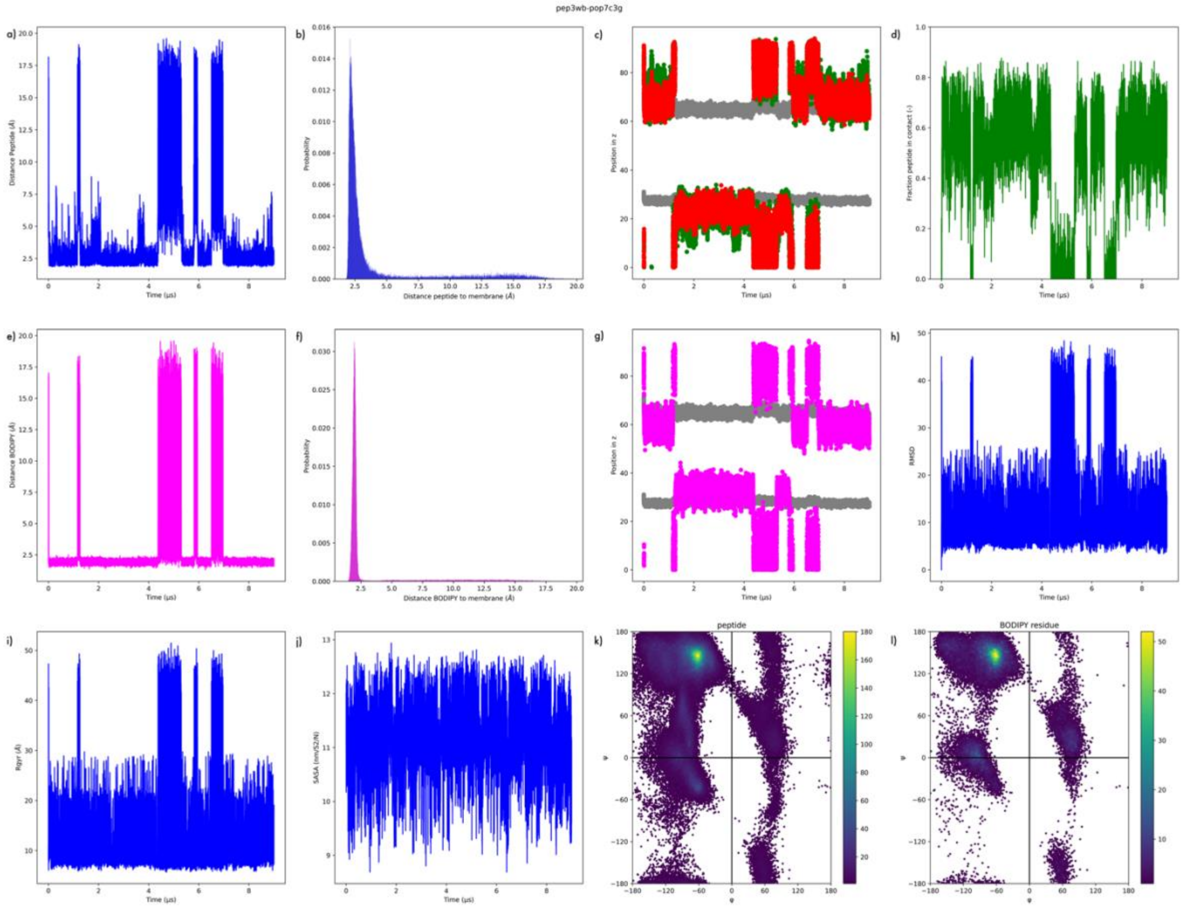

Figure S13 peptide 3 with BODIPY labelled tryptophan residue in POPC/POPG membrane

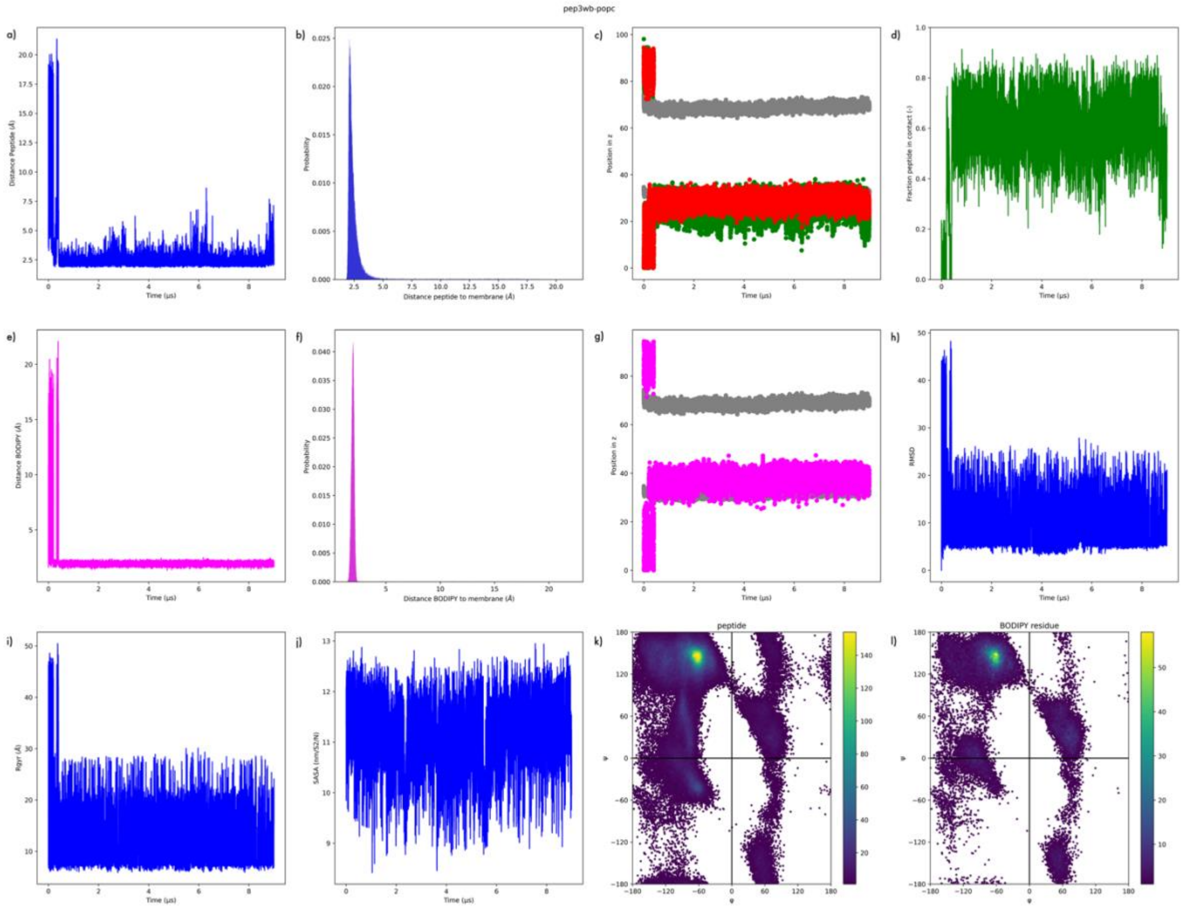

Figure S14 peptide 3 with BODIPY labelled tryptophan residue in POPC membrane

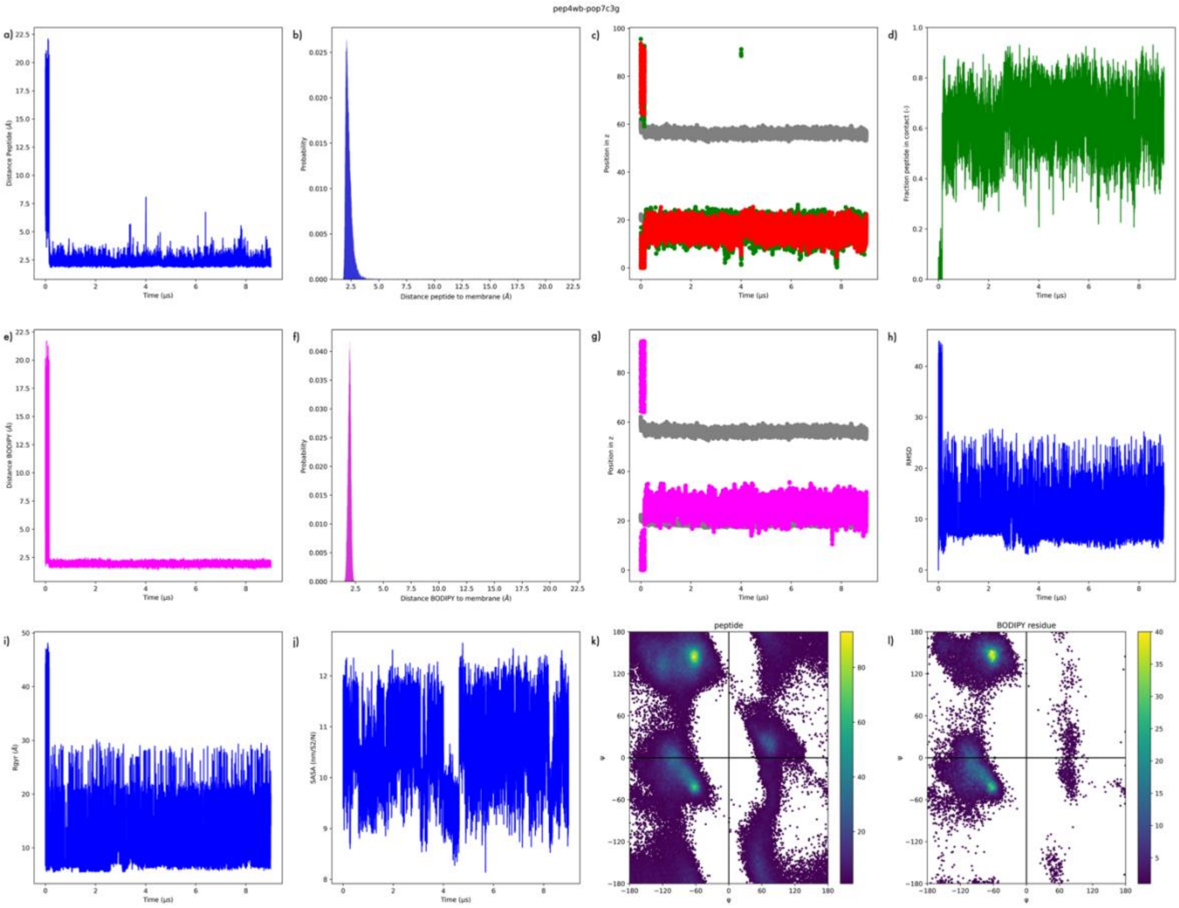

Figure S15 peptide 4 with BODIPY labelled tryptophan residue in POPC/POPG membrane

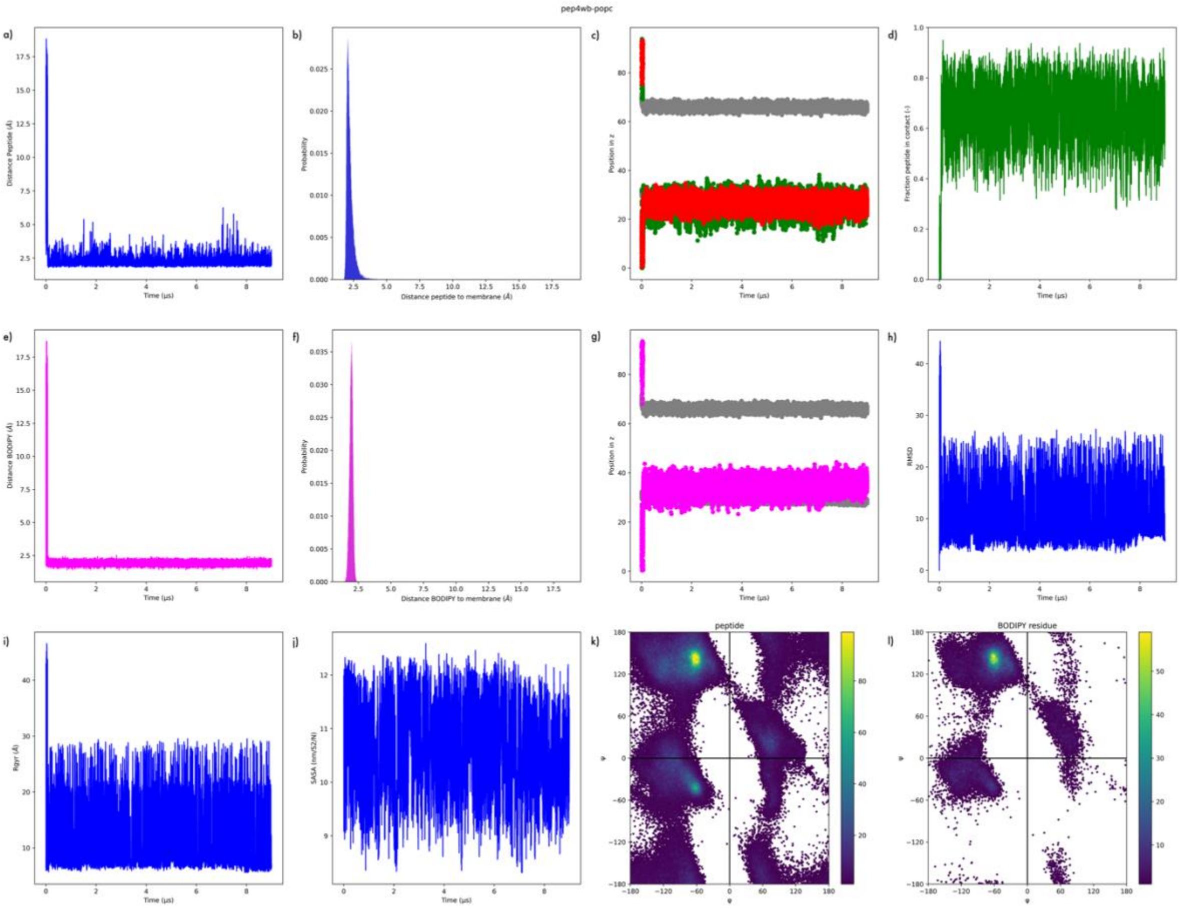

Figure S16 peptide 4 with BODIPY labelled tryptophan residue in POPC membrane

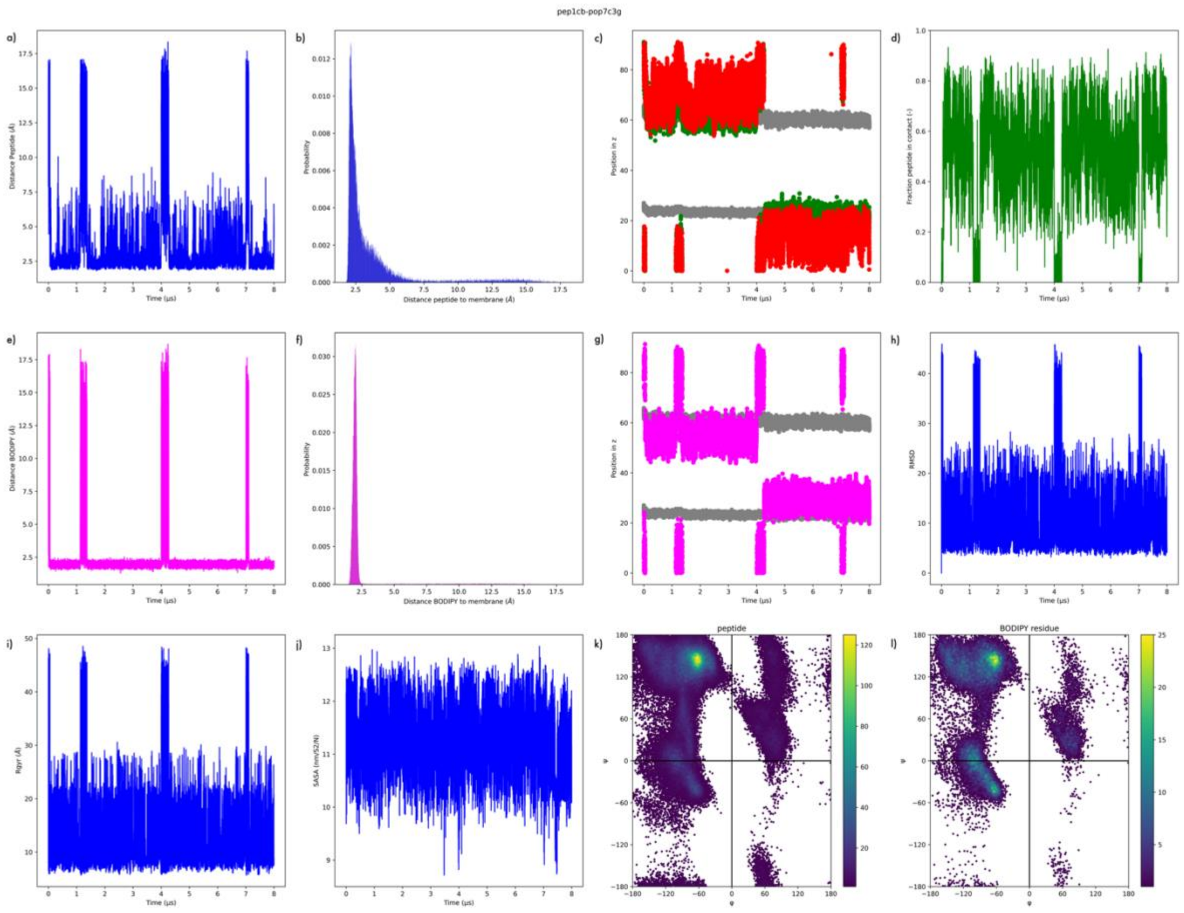

Figure S17 peptide 1 with BODIPY labelled cysteine residue in POPC/POPG membrane

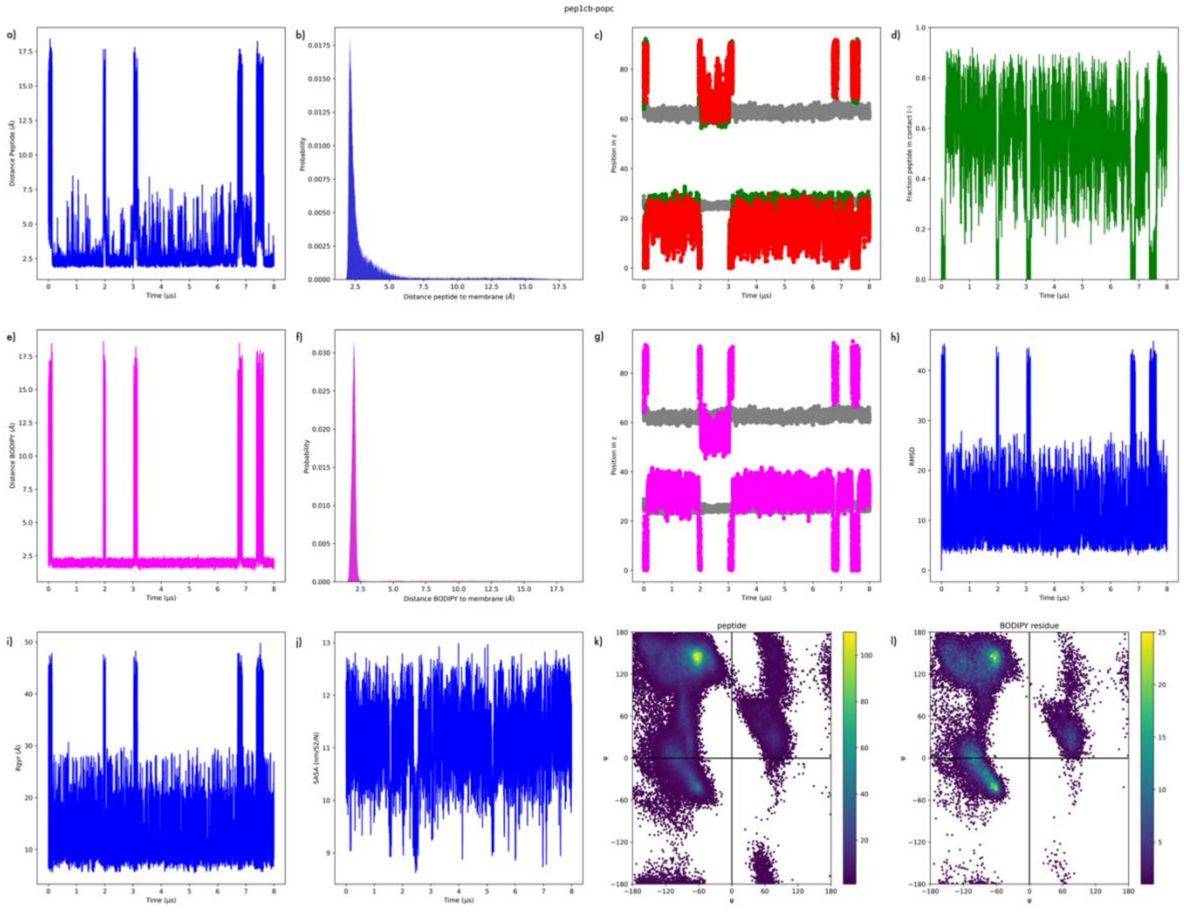

Figure S18 peptide 1 with BODIPY labelled cysteine residue in POPC membrane

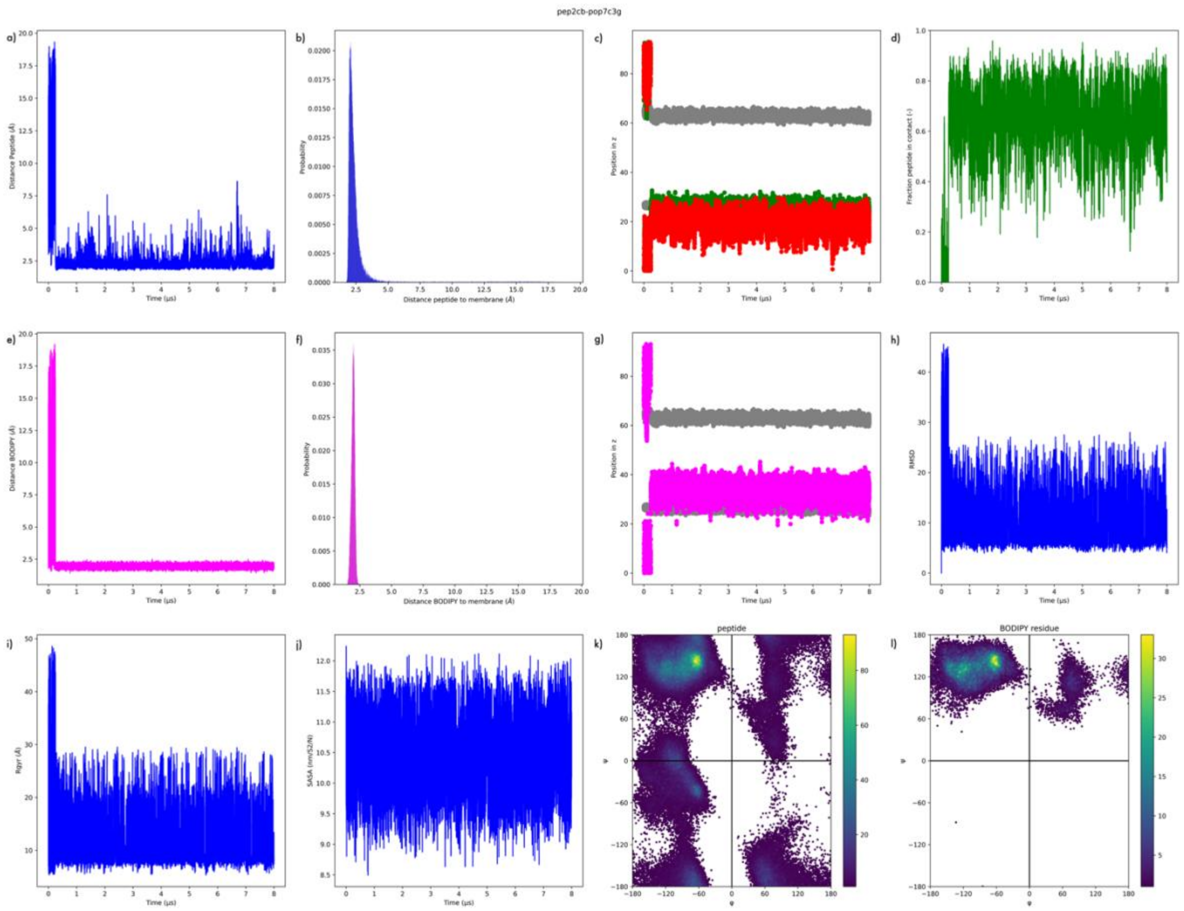

Figure S19 peptide 2 with BODIPY labelled cysteine residue in POPC/POPG membrane

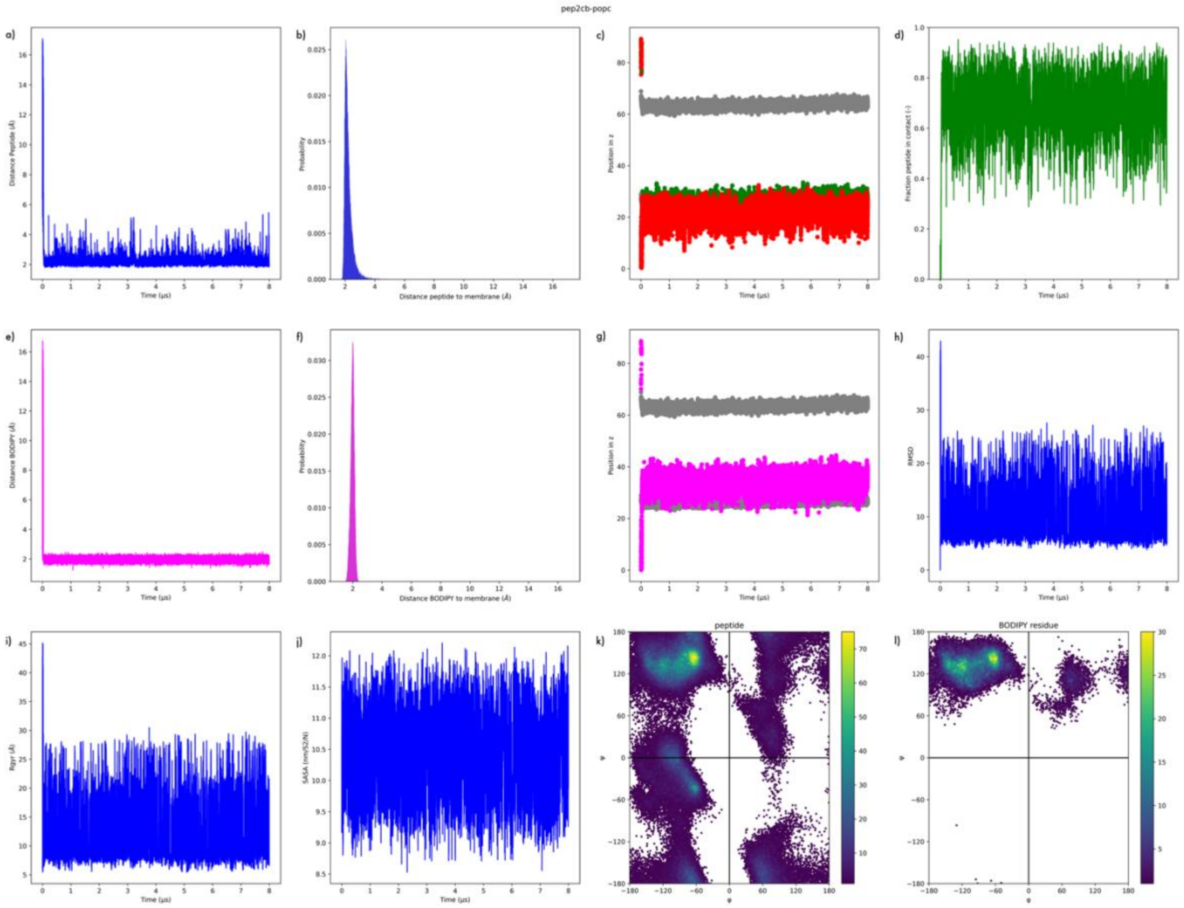

Figure S20 peptide 2 with BODIPY labelled cysteine residue in POPC membrane

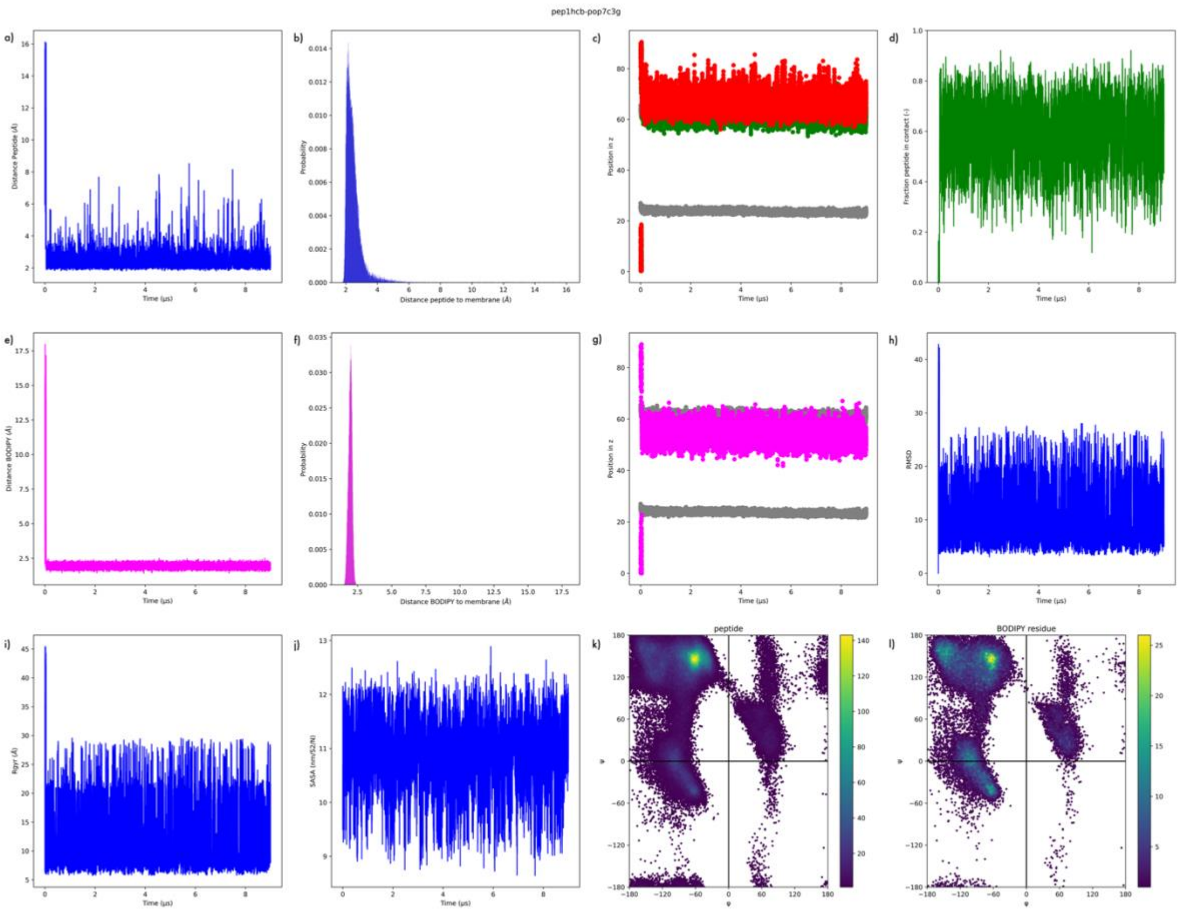

Figure S21 peptide 1 with BODIPY labelled homocysteine residue in POPC/POPG membrane

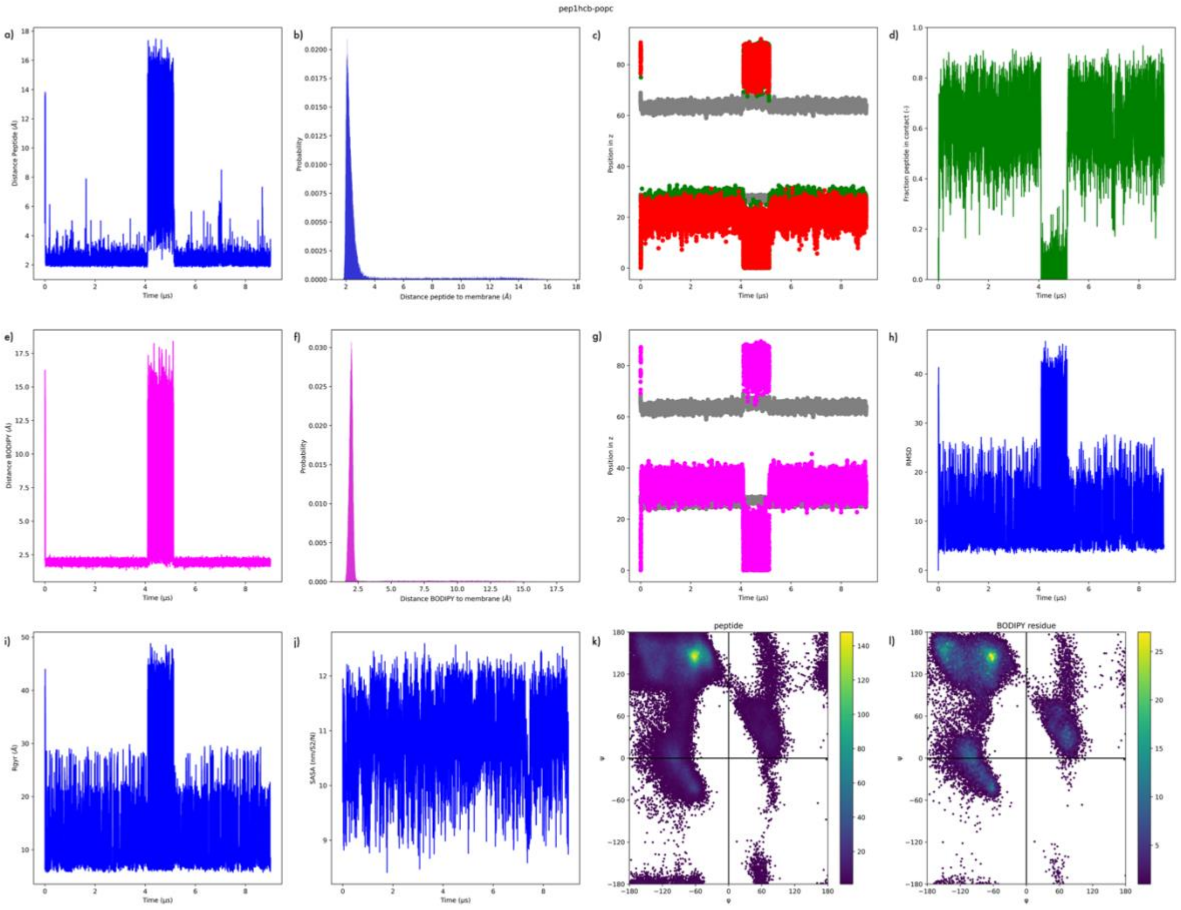

Figure S22 peptide 1 with BODIPY labelled homocysteine residue in POPC membrane

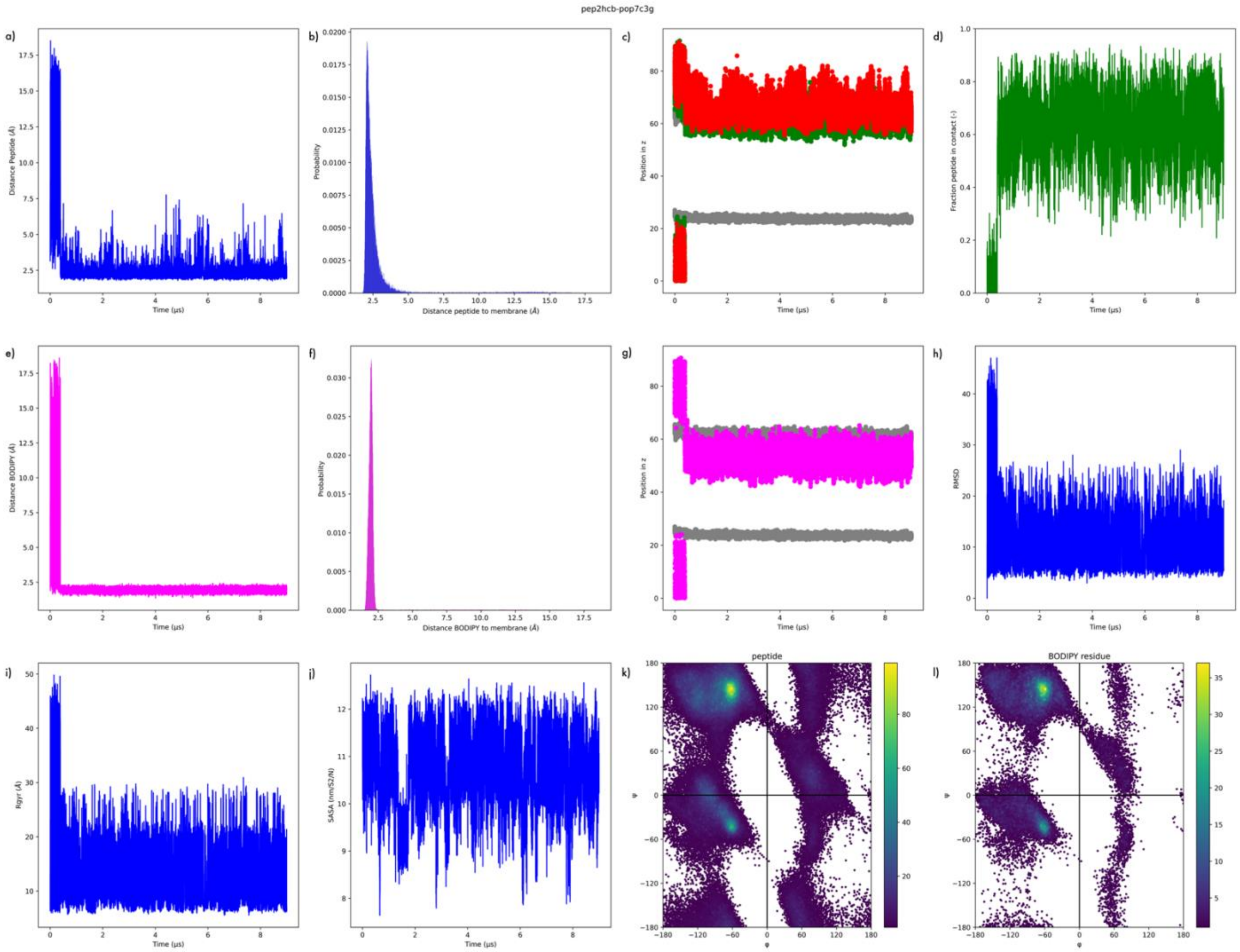

Figure S23 peptide 2 with BODIPY labelled homocysteine residue in POPC/POPG membrane

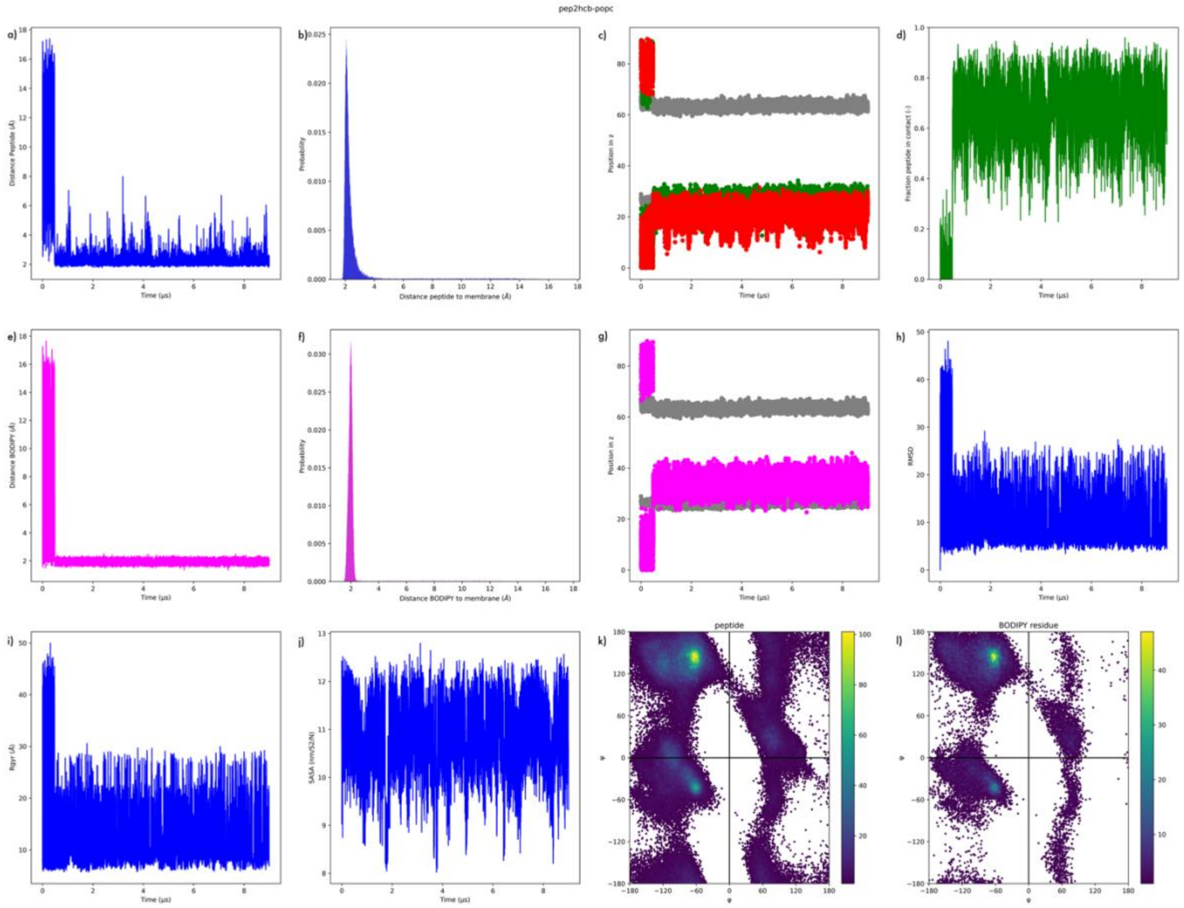

Figure S24 peptide 2 with BODIPY labelled homocysteine residue in POPC membrane

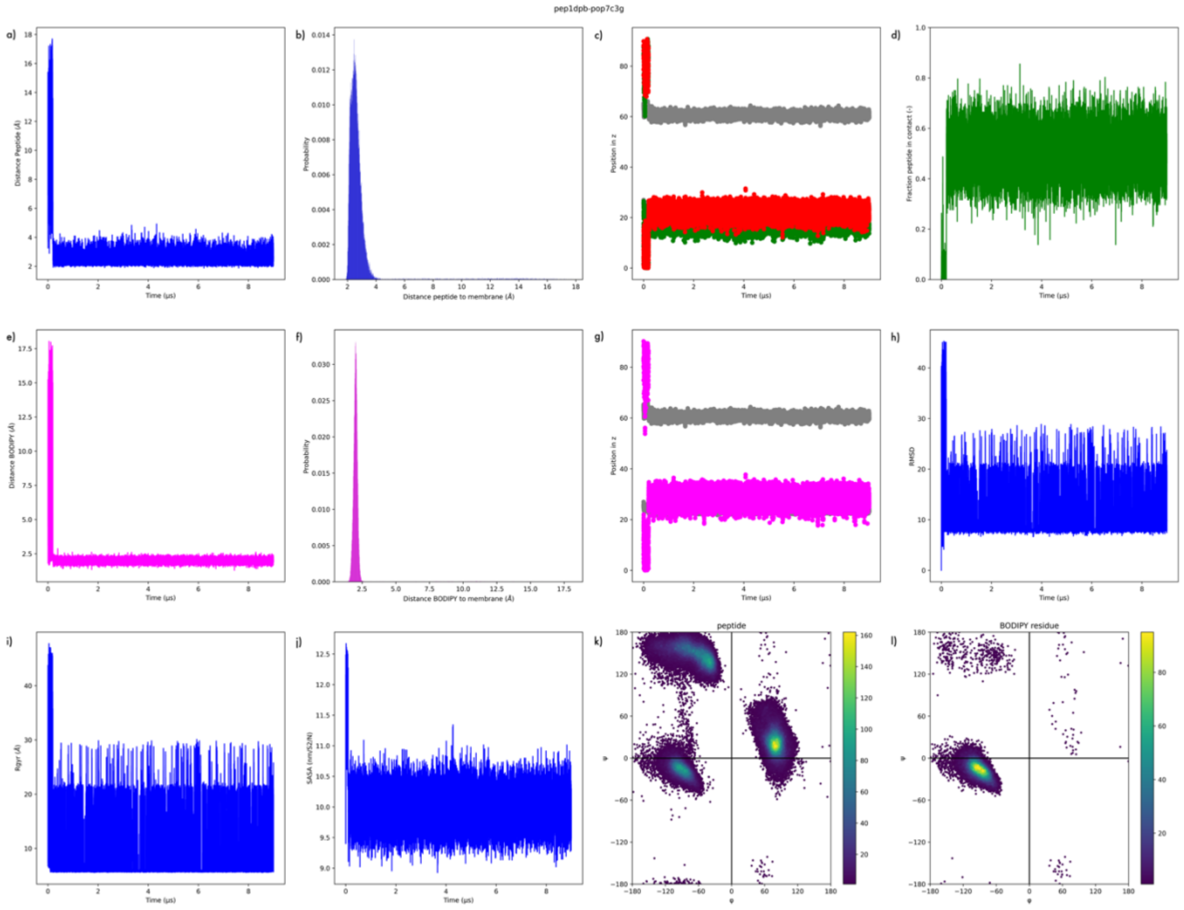

Figure S25 peptide 1 with BODIPY labelled 3-amino-Alanine residue in POPC/POPG membrane

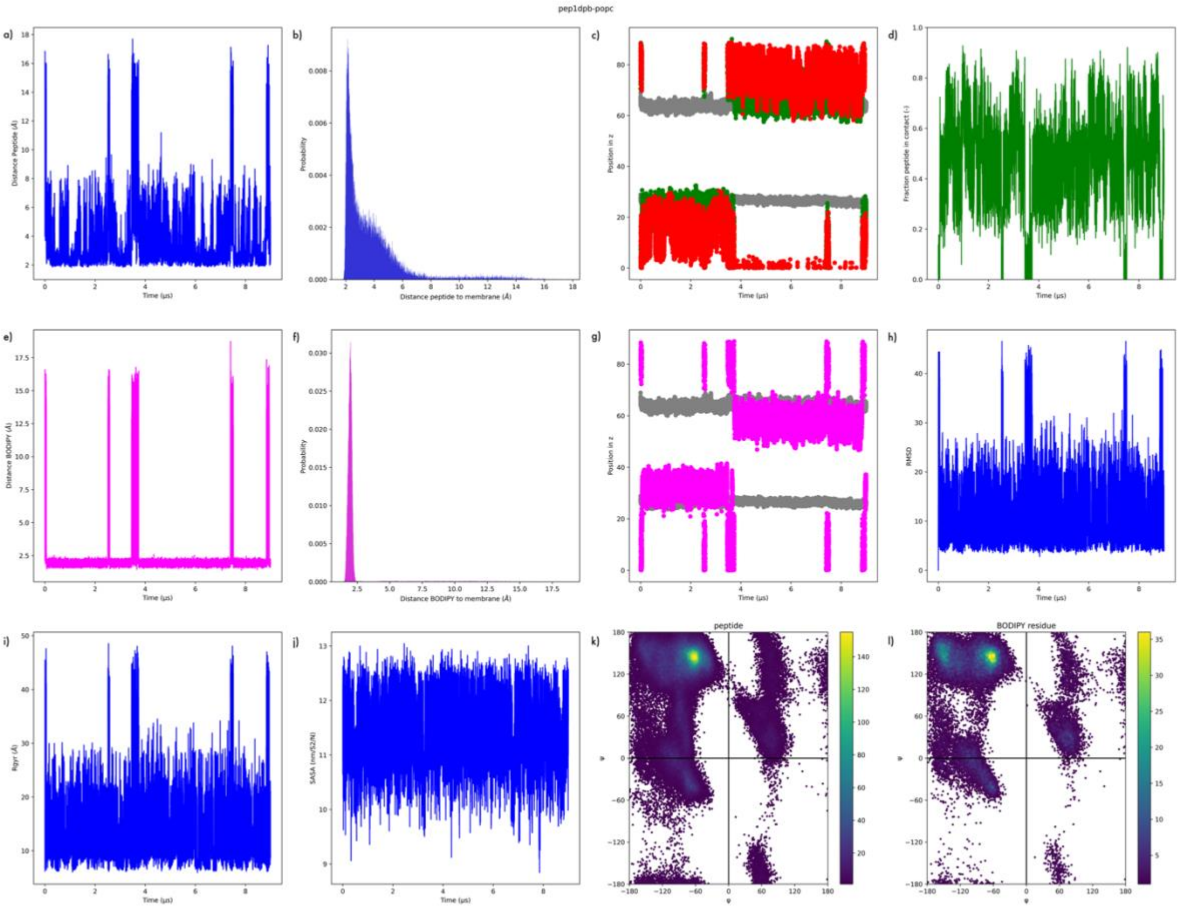

Figure S26 peptide 1 with BODIPY labelled 3-amino-Alanine residue in POPC membrane

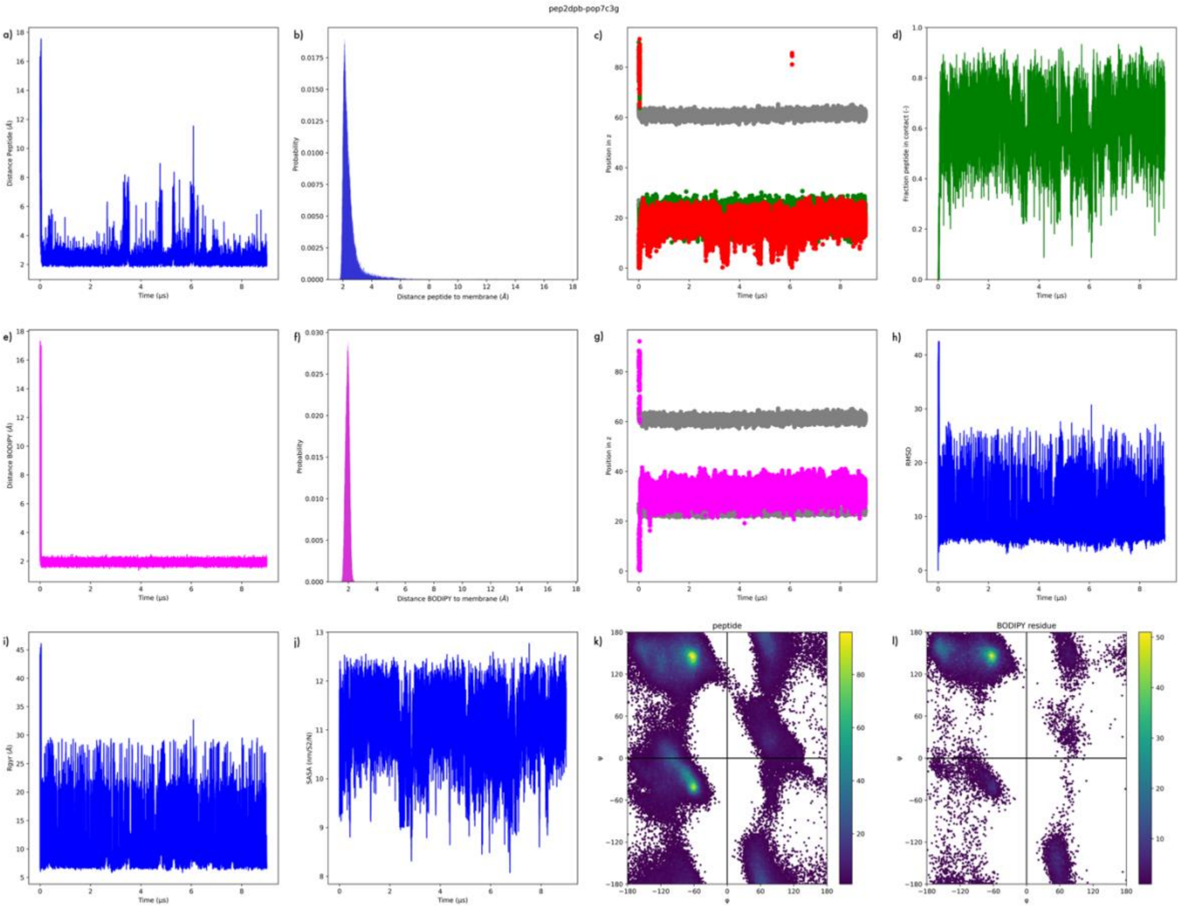

Figure S27 peptide 2 with BODIPY labelled 3-amino-Alanine residue in POPC/POPG membrane

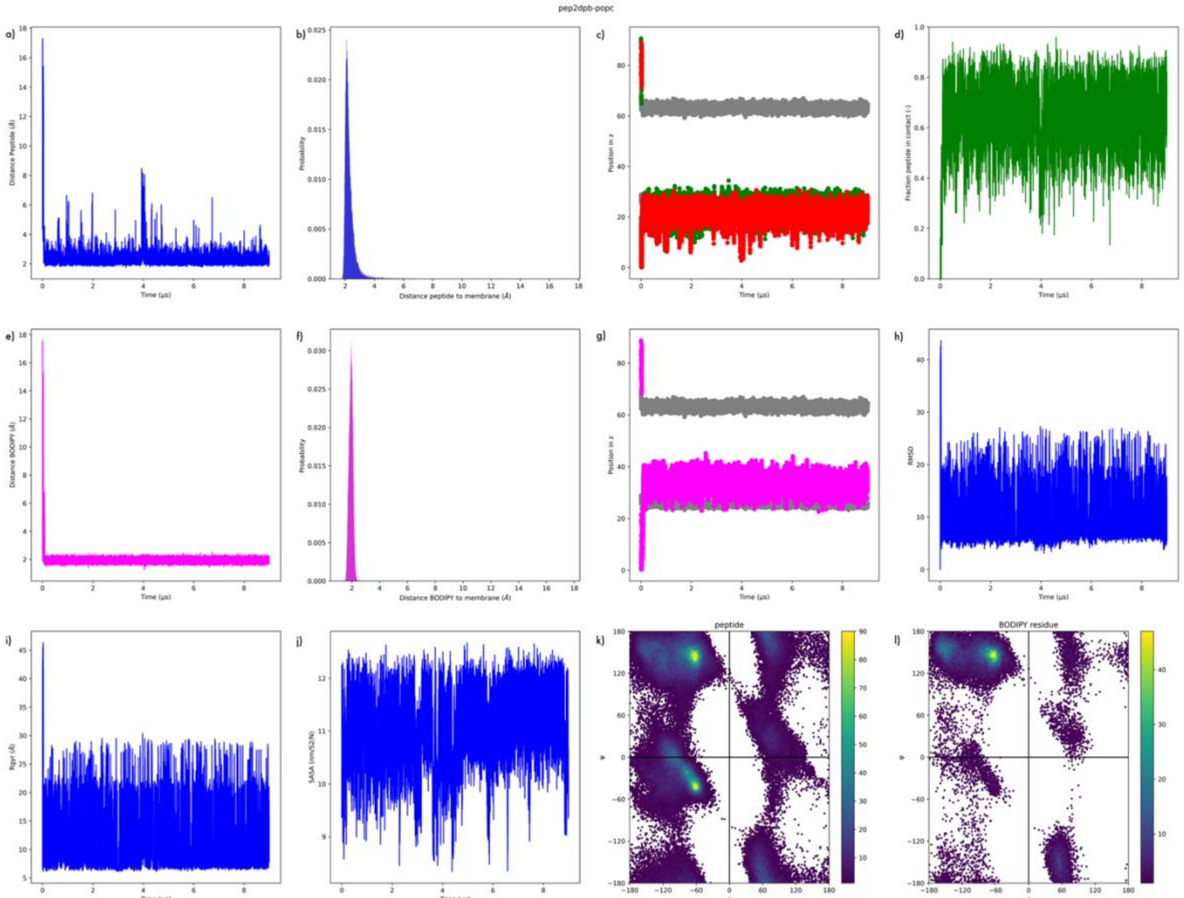

Figure S28 peptide 2 with BODIPY labelled 3-amino-Alanine residue in POPC membrane

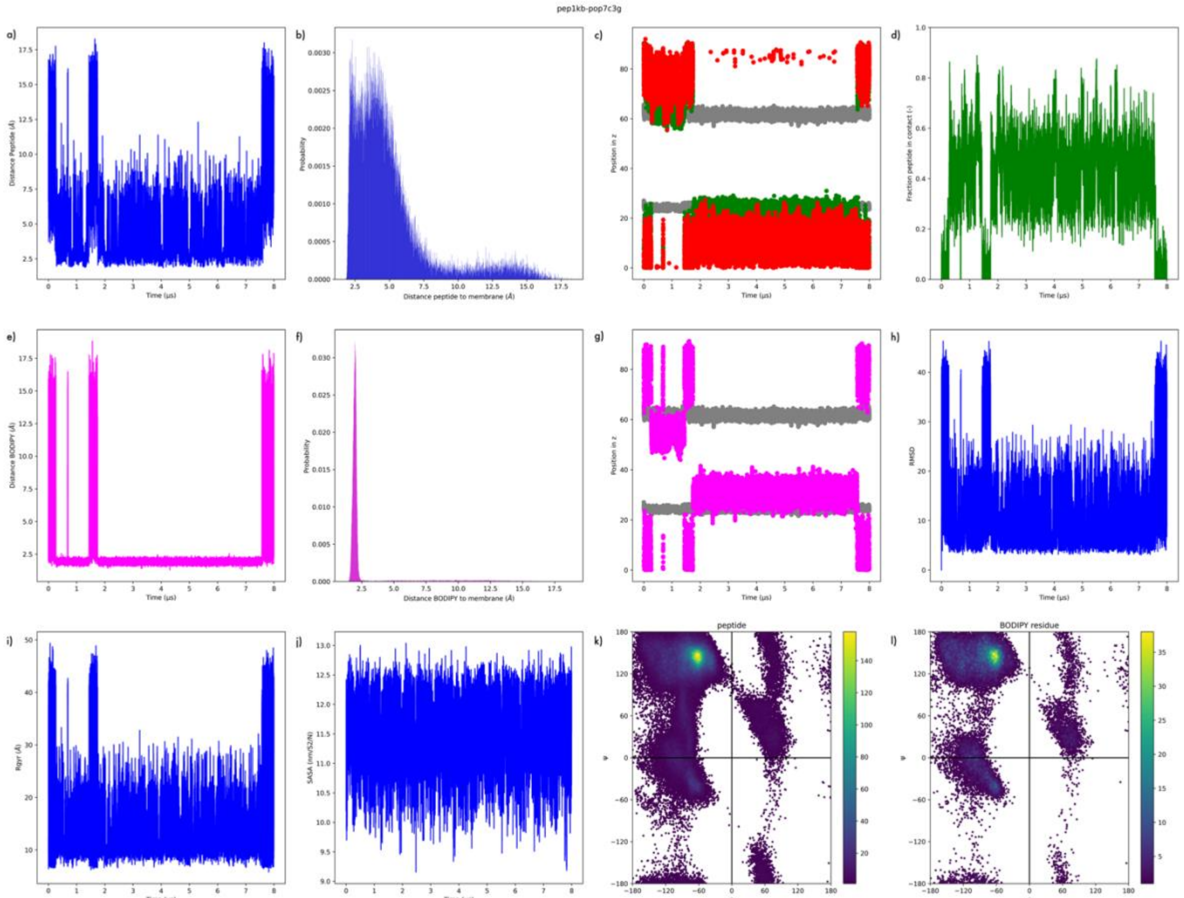

Figure S29 peptide 1 with BODIPY labelled Lysine residue in POPC/POPG membrane

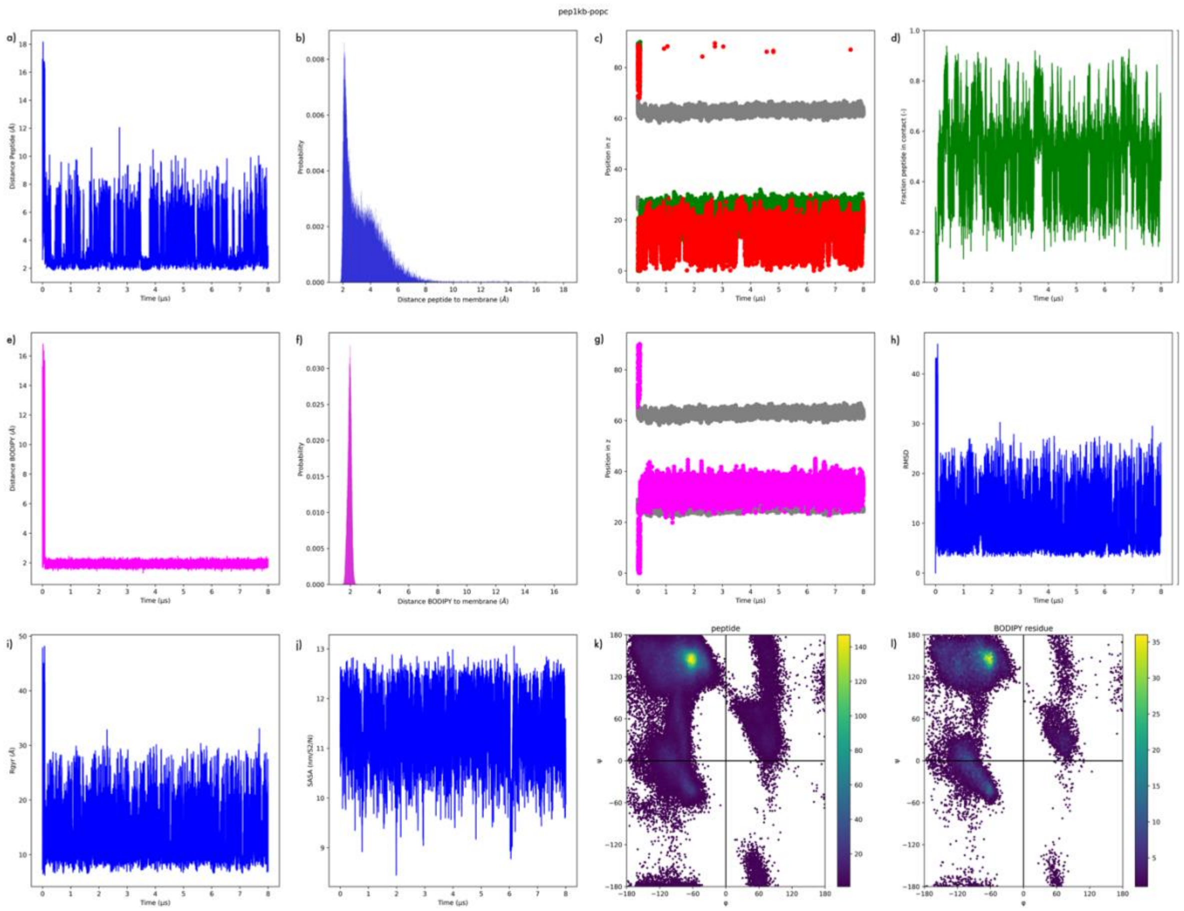

Figure S30 peptide 1 with BODIPY labelled Lysine residue in POPC membrane

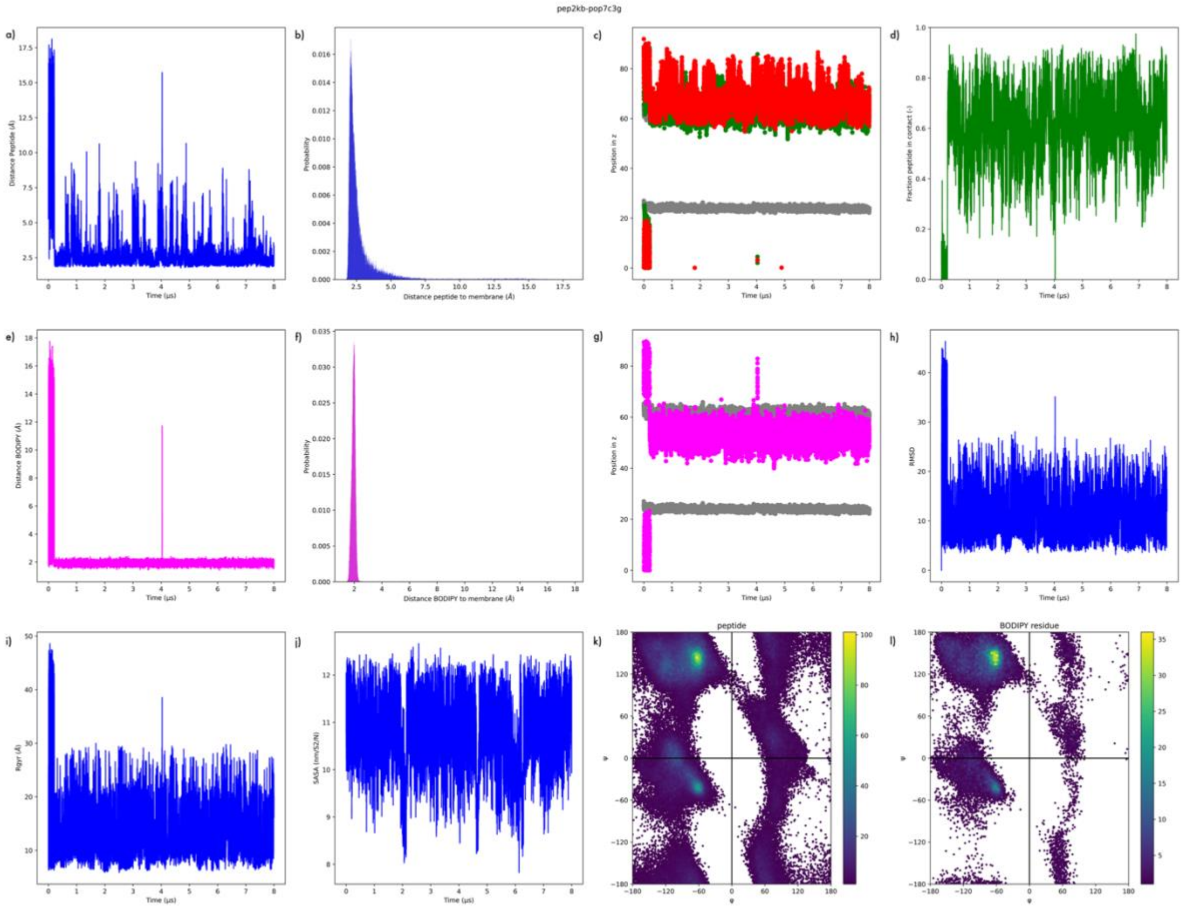

Figure S31 peptide 2 with BODIPY labelled Lysine residue in POPC/POPG membrane

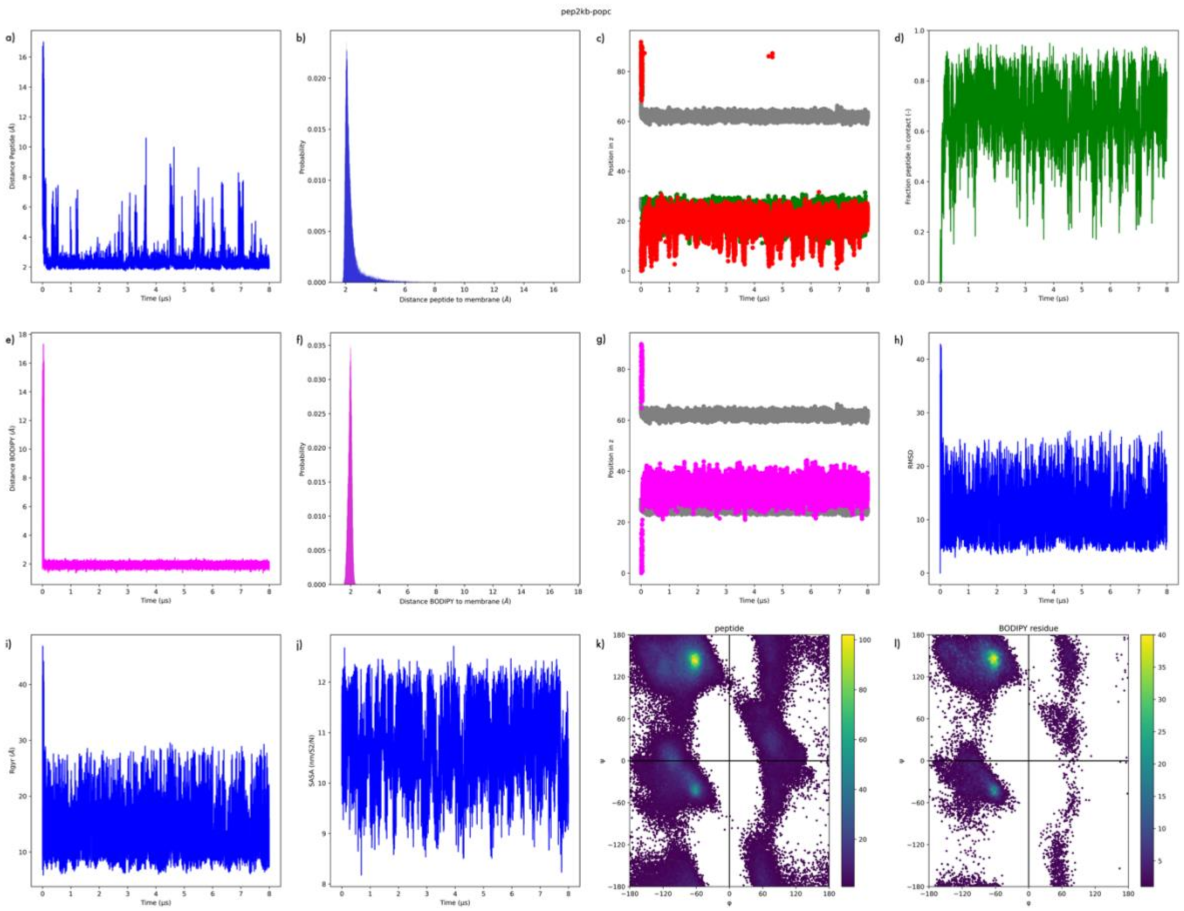

Figure S32 peptide 2 with BODIPY labelled Lysine residue in POPC membrane

Figure S33-S64: Total number of contacts between the POPC lipids (blue), POPG lipids (orange) and the whole membrane (green) and from left to right the whole peptide, residue 1, residue 2, residue 3, residue 4, residue 5, residue 6 and residue 7.

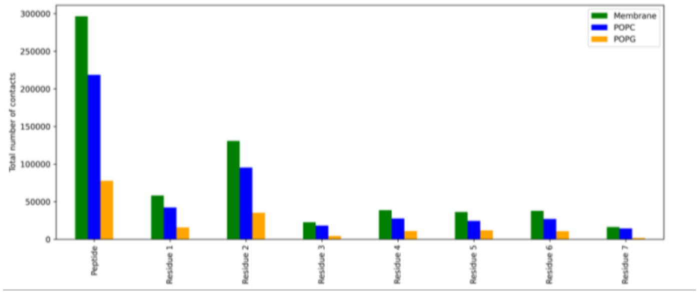

Figure S33 peptide 1 with tryptophan residue in POPC/POPG membrane

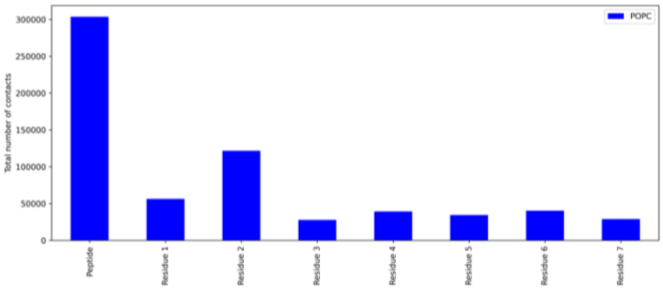

Figure S34 peptide 1 with tryptophan residue in POPC membrane

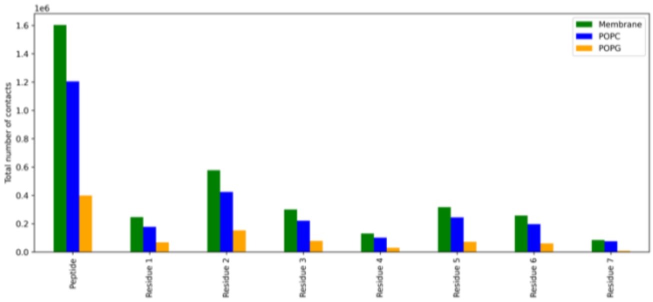

Figure S35 peptide 2 with tryptophan residue in POPC/POPG membrane

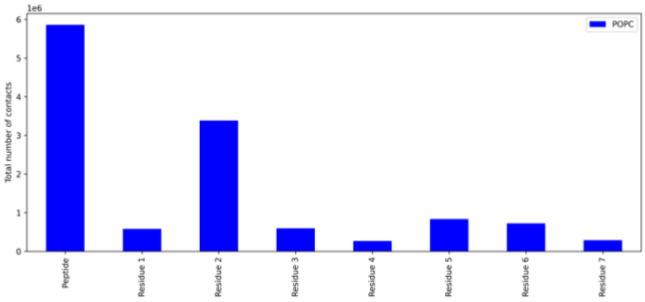

Figure S36 peptide 2 with tryptophan residue in POPC membrane

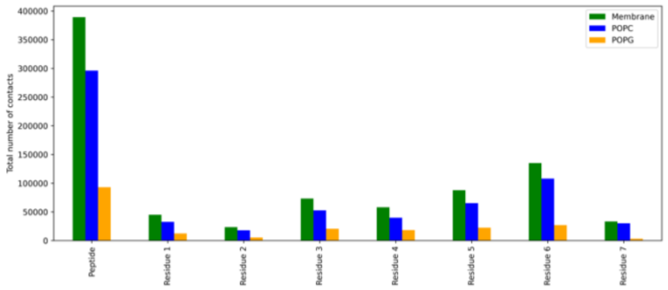

Figure S37 peptide 3 with tryptophan residue in POPC/POPG membrane

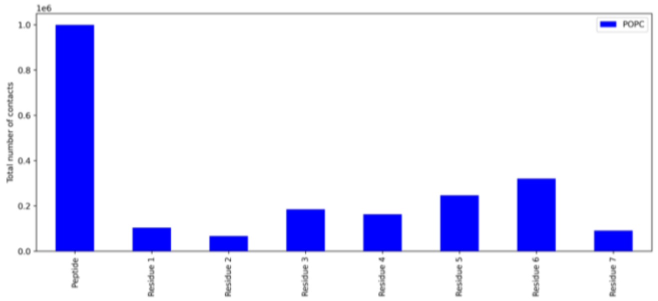

Figure S38 peptide 3 with tryptophan residue in POPC membrane

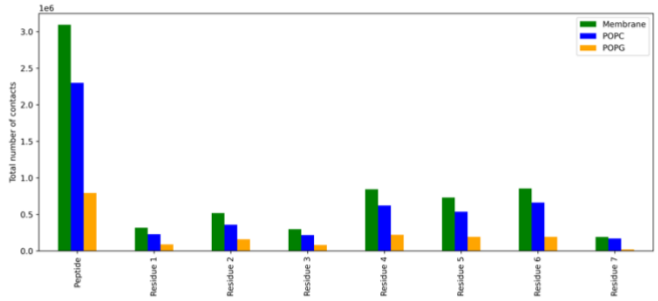

Figure S39 peptide 4 with tryptophan residue in POPC/POPG membrane

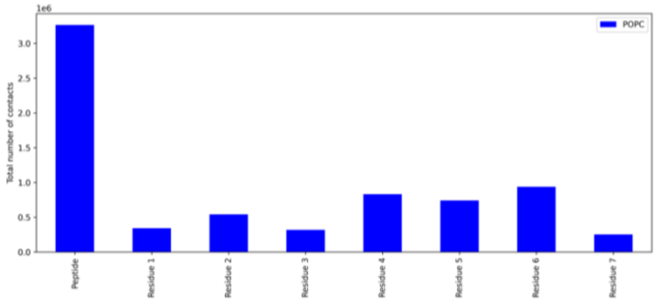

Figure S40 peptide 4 with tryptophan residue in POPC membrane

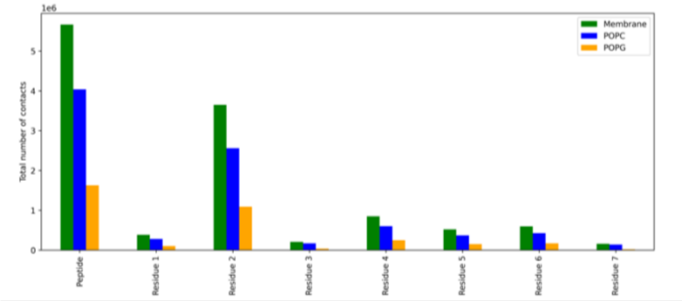

Figure S41 peptide 1 with BODIPY labelled tryptophan residue in POPC/POPG membrane

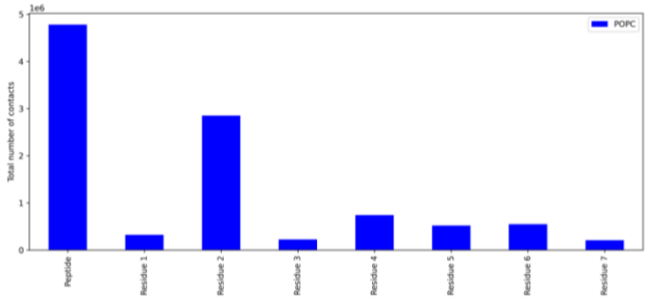

Figure S42 peptide 1 with BODIPY labelled tryptophan residue in POPC membrane

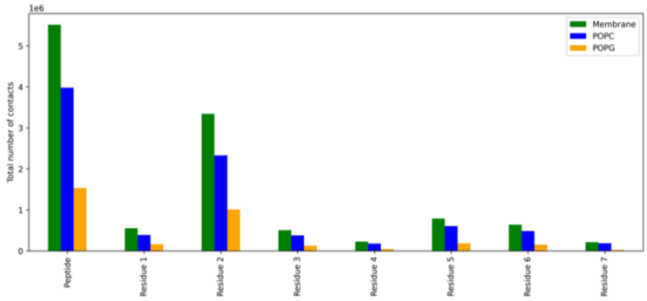

Figure S43 peptide 2 with BODIPY labelled tryptophan residue in POPC/POPG membrane

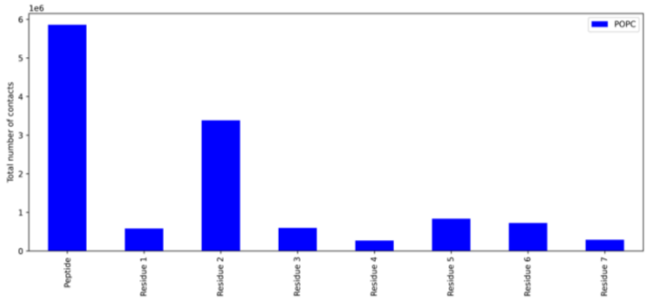

Figure S44 peptide 2 with BODIPY labelled tryptophan residue in POPC membrane

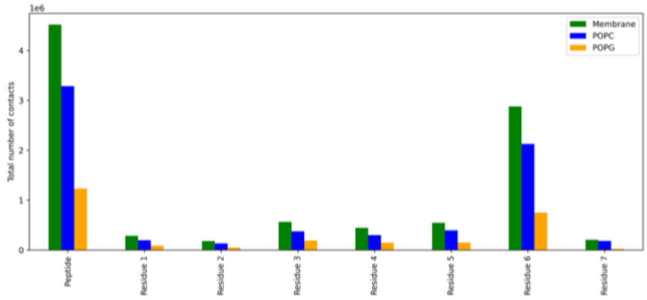

Figure S45 peptide 3 with BODIPY labelled tryptophan residue in POPC/POPG membrane

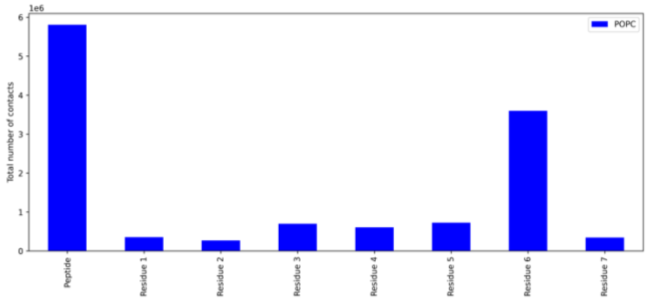

Figure S46 peptide 3 with BODIPY labelled tryptophan residue in POPC membrane

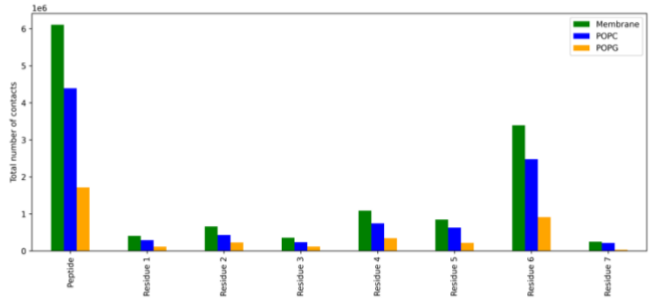

Figure S47 peptide 4 with BODIPY labelled tryptophan residue in POPC/POPG membrane

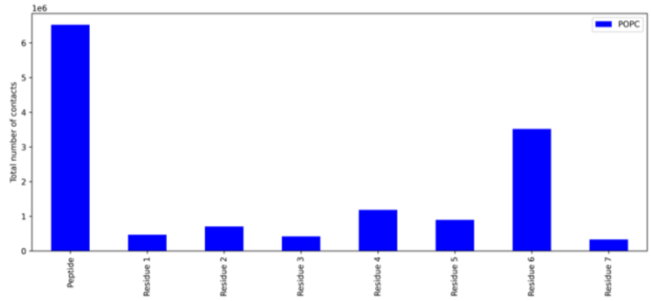

Figure S48 peptide 4 with BODIPY labelled tryptophan residue in POPC membrane

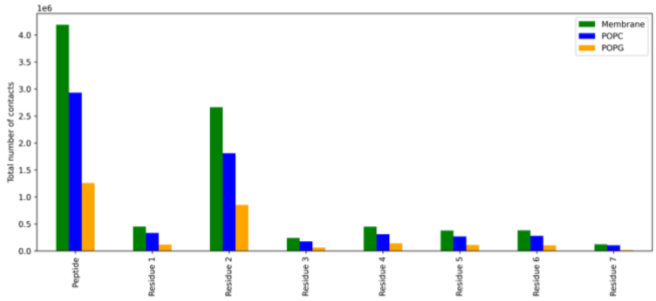

Figure S49 peptide 1 with BODIPY labelled cysteine residue in POPC/POPG membrane

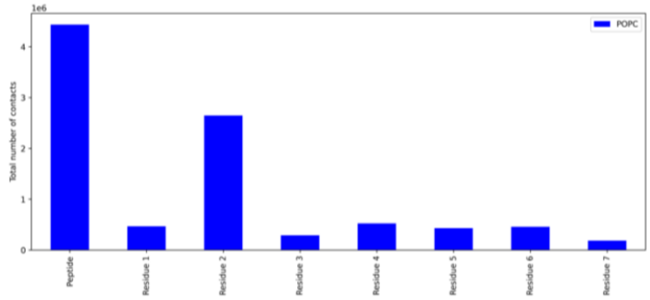

Figure S50 peptide 1 with BODIPY labelled cysteine residue in POPC membrane

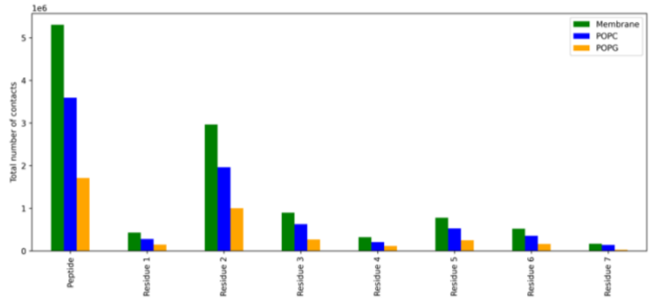

Figure S51 peptide 2 with BODIPY labelled cysteine residue in POPC/POPG membrane

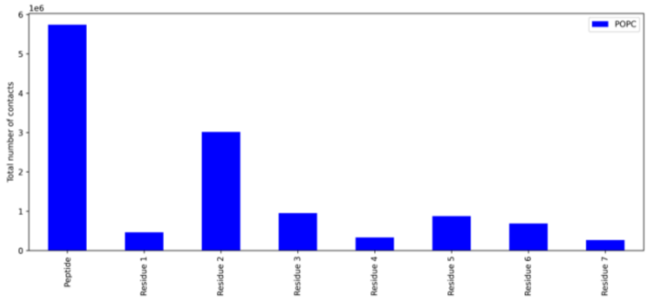

Figure S52 peptide 2 with BODIPY labelled cysteine residue in POPC membrane

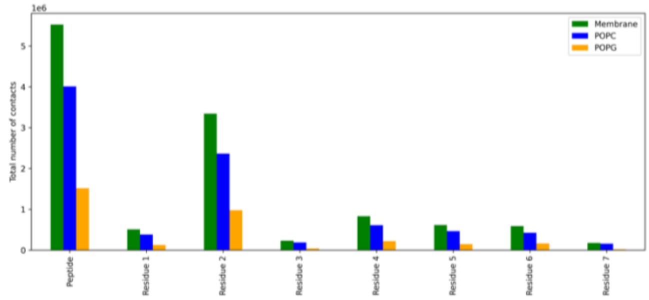

Figure S53 peptide 1 with BODIPY labelled homocysteine residue in POPC/POPG membrane

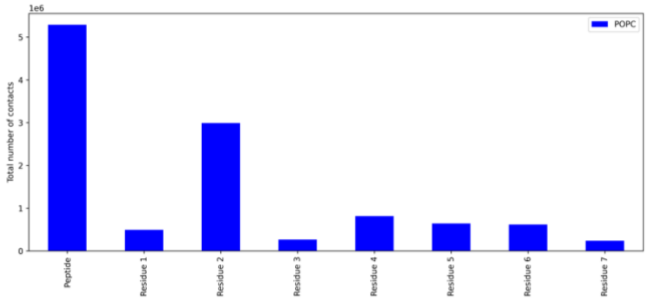

Figure S54 peptide 1 with BODIPY labelled homocysteine residue in POPC membrane

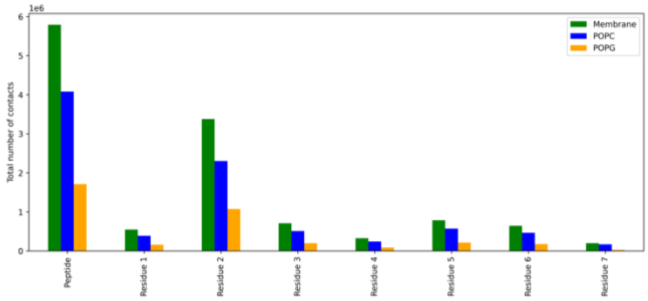

Figure S55 peptide 2 with BODIPY labelled homocysteine residue in POPC/POPG membrane

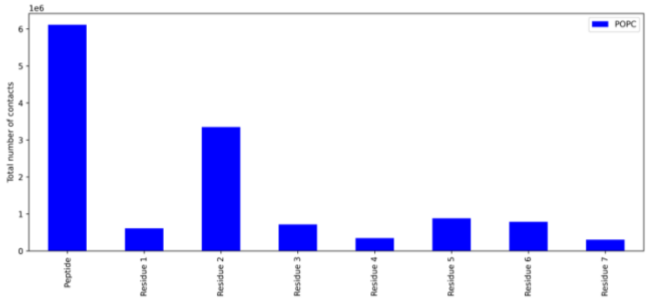

Figure S56 peptide 2 with BODIPY labelled homocysteine residue in POPC membrane

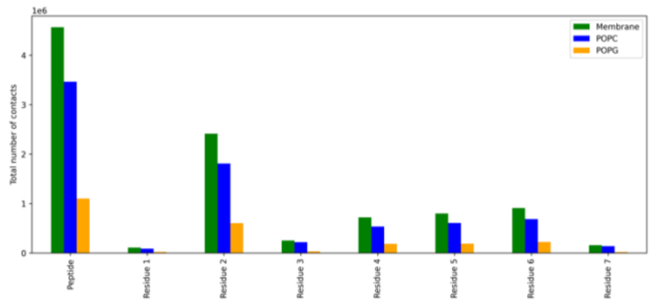

Figure S57 peptide 1 with BODIPY labelled 3-amino-Alanine residue in POPC/POPG membrane

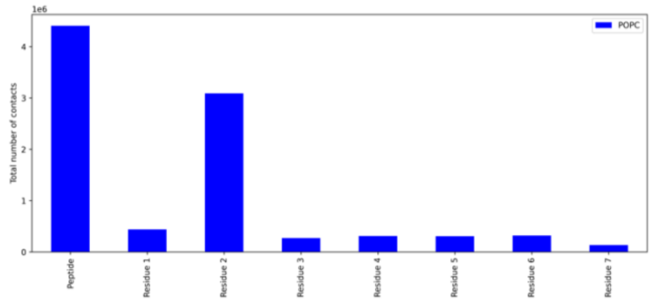

Figure S58 peptide 1 with BODIPY labelled 3-amino-Alanine residue in POPC membrane

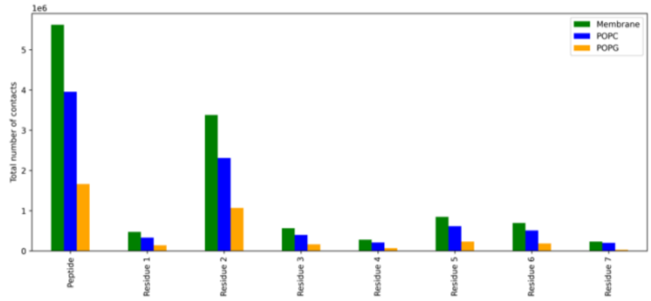

Figure S59 peptide 2 with BODIPY labelled 3-amino-Alanine residue in POPC/POPG membrane

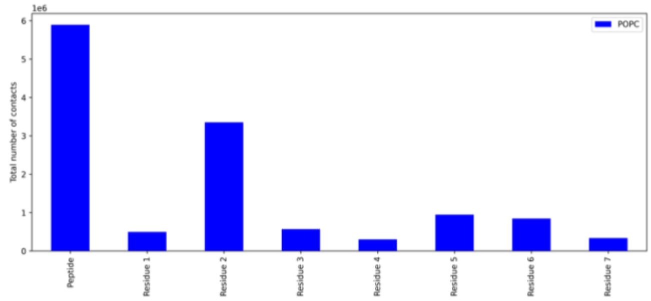

Figure S60 peptide 2 with BODIPY labelled 3-amino-Alanine residue in POPC membrane

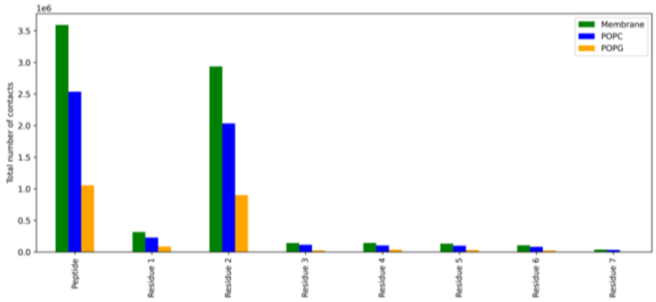

Figure S61 peptide 1 with BODIPY labelled Lysine residue in POPC/POPG membrane

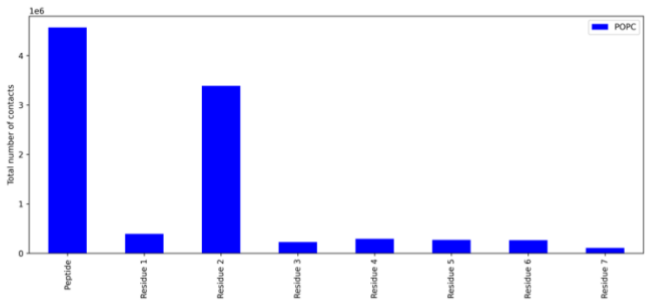

Figure S62 peptide 1 with BODIPY labelled Lysine residue in POPC membrane

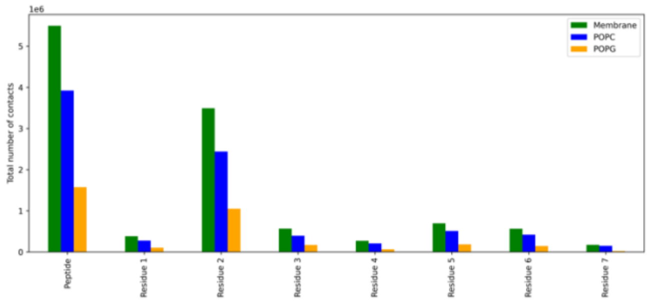

Figure S63 peptide 2 with BODIPY labelled Lysine residue in POPC/POPG membrane

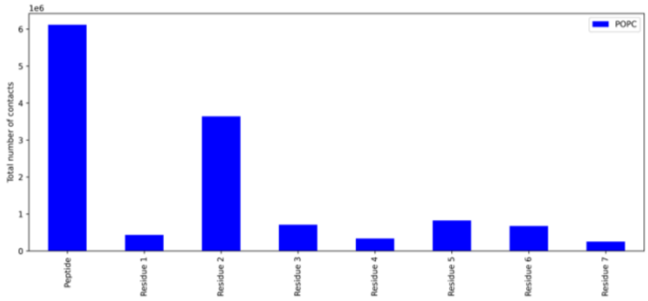

Figure S64 peptide 2 with BODIPY labelled Lysine residue in POPC membrane

Figure S65-S96: Probabilities of finding atoms at the z-directional positions for **a)** C-terminal carbon (red) and N-terminal nitrogen (green) **b)** C-alpha carbon for residue 1 **c)** C-alpha carbon for residue 2 in blue and for **Figures 73-76 and 81-96** boron atom of BODIPY labelled residue in magenta **d)** C-alpha carbon for residue 3 **e)** C-alpha carbon for residue 4 **f)** C-alpha carbon for residue 5 **g)** C-alpha carbon for residue 6 **h)** C-alpha carbon for residue 7 in blue and for **Figures S77-S80** boron atom of the BODIPY labelled residue in magenta. The black vertical lines represent the average position on the phosphor atom of the lipid headgroups.

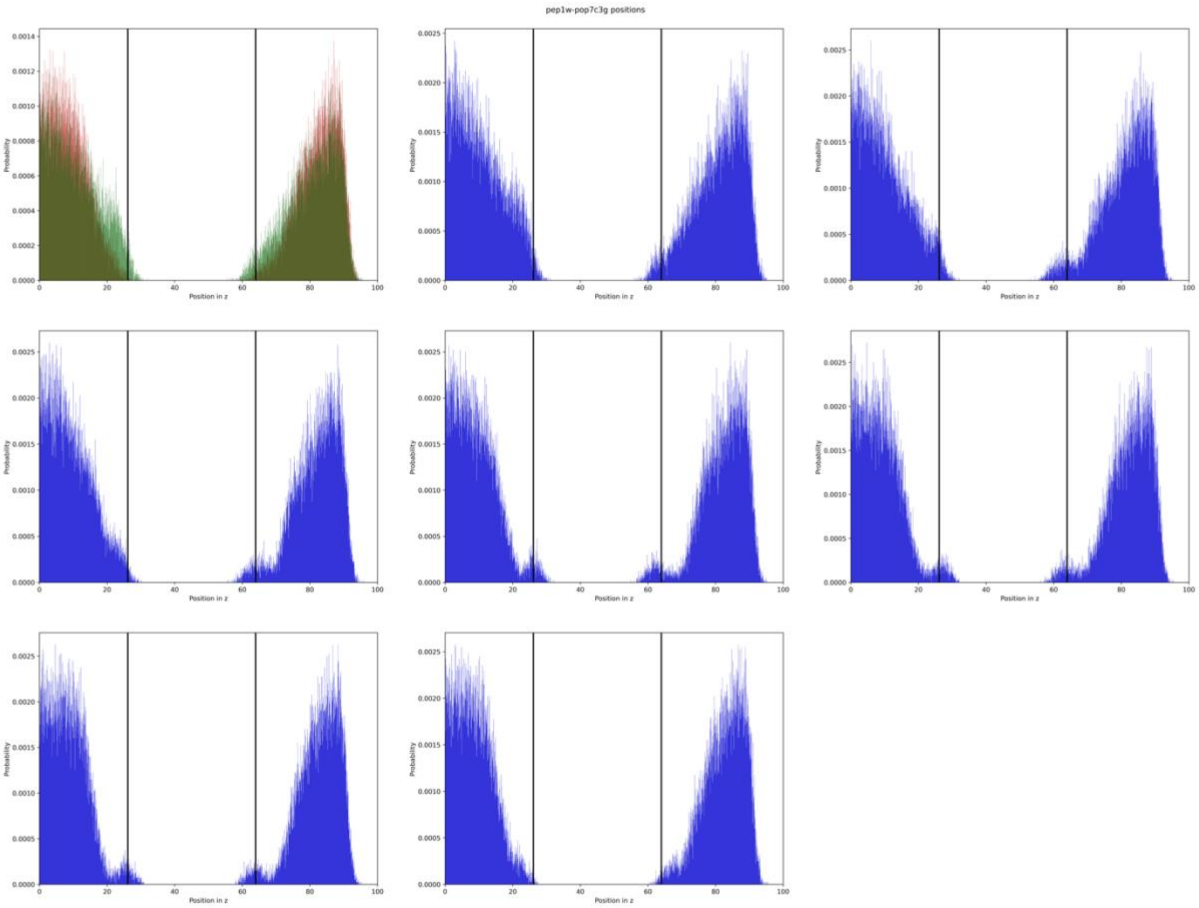

Figure S65 peptide 1 with tryptophan residue in POPC/POPG membrane

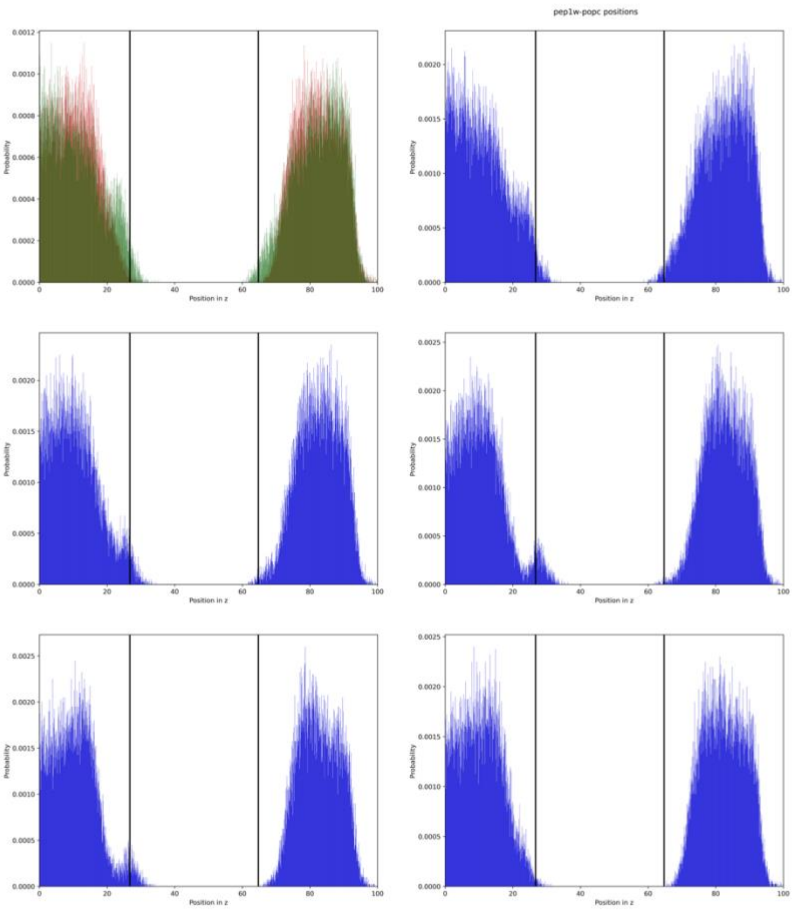

Figure S66 peptide 1 with tryptophan residue in POPC membrane

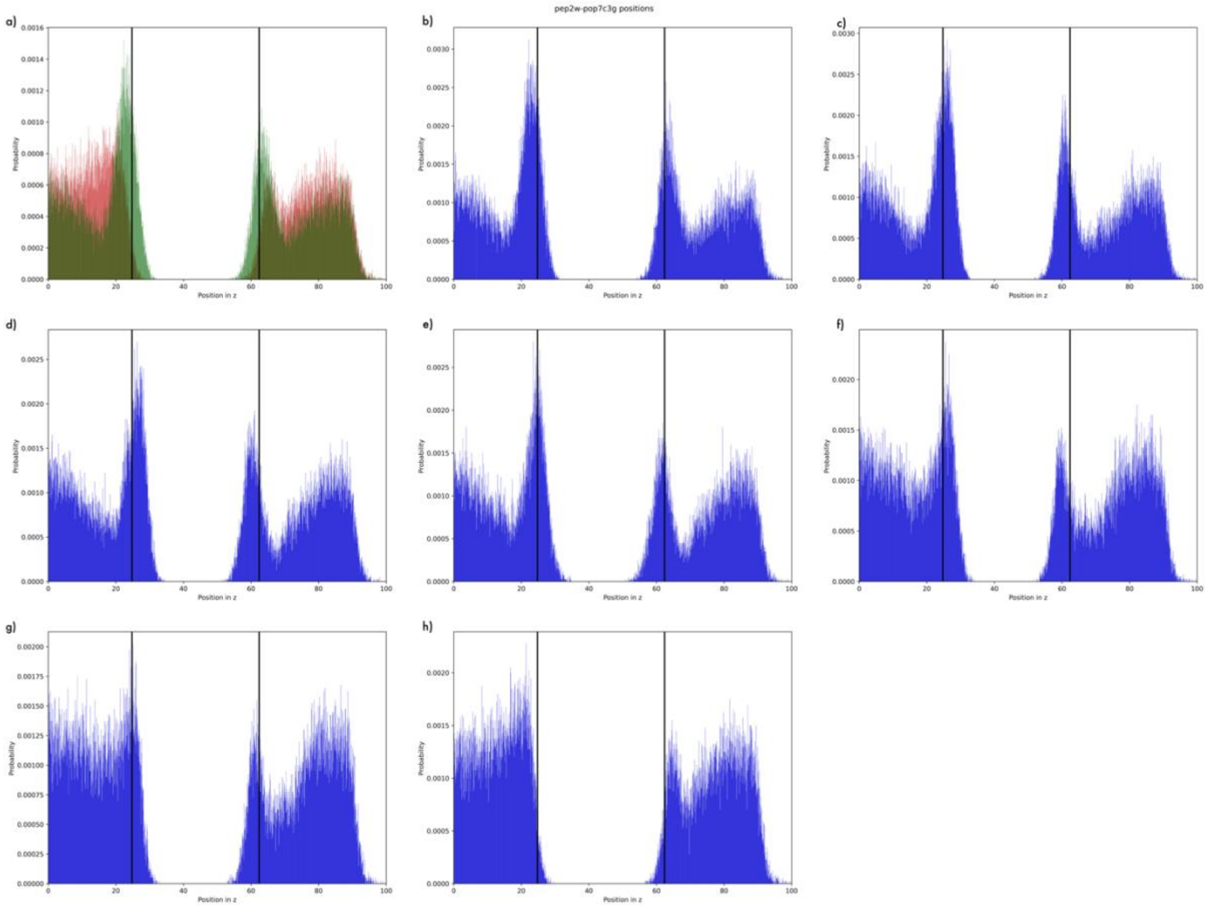

Figure S67 peptide 2 with tryptophan residue in POPC/POPG membrane

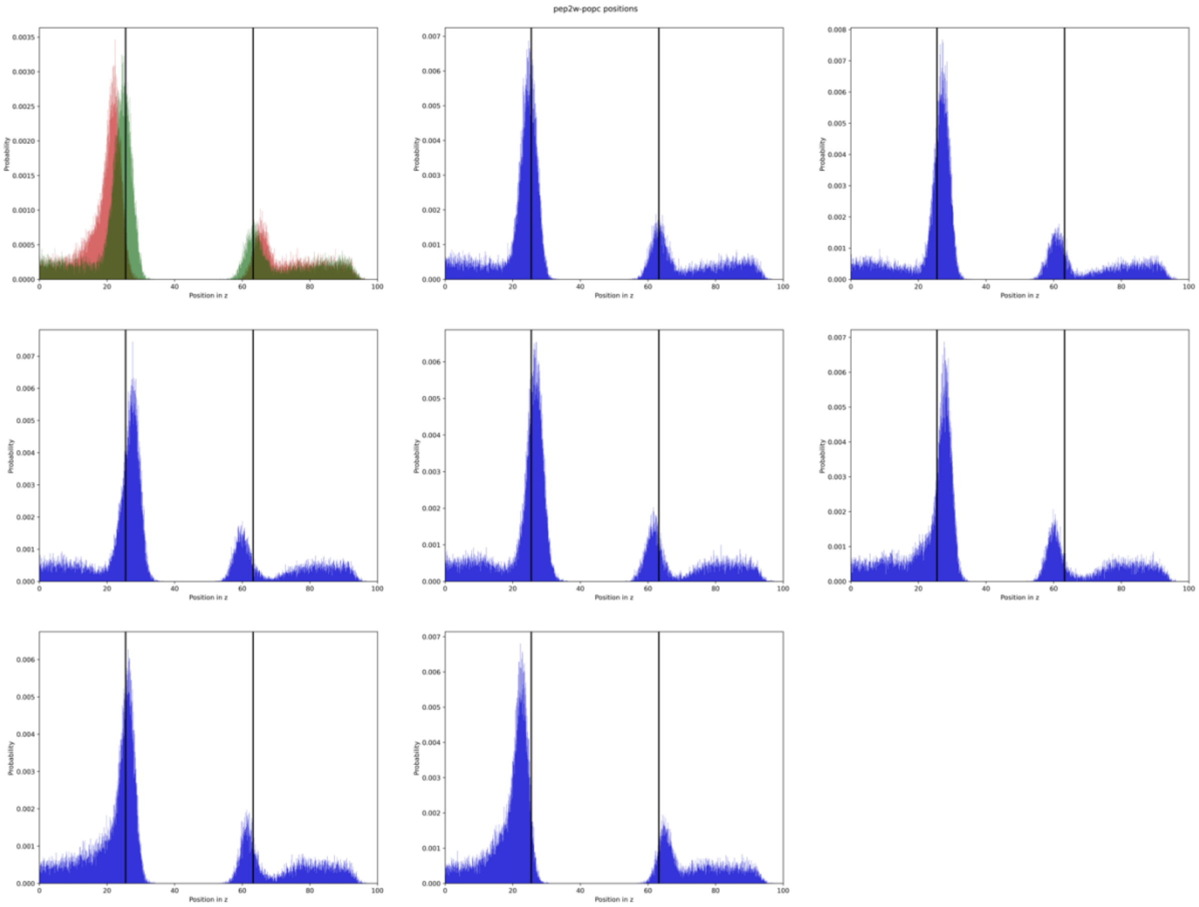

Figure S68 peptide 2 with tryptophan residue in POPC membrane

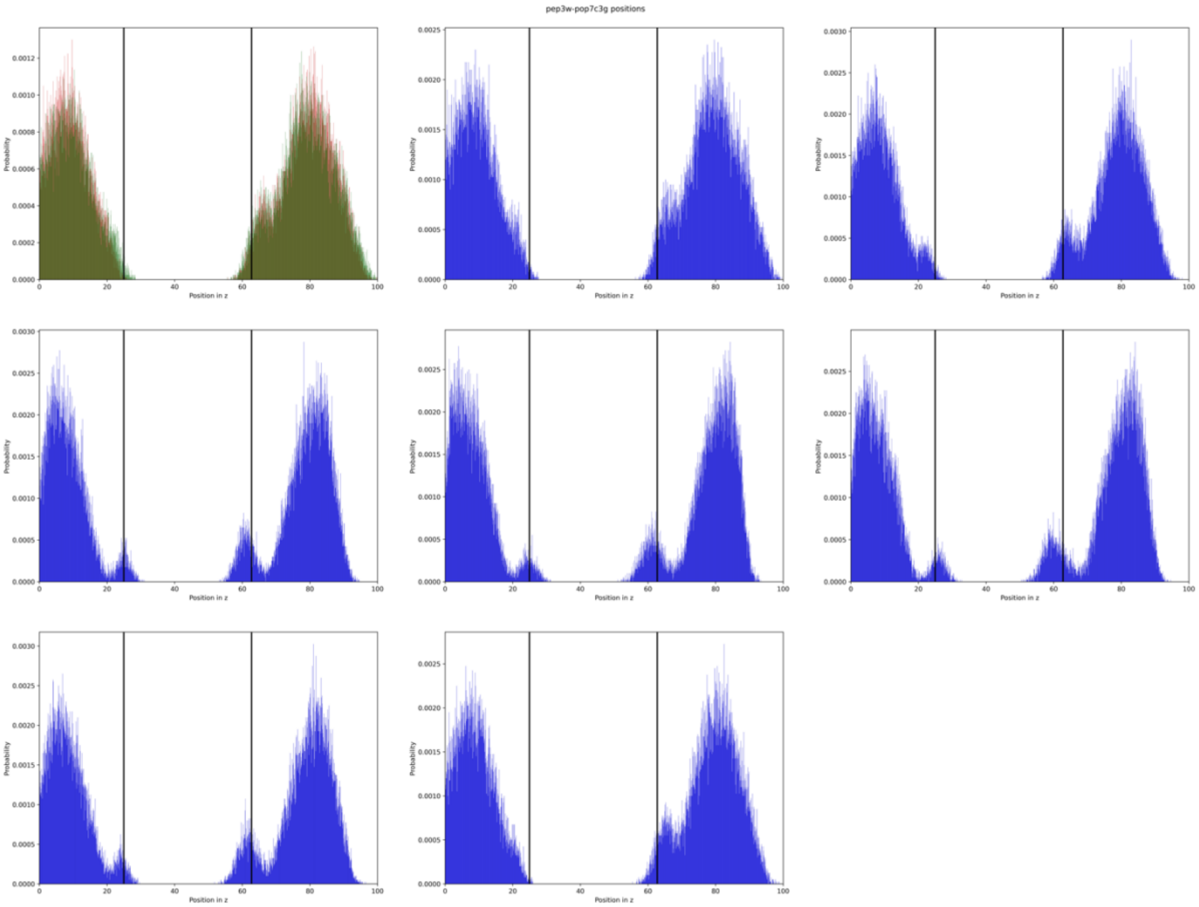

Figure S69 peptide 3 with tryptophan residue in POPC/POPG membrane

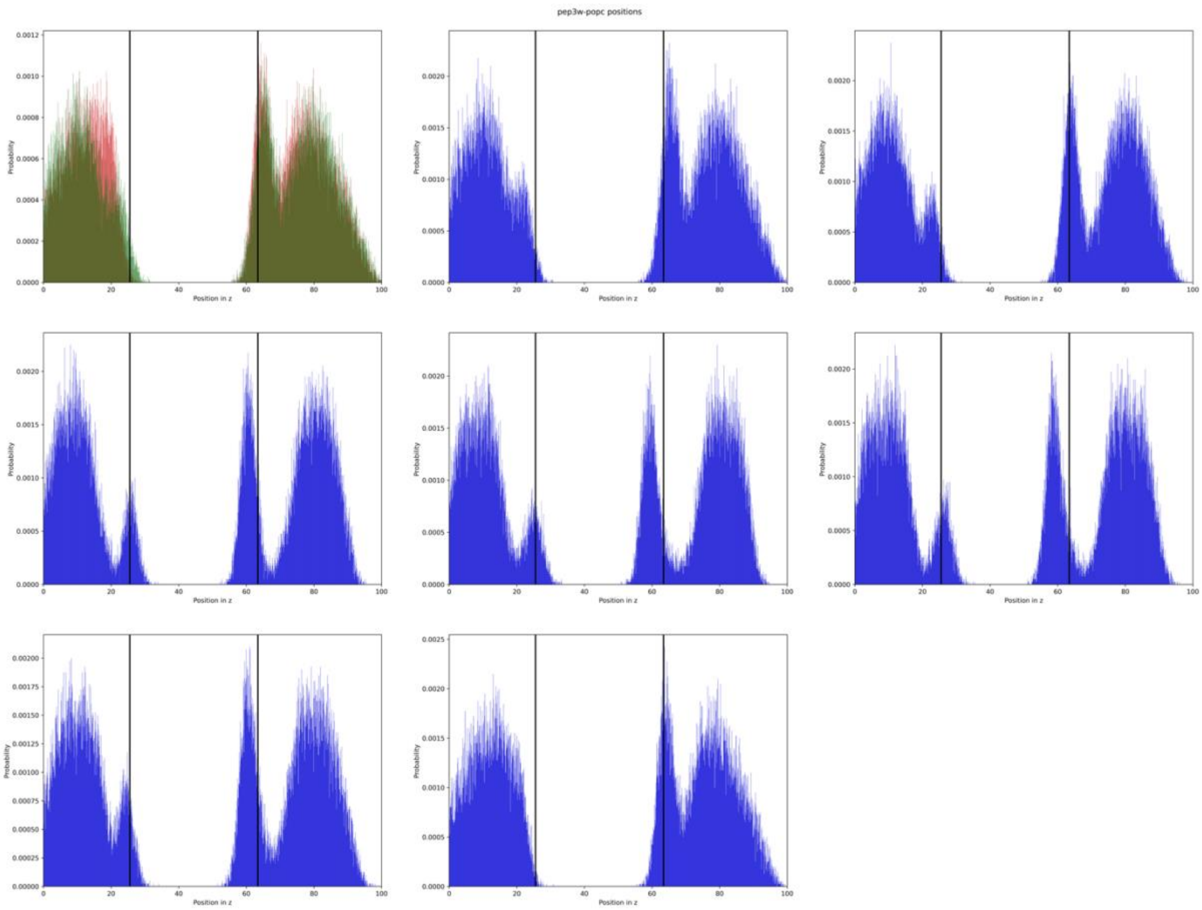

Figure S70 peptide 3 with tryptophan residue in POPC membrane

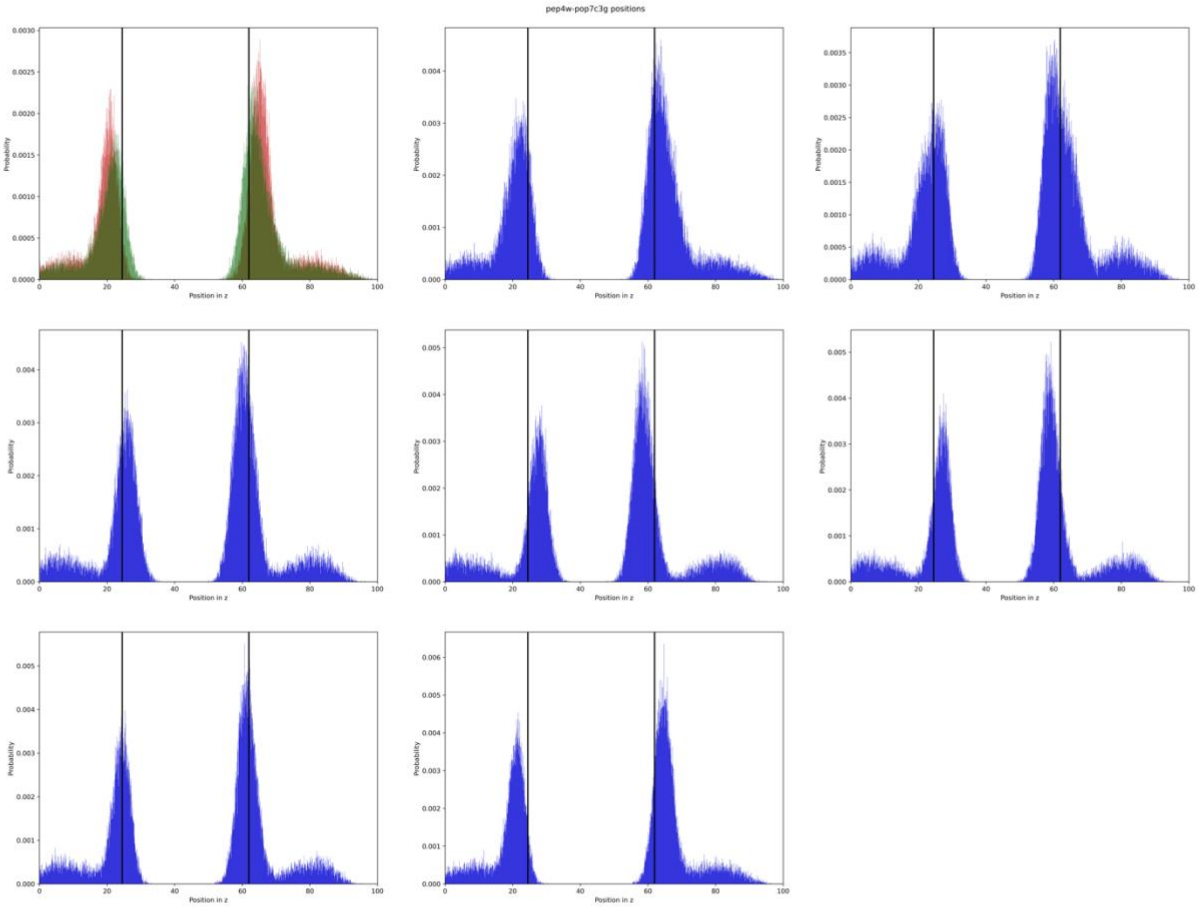

Figure S71 peptide 4 with tryptophan residue in POPC/POPG membrane

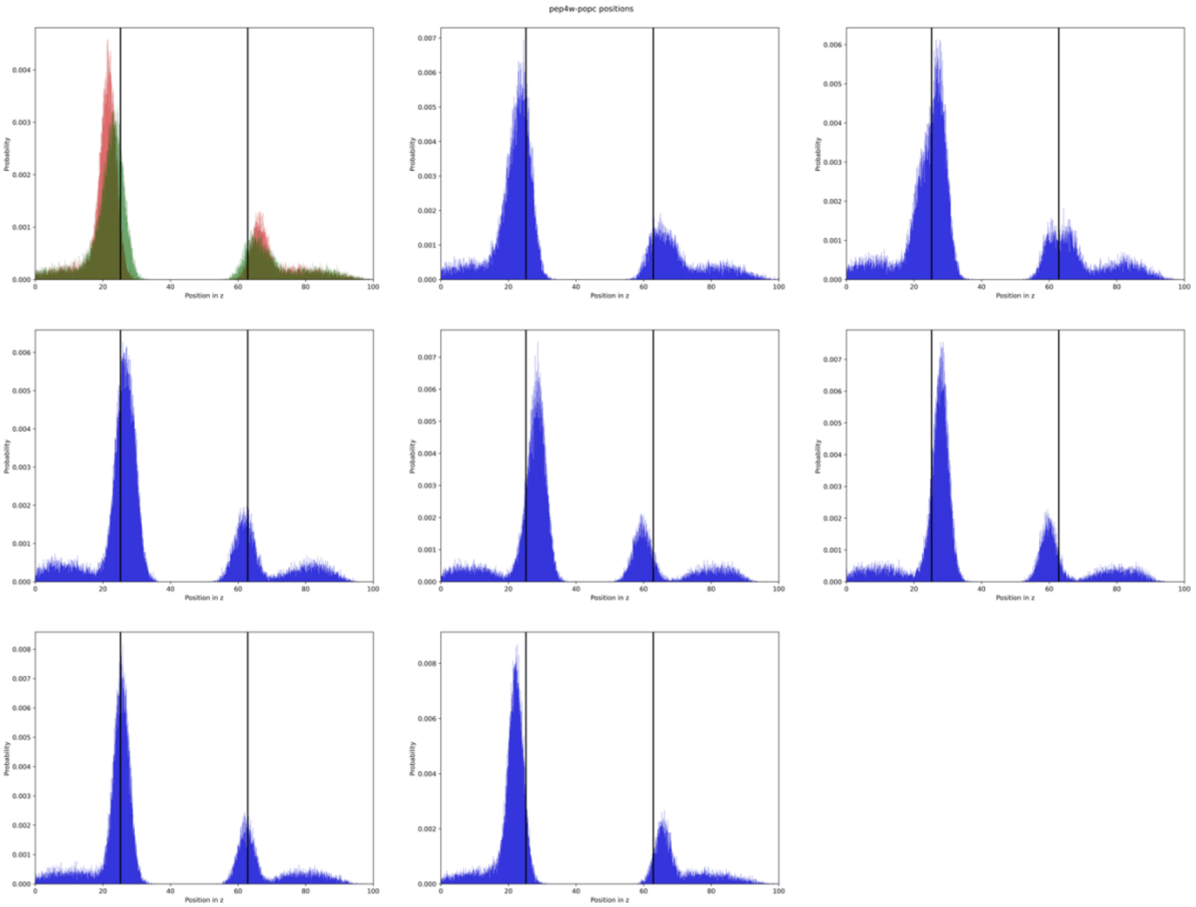

Figure S72 peptide 4 with tryptophan residue in POPC membrane

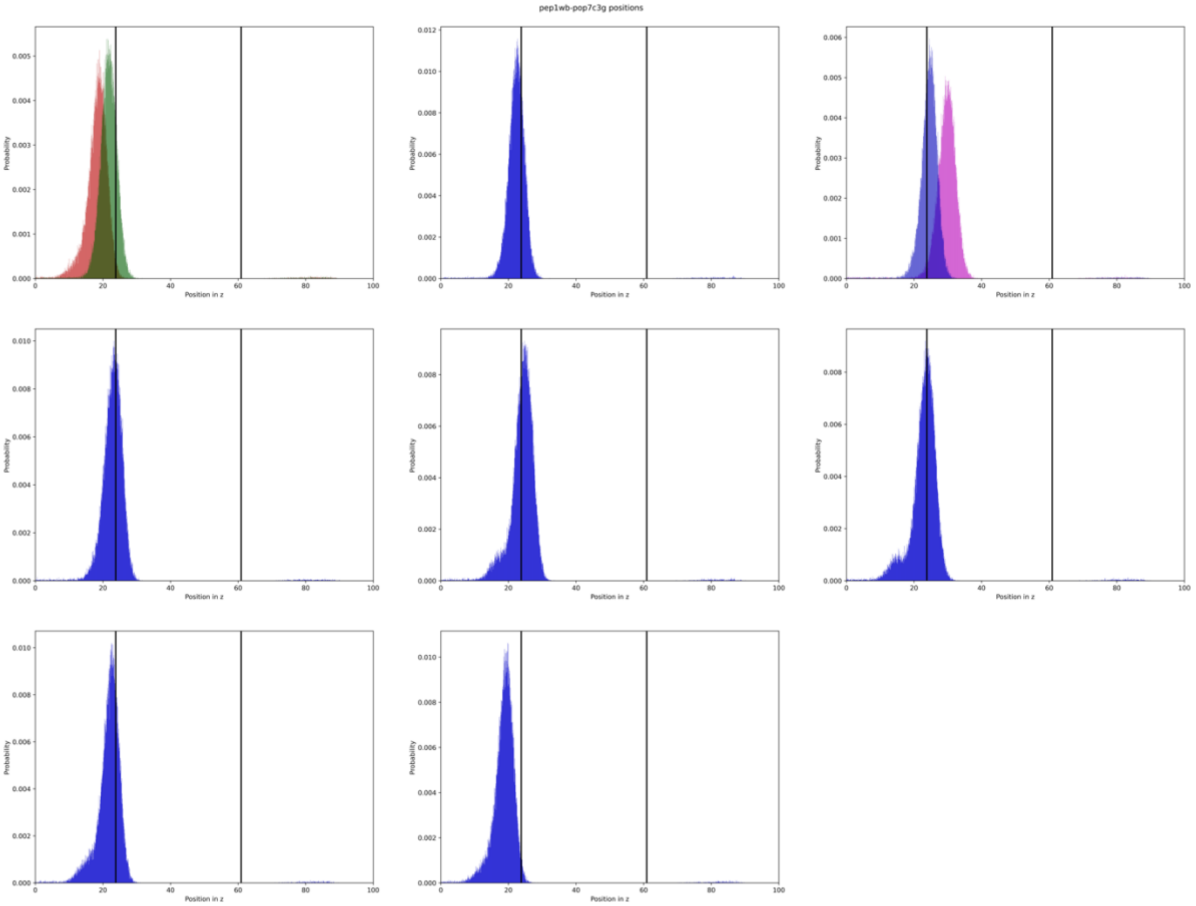

Figure S73 peptide 1 with BODIPY labelled tryptophan residue in POPC/POPG membrane

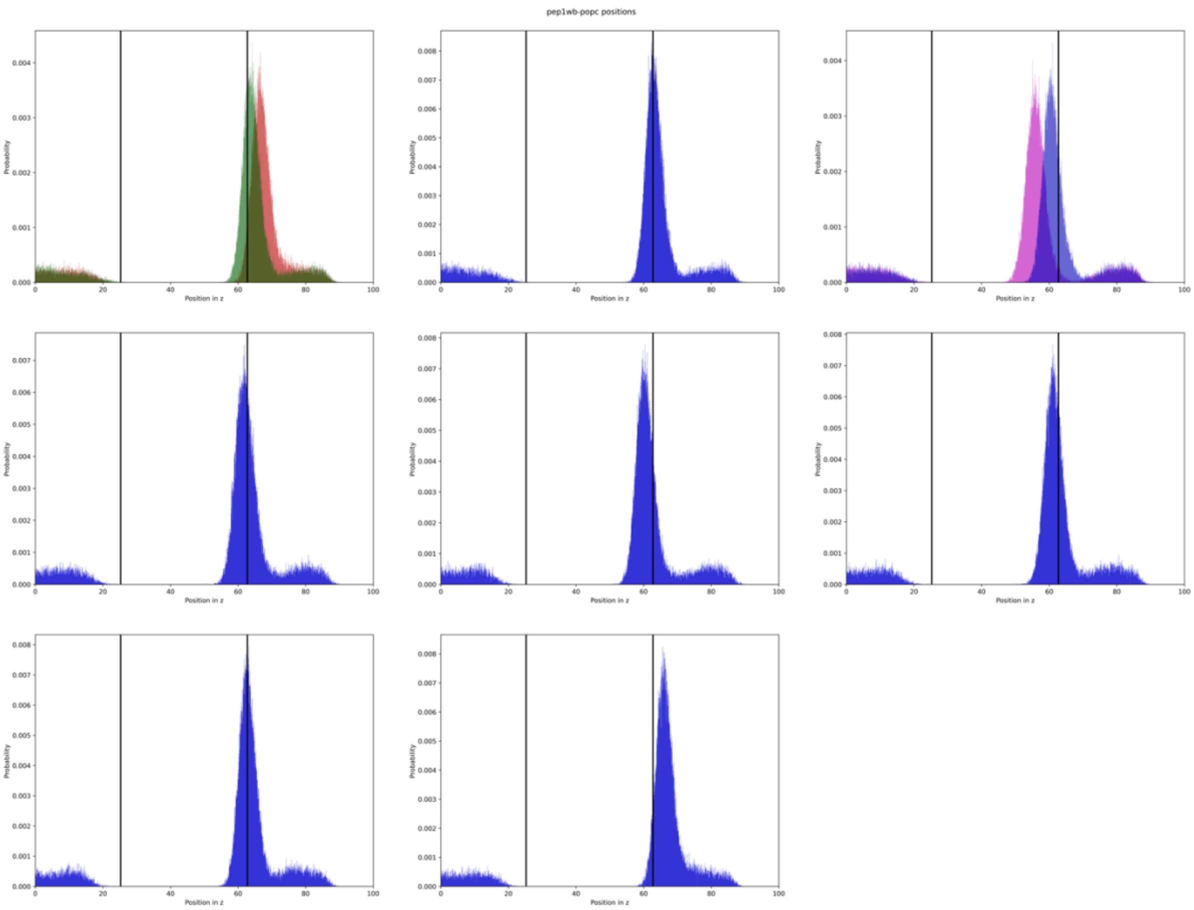

Figure S74 peptide 1 with BODIPY labelled tryptophan residue in POPC membrane

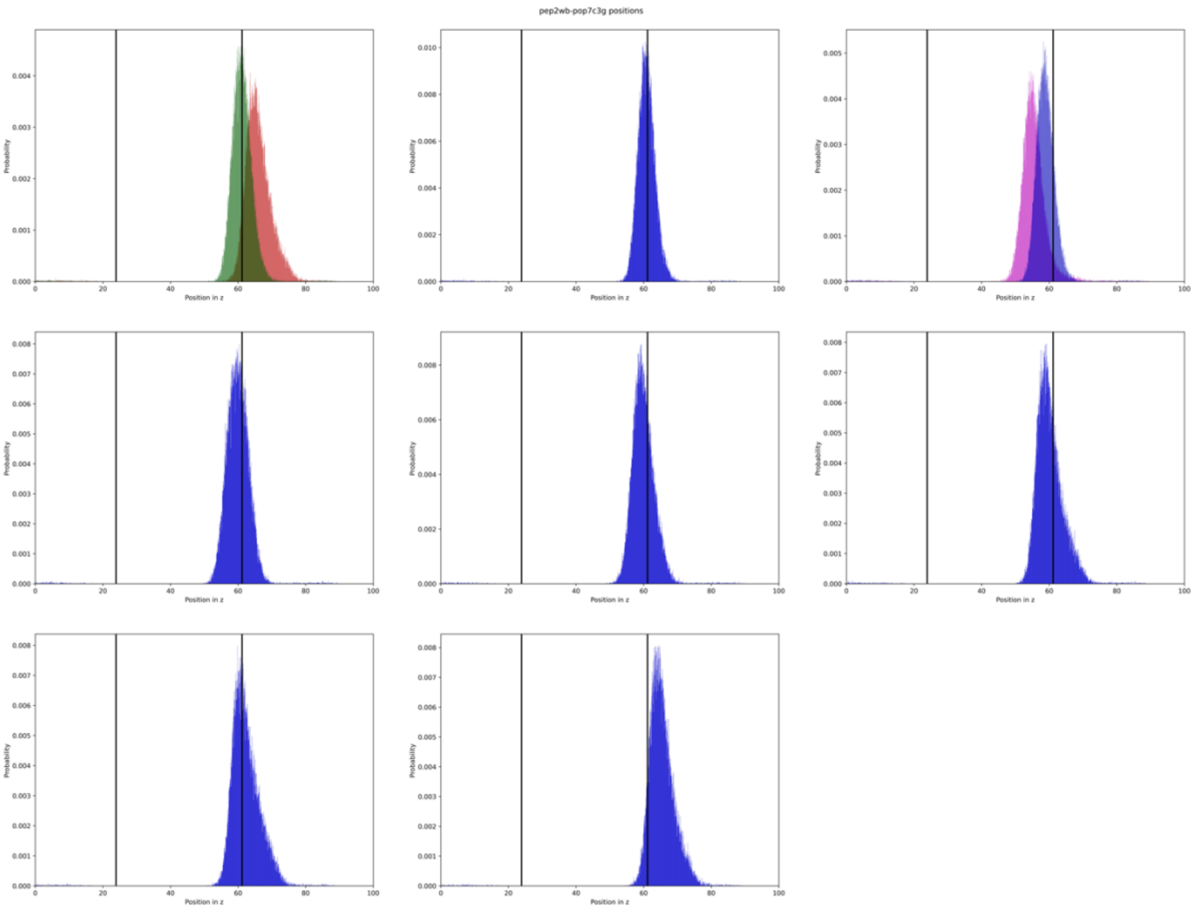

Figure S75 peptide 2 with BODIPY labelled tryptophan residue in POPC/POPG membrane

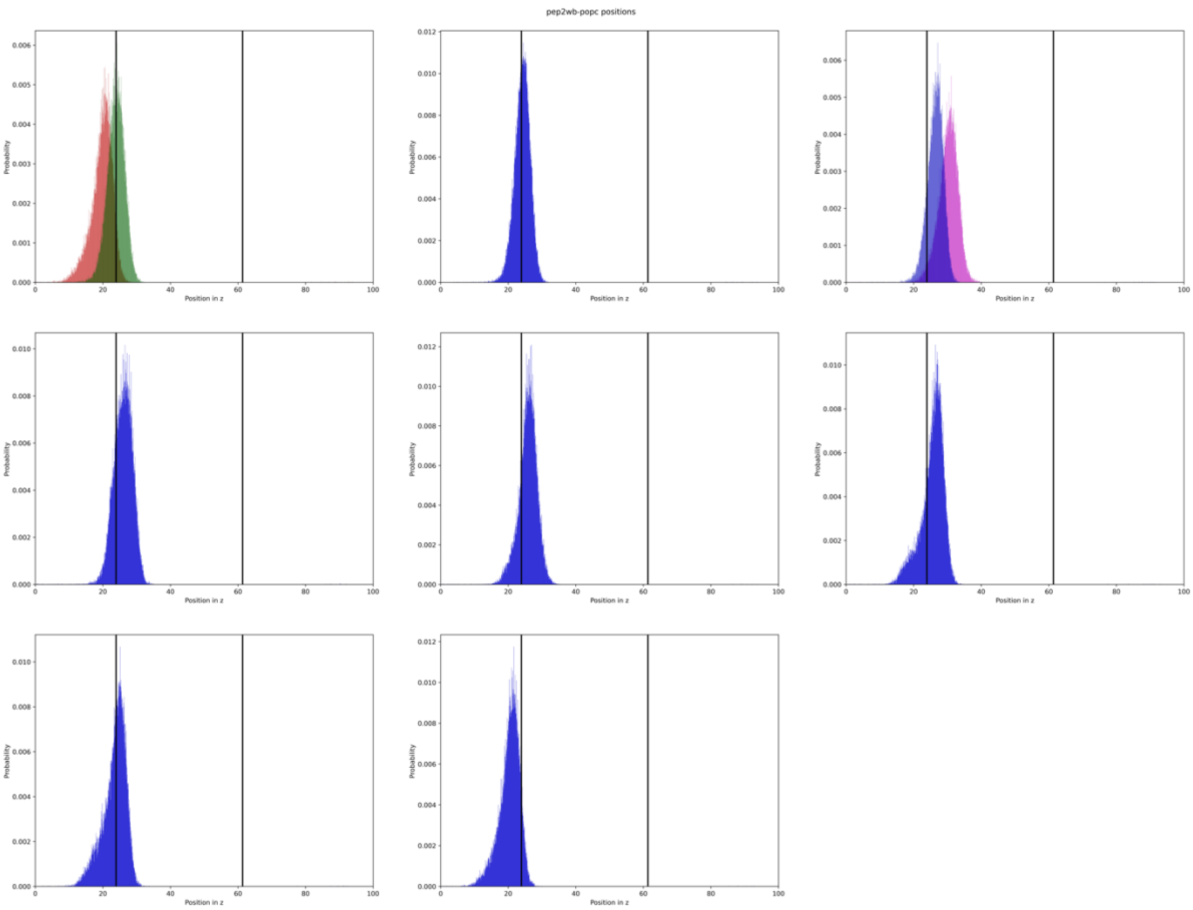

Figure S76 peptide 2 with BODIPY labelled tryptophan residue in POPC membrane

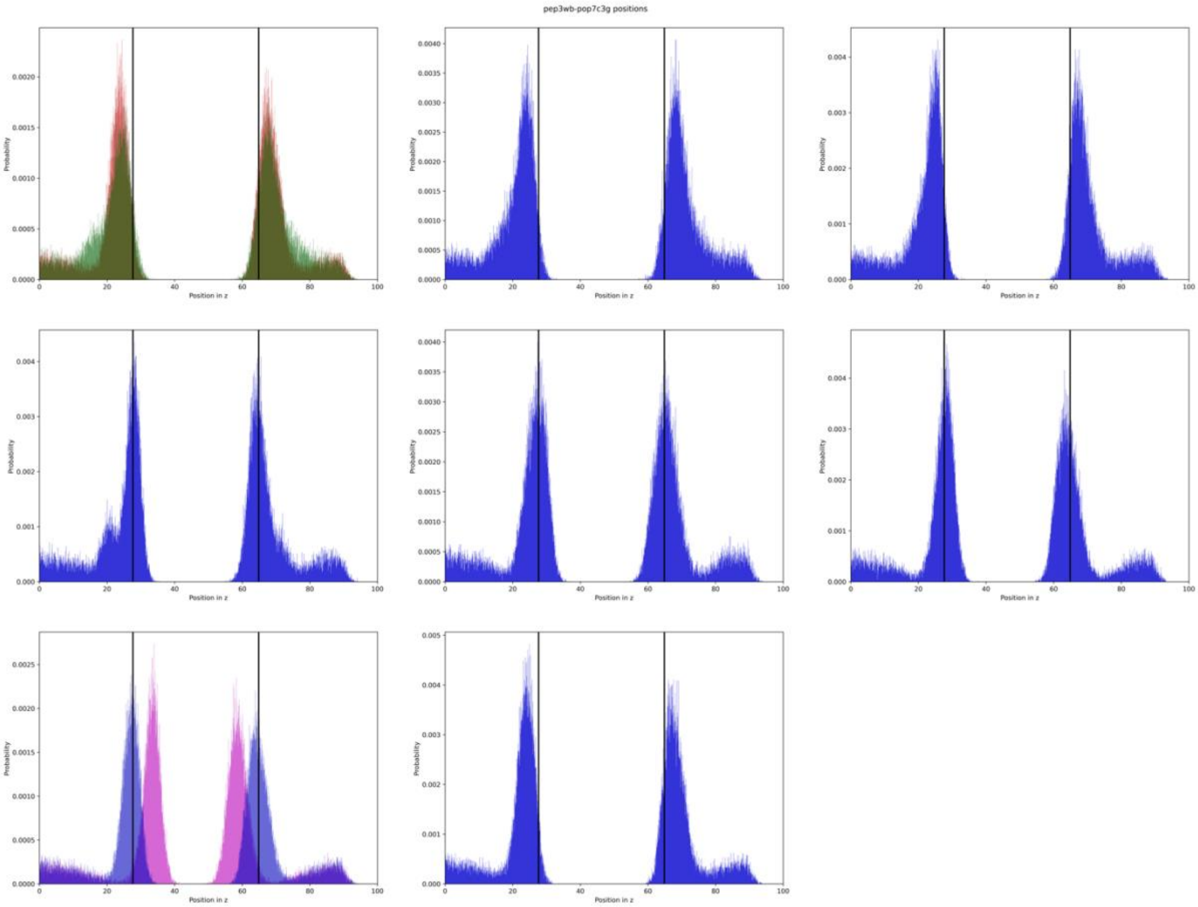

Figure S77 peptide 3 with BODIPY labelled tryptophan residue in POPC/POPG membrane

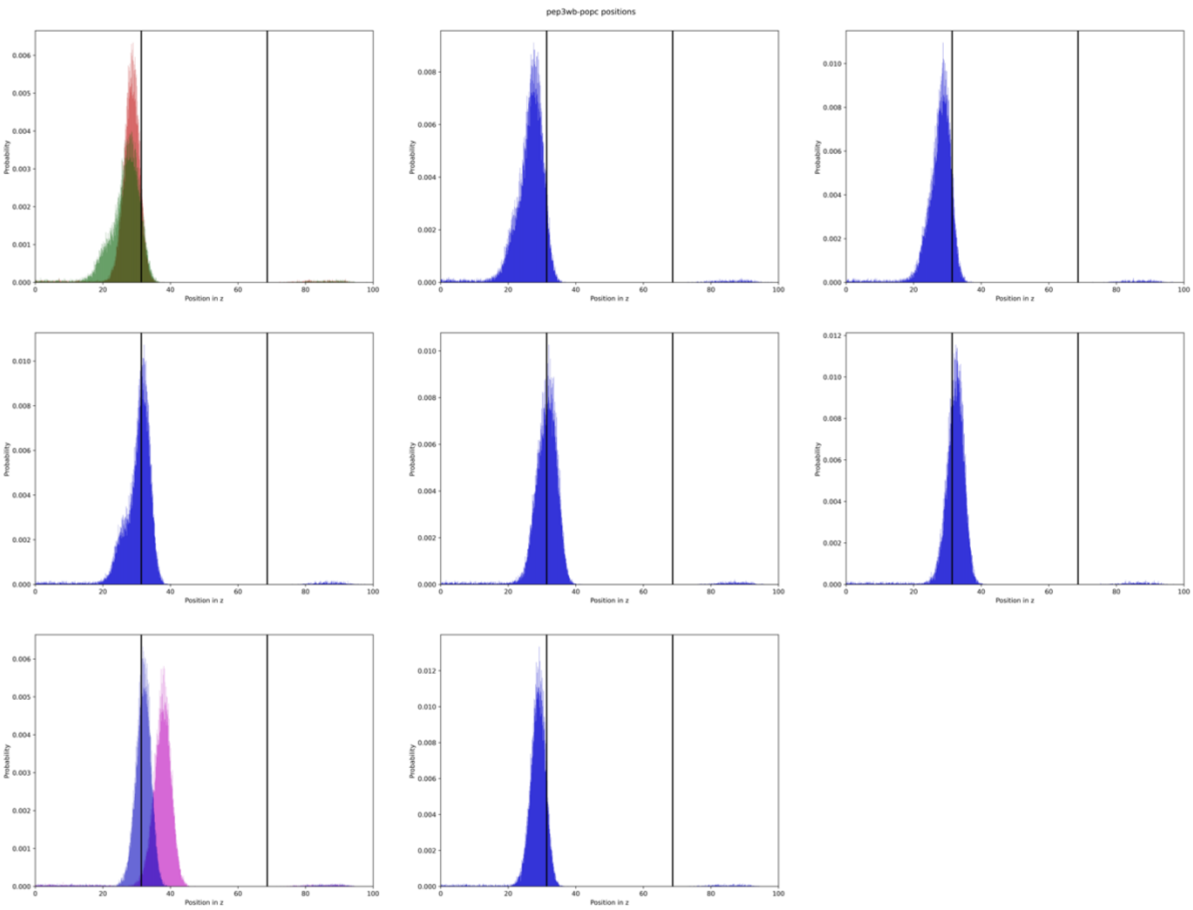

Figure S78 peptide 3 with BODIPY labelled tryptophan residue in POPC membrane

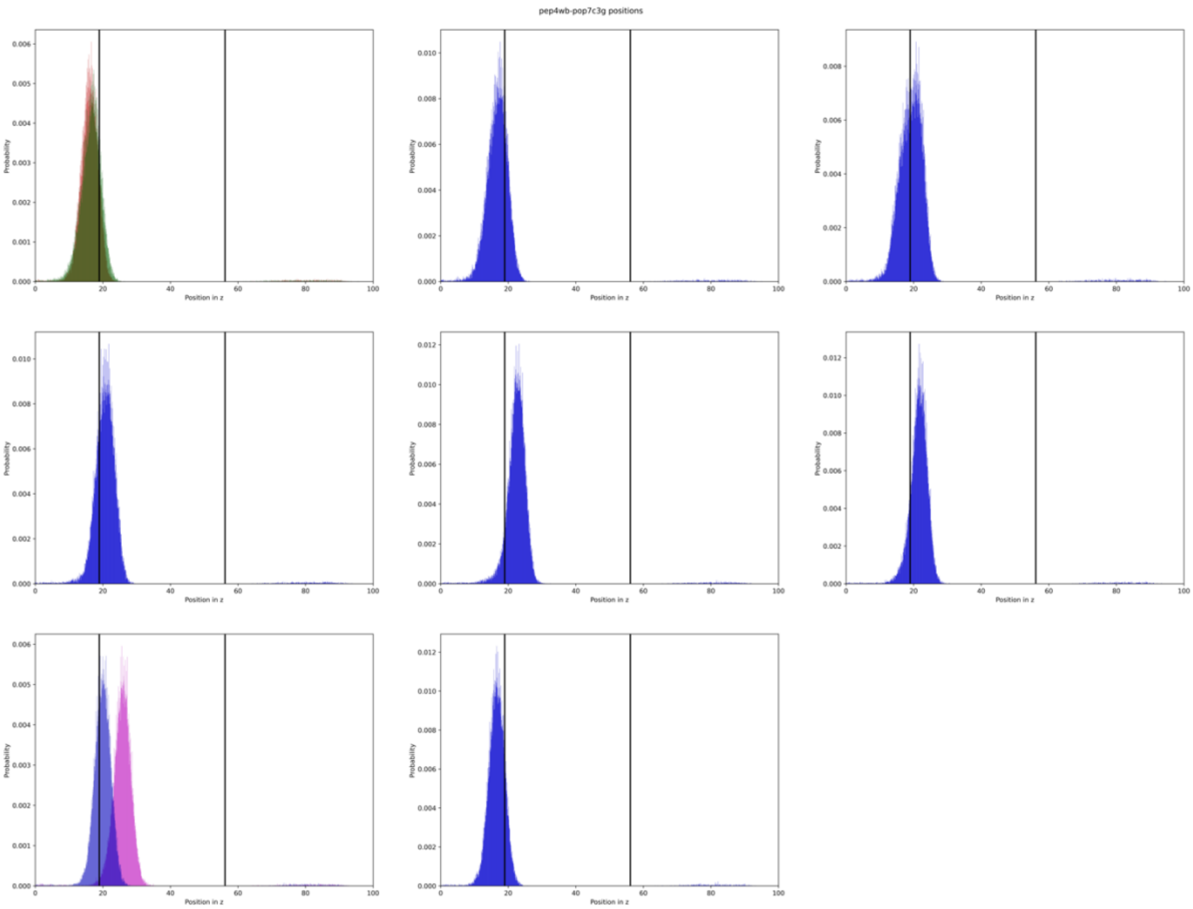

Figure S79 peptide 4 with BODIPY labelled tryptophan residue in POPC/POPG membrane

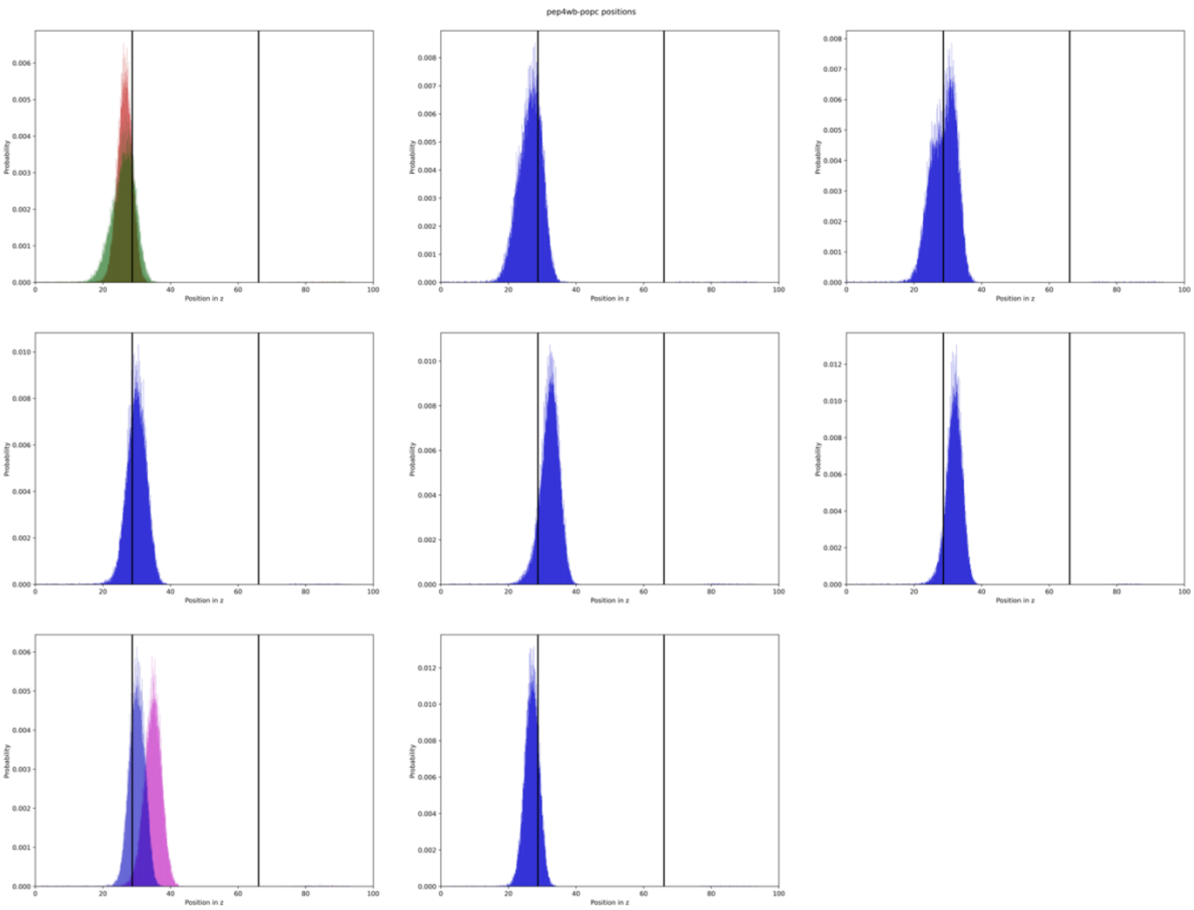

Figure S80 peptide 4 with BODIPY labelled tryptophan residue in POPC membrane

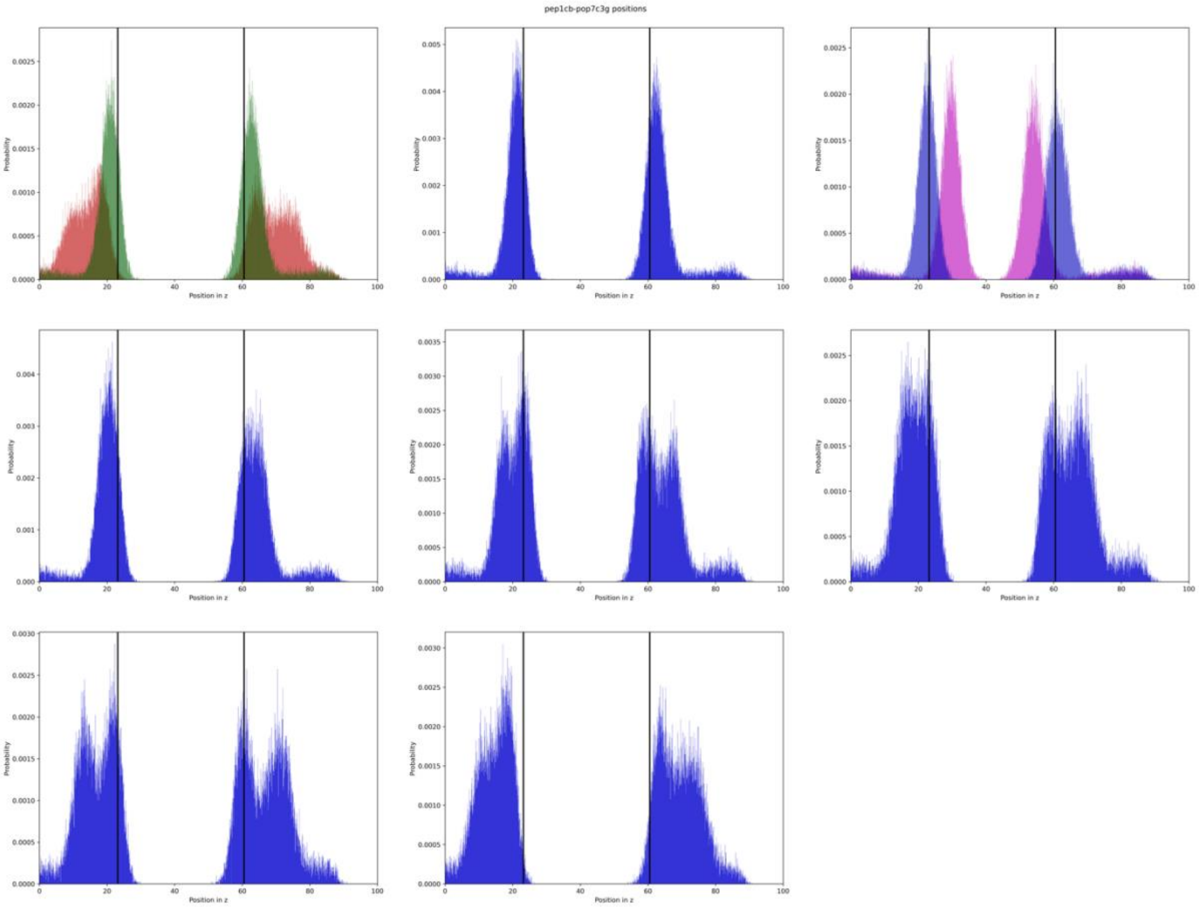

Figure S81 peptide 1 with BODIPY labelled cysteine residue in POPC/POPG membrane

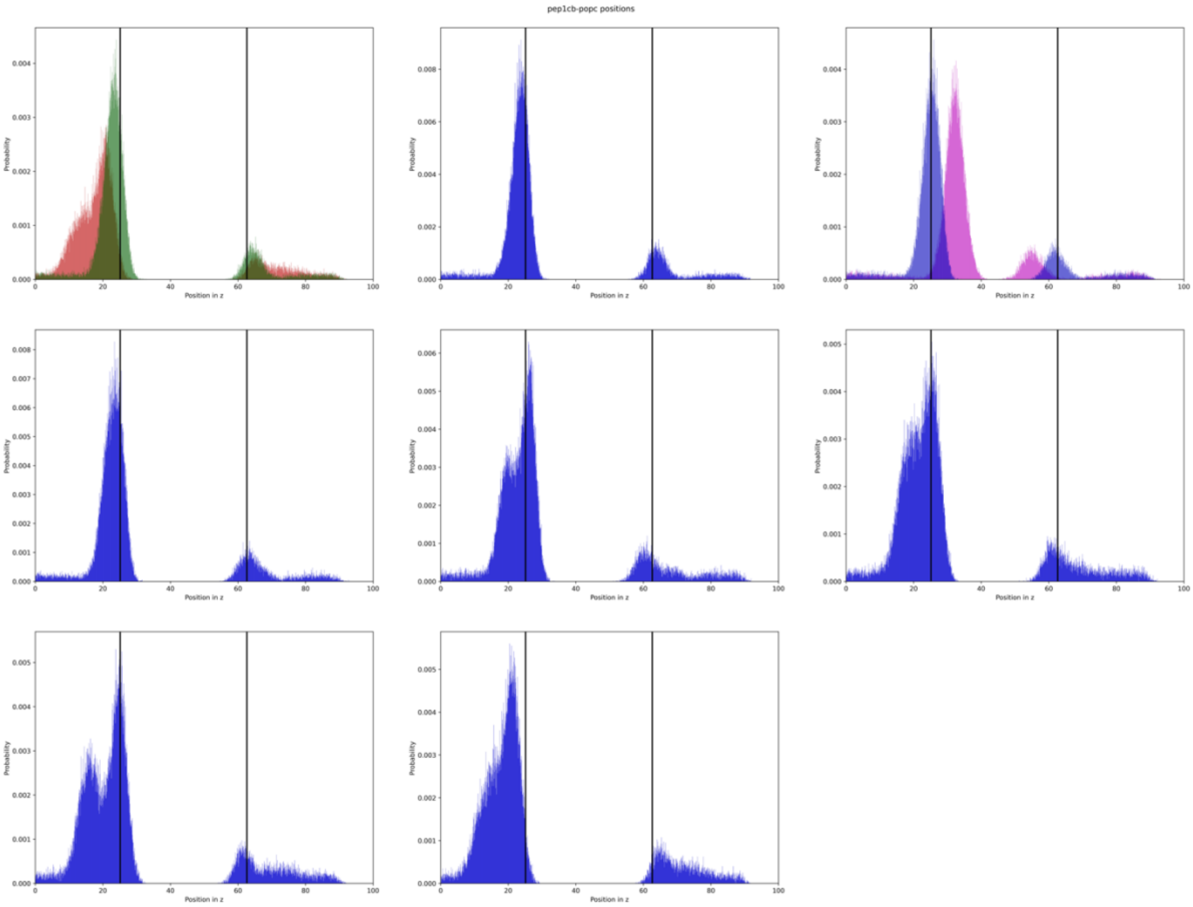

Figure S82 peptide 1 with BODIPY labelled cysteine residue in POPC membrane

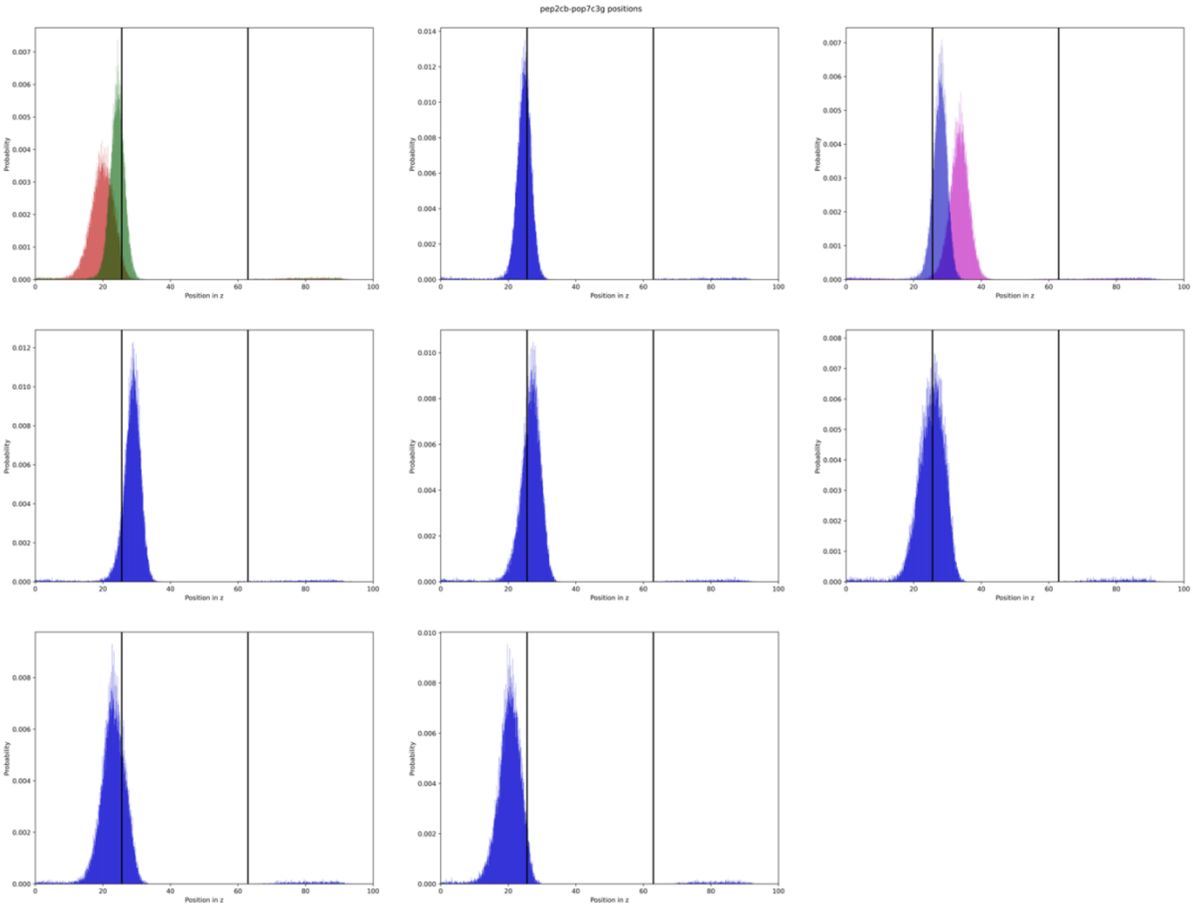

Figure S83 peptide 2 with BODIPY labelled cysteine residue in POPC/POPG membrane

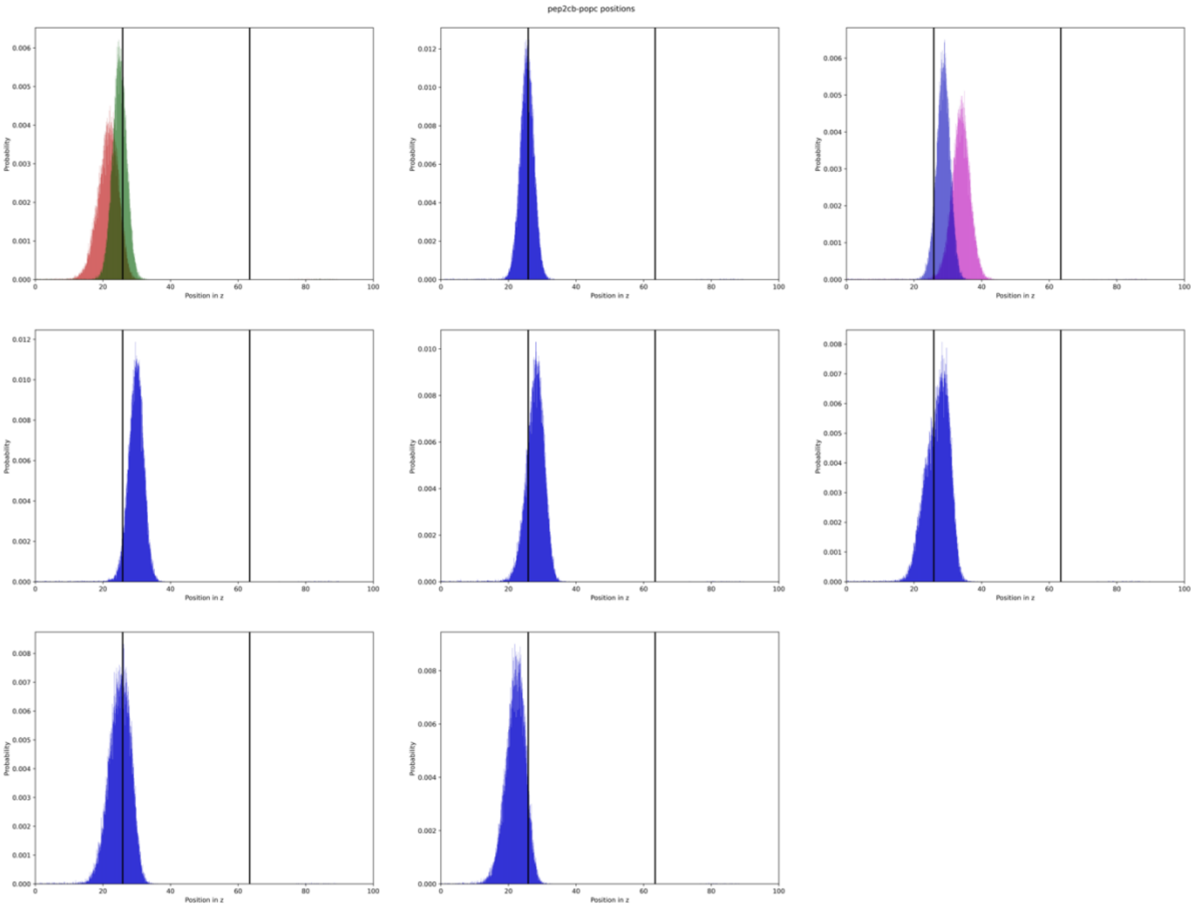

Figure S84 peptide 2 with BODIPY labelled cysteine residue in POPC membrane

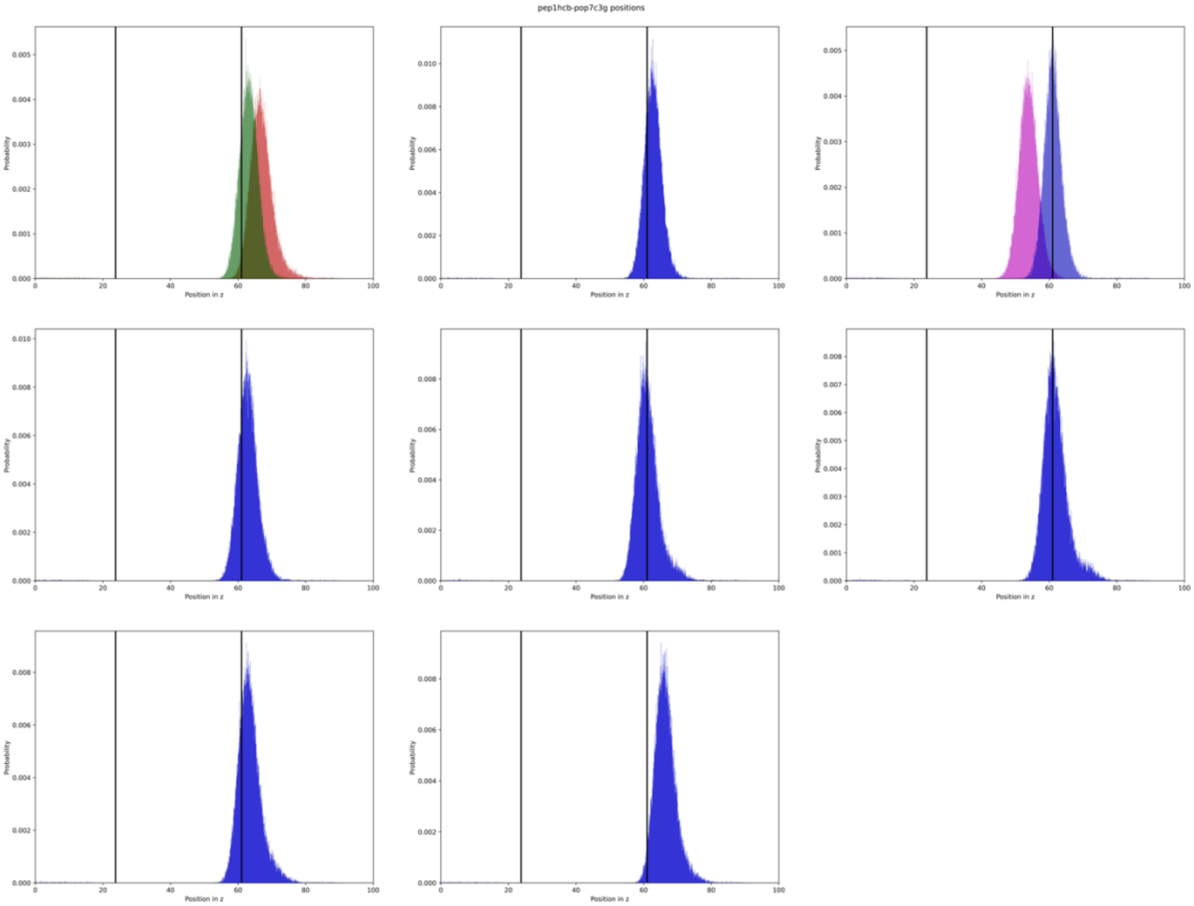

Figure S85 peptide 1 with BODIPY labelled homocysteine residue in POPC/POPG membrane

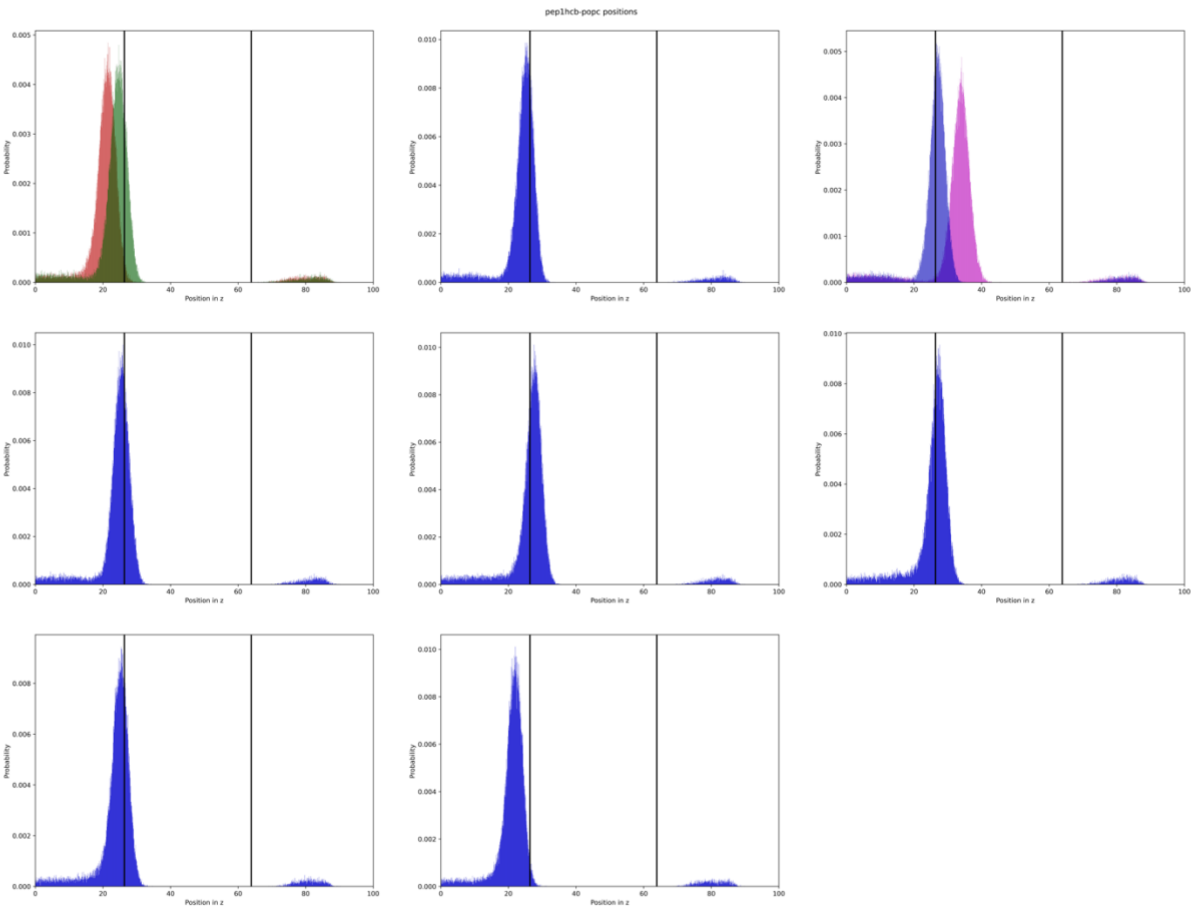

Figure S86 peptide 1 with BODIPY labelled homocysteine residue in POPC membrane

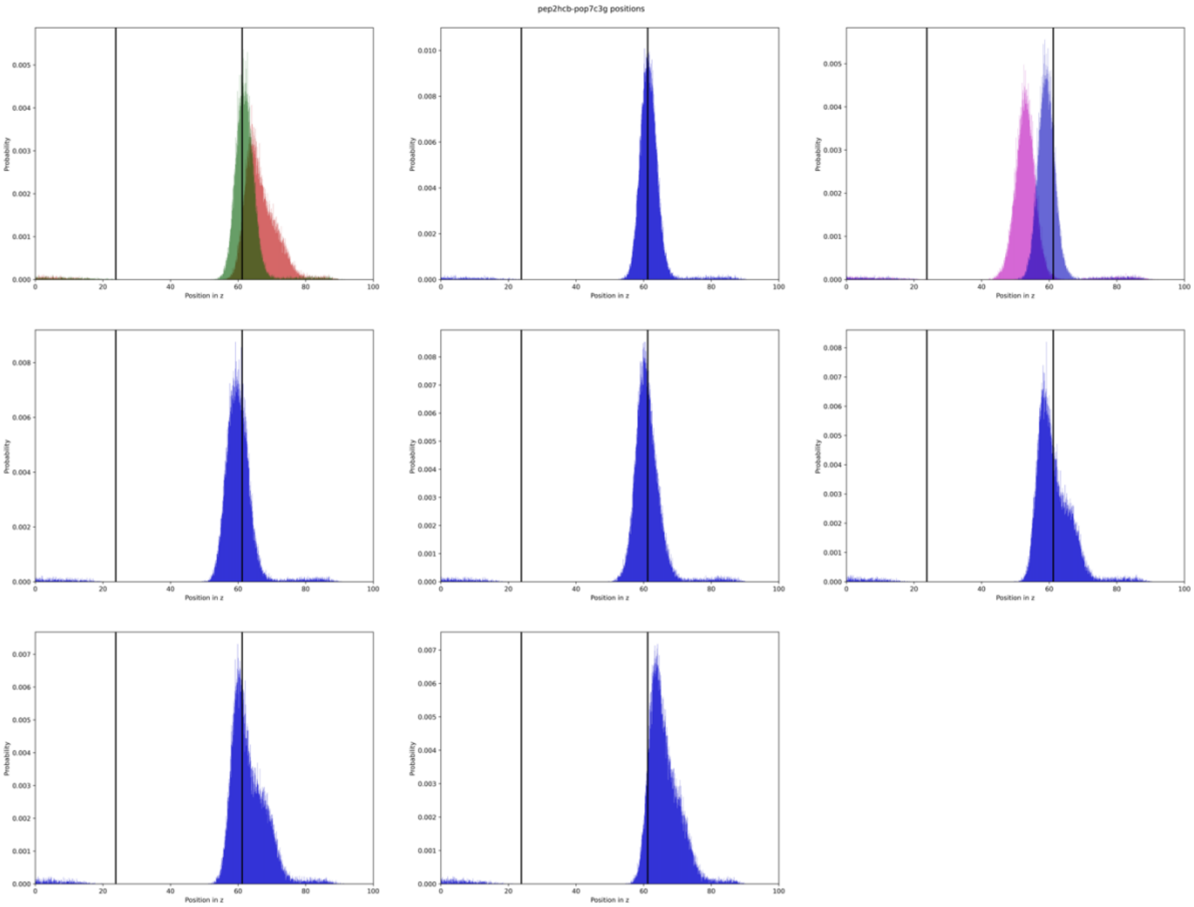

Figure S87 peptide 2 with BODIPY labelled homocysteine residue in POPC/POPG membrane

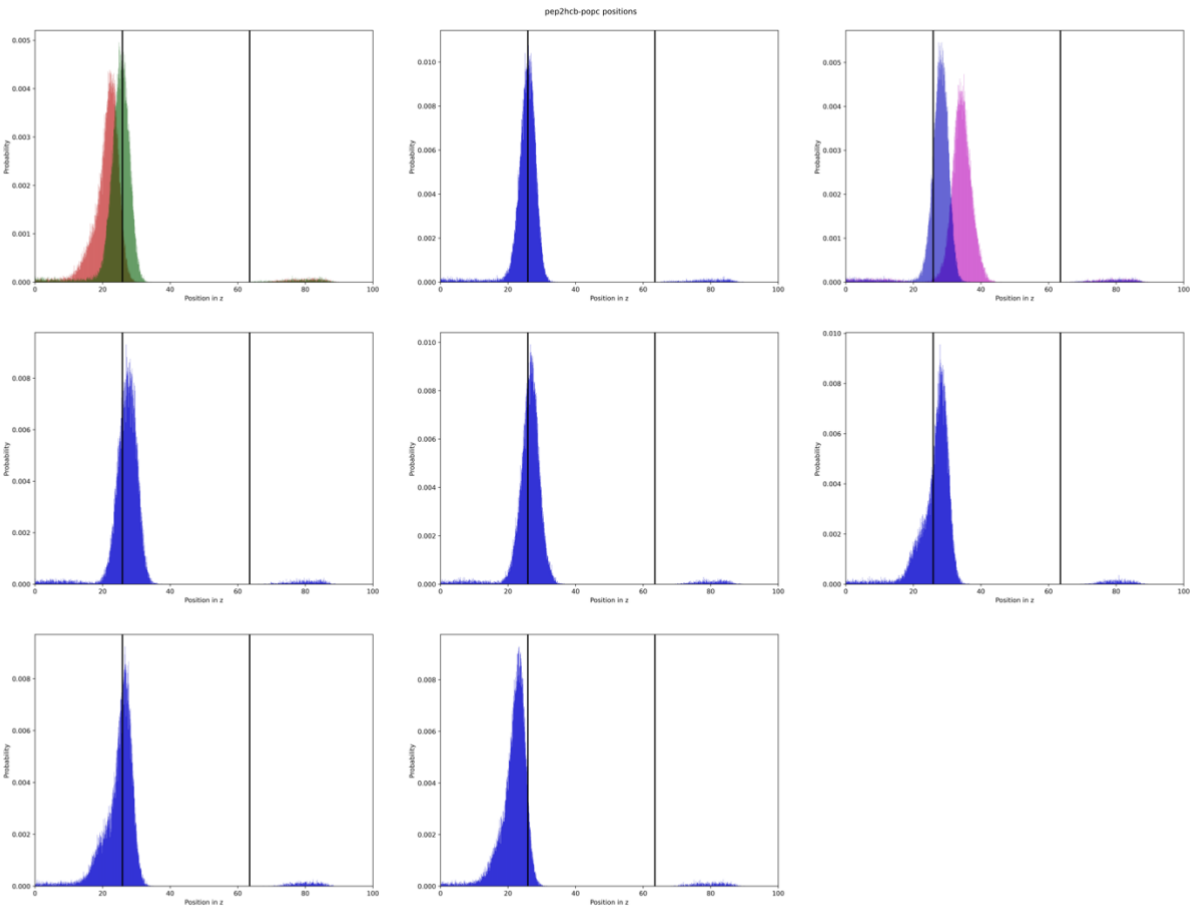

Figure S88 peptide 2 with BODIPY labelled homocysteine residue in POPC membrane

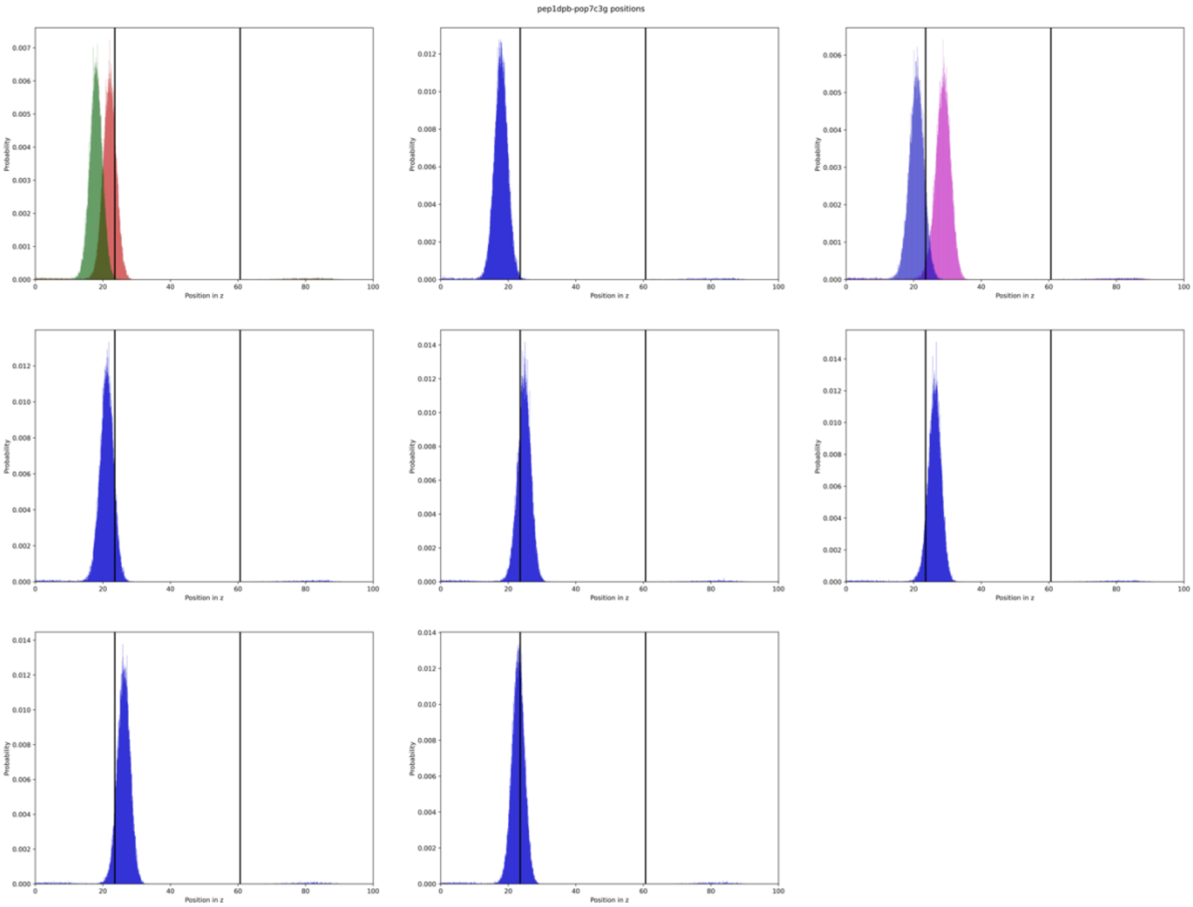

Figure S89 peptide 1 with BODIPY labelled 3-amino-Alanine residue in POPC/POPG membrane

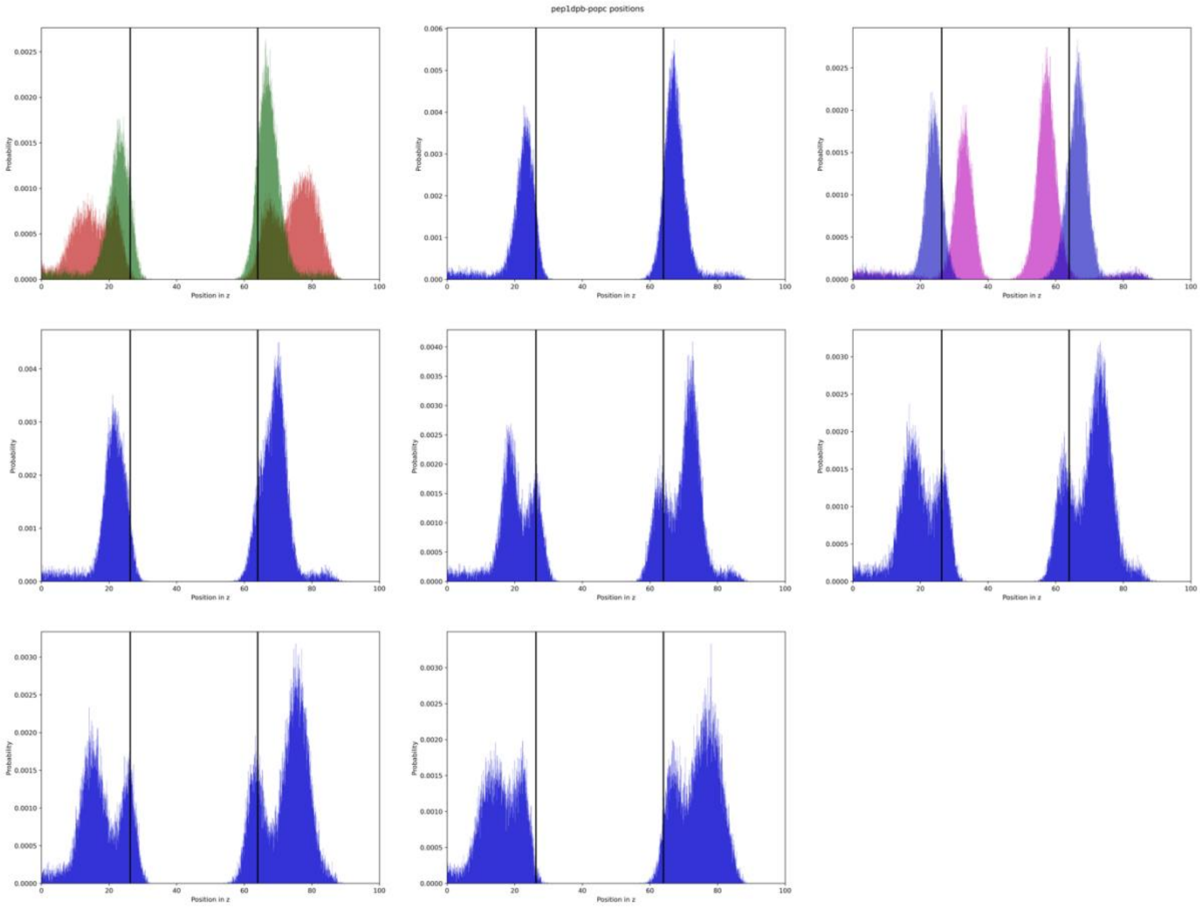

Figure S90 peptide 1 with BODIPY labelled 3-amino-Alanine residue in POPC membrane

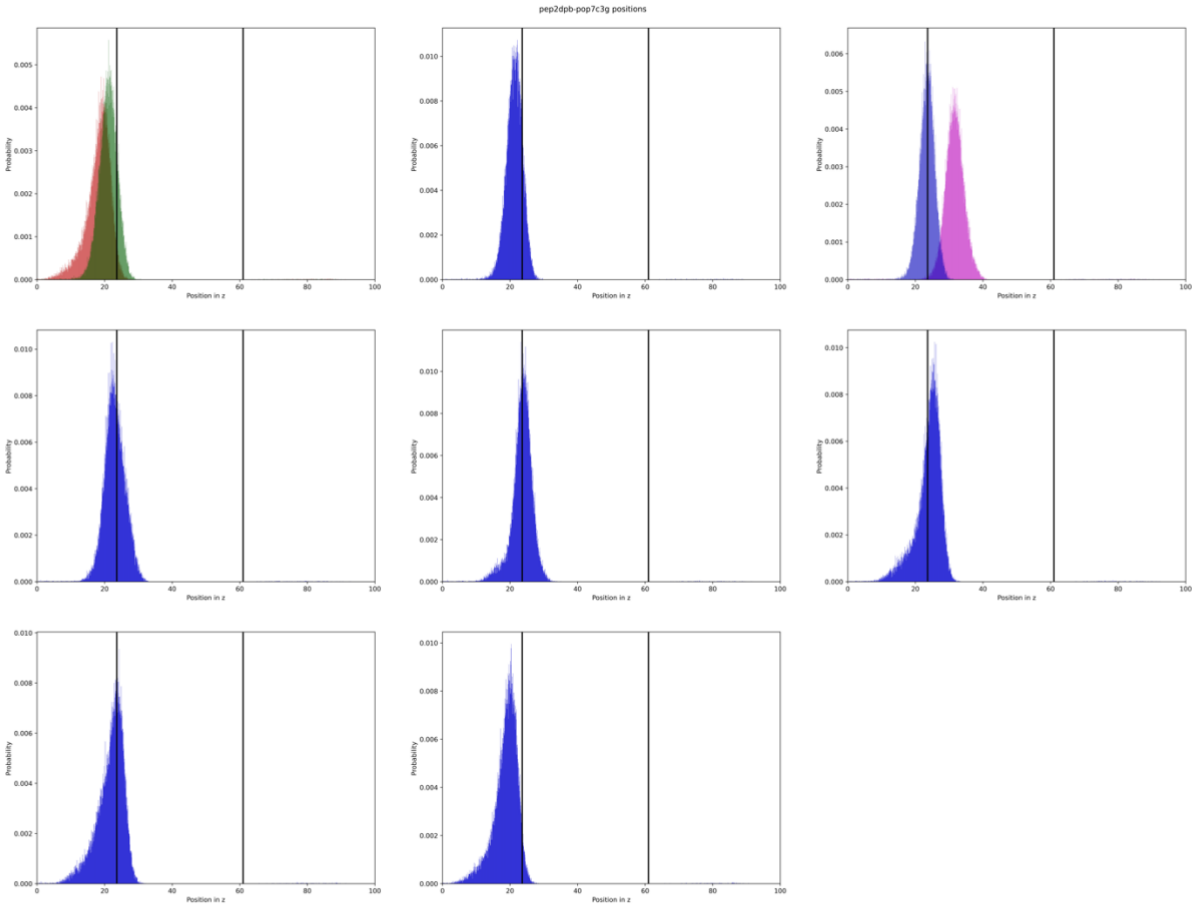

Figure S91 peptide 2 with BODIPY labelled 3-amino-Alanine residue in POPC/POPG membrane

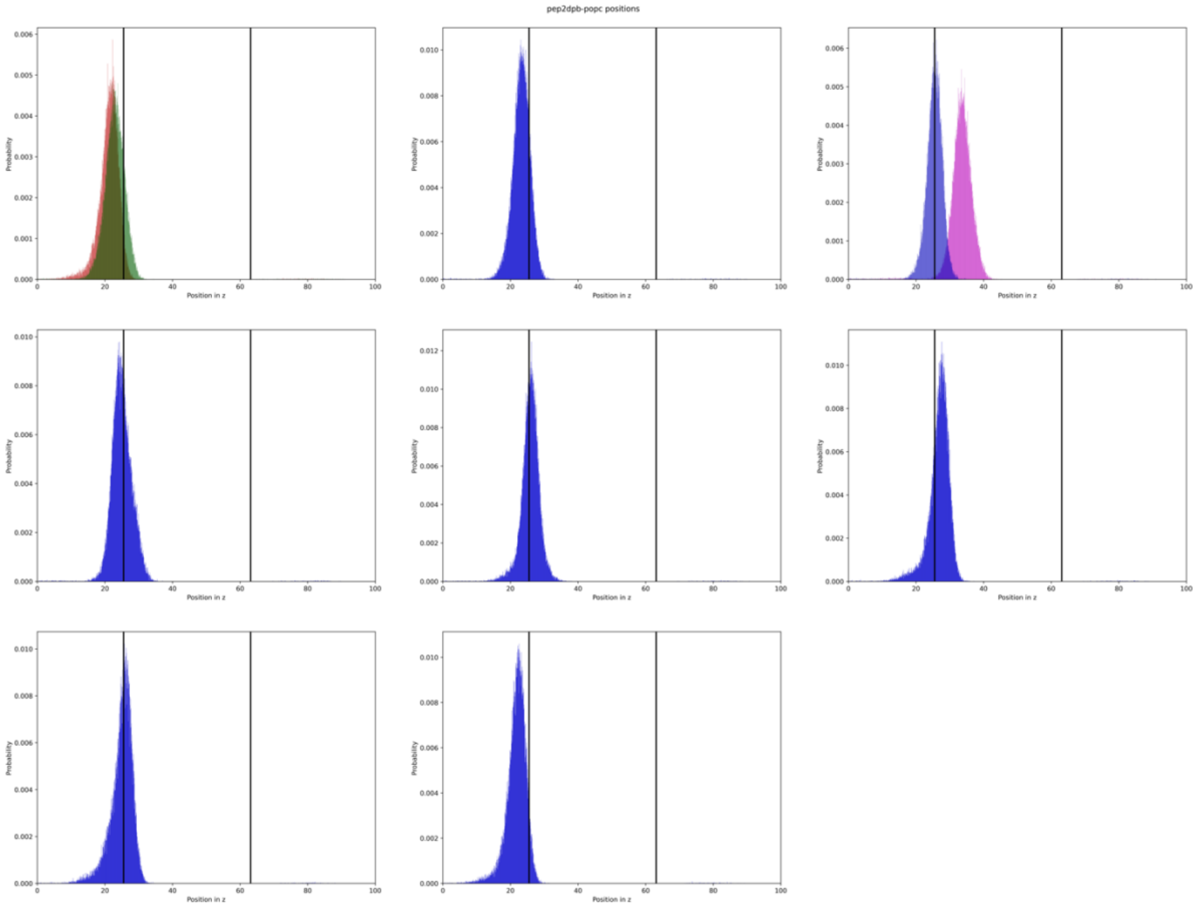

Figure S92 peptide 2 with BODIPY labelled 3-amino-Alanine residue in POPC membrane

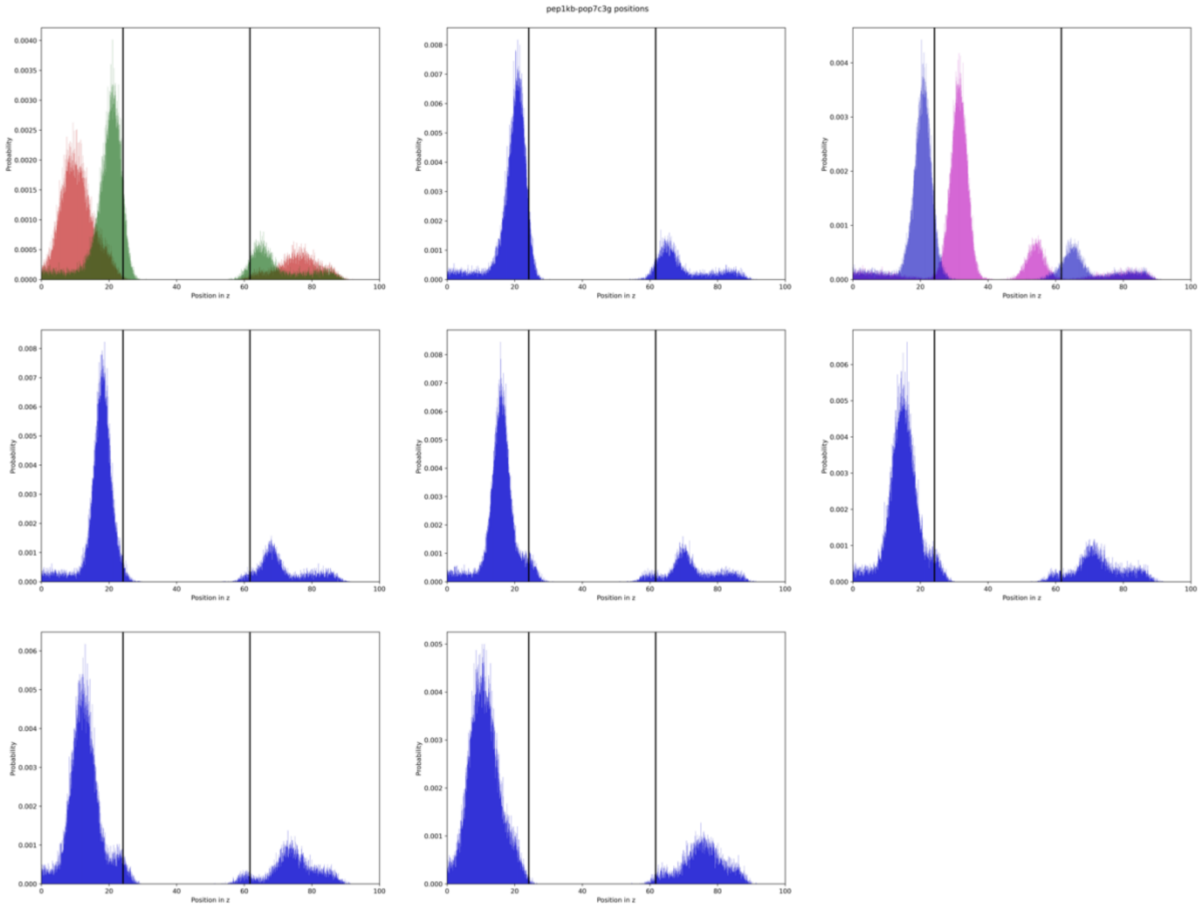

Figure S93 peptide 1 with BODIPY labelled Lysine residue in POPC/POPG membrane

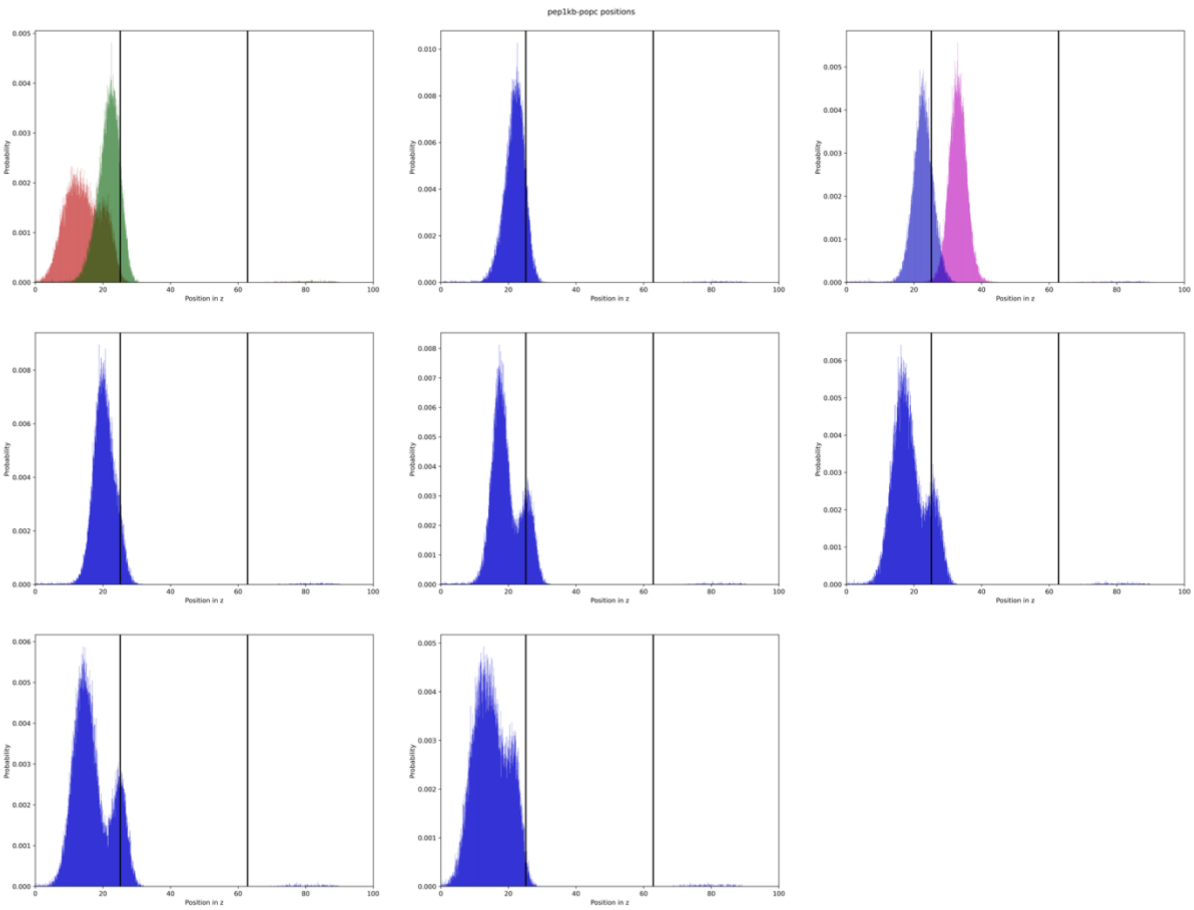

Figure S94 peptide 1 with BODIPY labelled Lysine residue in POPC membrane

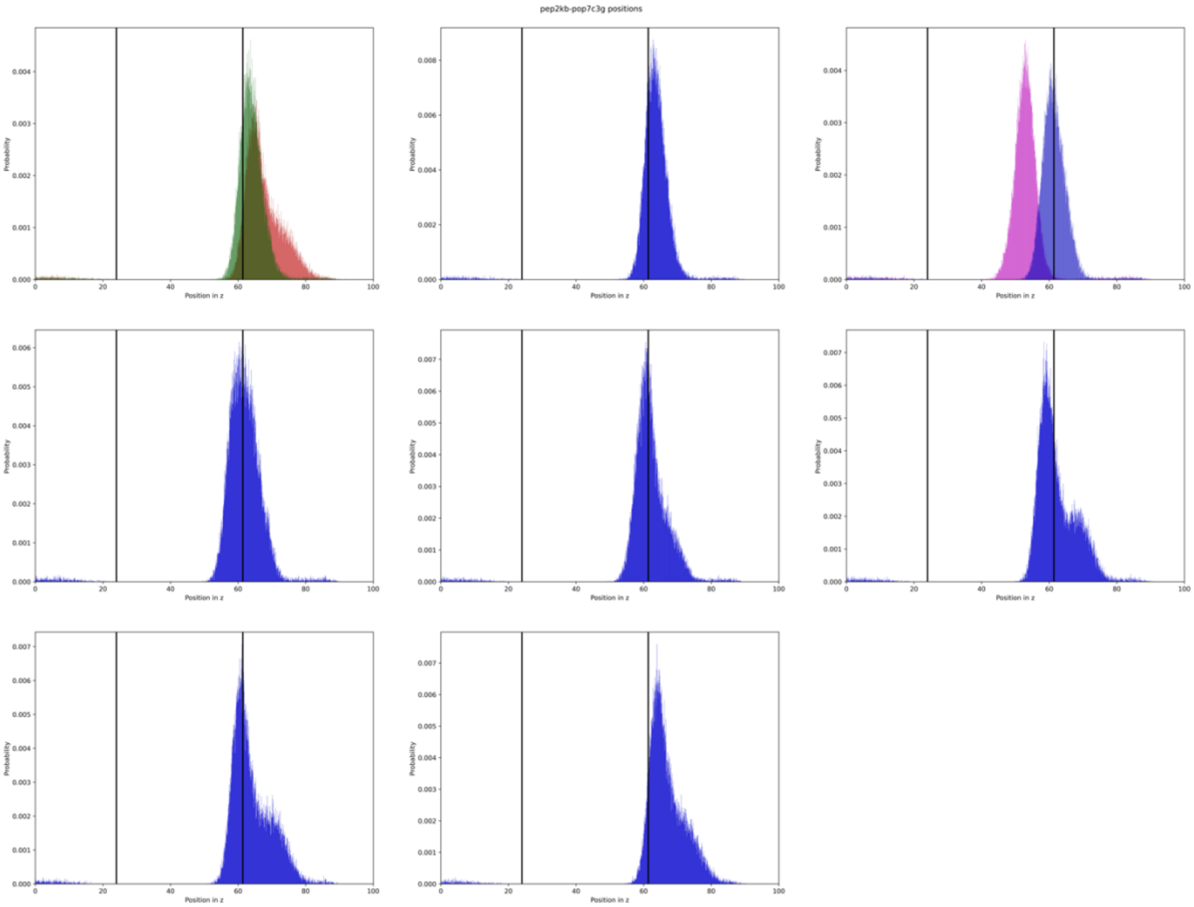

Figure S95 peptide 2 with BODIPY labelled Lysine residue in POPC/POPG membrane

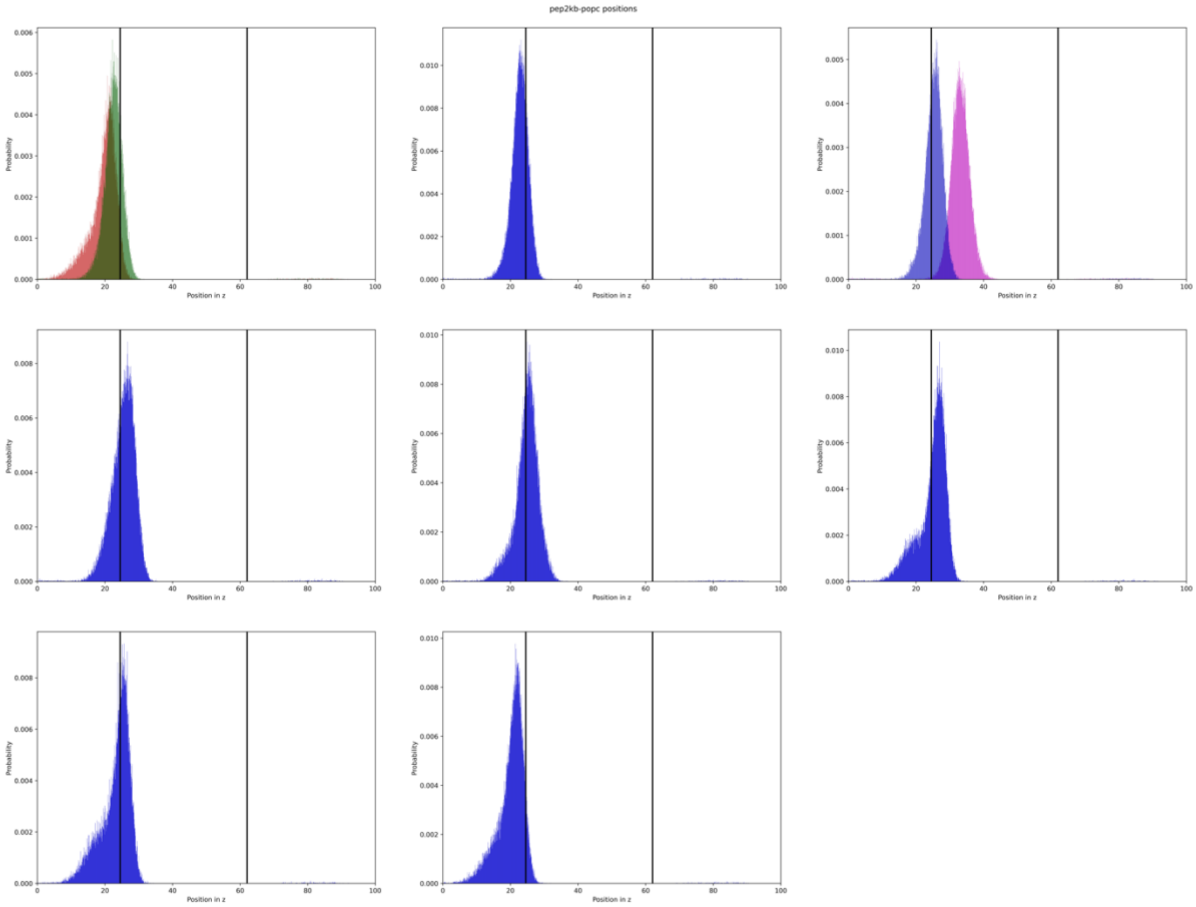

Figure S96 peptide 2 with BODIPY labelled Lysine residue in POPC membrane

References

[1] K. C. Song, P. W. Livanec, J. B. Klauda, K. Kuczera, R. C. Dunn, W. Im, *J. Phys. Chem. B* **2011**, *115*, 6157–6165.

[2] S. Kim, J. Lee, S. Jo, C. L. Brooks, H. S. Lee, W. Im, *J. Comput. Chem.* **2017**, *38*, 1879–1886.

[3] S. Jo, T. Kim, V. G. Iyer, W. Im, *J. Comput. Chem.* **2008**, *29*, 1859–1865.
